# PKCα regulates the secretion of PDL1-carrying small extracellular vesicles in a p53-dependent manner

**DOI:** 10.1038/s41419-025-07341-5

**Published:** 2025-01-14

**Authors:** Ren Zhang, Weilin Liao, Xi Chen, Junyi Wang, Jiaqi Li, Geer Chen, Weiyu Wu, Xiaoxuan Wang, Yao Zhang, Ziyu Chen, Xiaoyu Zhu, Zicong Lin, Yizhun Zhu, Lijuan Ma, Haijie Yu

**Affiliations:** 1 School of Pharmacy, Faculty of Medicine & State Key Laboratory of Quality Research in Chinese Medicine, Macau University of Science and Technology, Macau, China; 2 Dr. Neher’s Biophysics Laboratory for Innovative Drug Discovery, State Key Laboratory of Quality Research in Chinese Medicine, Macau University of Science and Technology, Macau, China; 3 Guangdong Second Provincial General Hospital, Postdoctoral Research Station of Basic Medicine, School of Medicine, Jinan University, Guangzhou, China

**Keywords:** Calcium signalling, Non-small-cell lung cancer

## Abstract

Small extracellular vesicles (sEVs), carrying PD-L1, have been implicated in immune evasion and tumor progression. However, understanding how PD-L1 sEVs are secreted still needs to be improved. We found that the secretion dynamics of PD-L1 sEVs is similar to that of other sEVs. Intracellular calcium and the associated downstream PKC signaling plays pivotal roles in releasing PD-L1 sEVs in non-small cell lung cancer cells (NSCLC). Particularly, we observed that knocking down PKCα has profound impacts on PD-L1 sEVs secretion, especially in the resting state and in the activated state, when induced by an intracellular calcium rise. Furthermore, our study revealed that PKCα regulates PD-L1 expression and PD-L1 sEVs secretion by influencing STAT1 phosphorylation and nuclear translocation in a p53-dependent manner. Notably, p53 can regulate STAT1 phosphorylation and nuclear localization, but it does not affect PKCα expression. This suggests that PKCα plays a significant role in regulating PD-L1 expression. Our findings suggest that targeting PKCα to modulate PD-L1 dynamics in NSCLC may be a promising therapeutic strategy to enhance the efficacy of immunotherapeutic interventions.

## Introduction

Extracellular vesicles (EVs), encompassing lipid bilayer-enclosed structures, are released by cells and harbor crucial cellular components. They play a pivotal role in shaping the extracellular microenvironment of tumors and facilitate intercellular communication [1]. EVs are typically classified into ectosomes and exosomes based on their size and pattern of formation [2]. Exosomes, also referred to as small EVs (sEVs), stand out due to their smaller dimensions. Within cells, sEVs take the form of intraluminal vesicles (ILVs) residing within multivesicular bodies (MVBs). Upon the merging of MVBs with the plasma membrane (PM), sEVs undergo secretion into the extracellular space [3]. The impact of sEVs on various biological processes has attracted considerable attention, even though the precise mechanisms underlying their secretion remain incompletely understood.

Programmed cell death-ligand-1 (PD-L1) functions as a crucial component of a specific immune checkpoint by interaction with the programmed cell death protein-1 [4]. In tumor cells, increased expression of PD-L1 correlates with higher aggressiveness and mortality, mostly attributed to PD-L1’s capacity to induce immune escape [5]. Recently, PD-L1 has been identified as a component of sEVs. PD-L1 on small extracellular vesicles (sEV-PD-L1) seemingly recapitulates the effect of PD-L1 on the cell surface. Notably, sEV-PD-L1 can be abundantly secreted into the circulation, exerting a systemic immunosuppressive impact throughout the entire body [6]. The process involved in loading PD-L1 onto sEVs may be linked to the process of sEV biogenesis [7]. However, it remains unknown whether PD-L1 shares similar mechanisms of biogenesis, cargo trafficking and secretion with other sEVs markers.

Cytosolic calcium functions as a vital second messenger, participating in numerous essential biological processes. Elevated intracellular calcium levels play a pivotal role in recruiting ‘endosomal sorting complexes required for transport’ (ESCRT), subsequently leading to an increased secretion of sEVs [8, 9]. The alterations in calcium signaling within cancer cells have been extensively investigated, encompassing aspects such as tumor angiogenesis, the regulation of the tumor microenvironment, and even enhancing tumor cell chemoresistance [10, 11]. Specifically, calcium signaling has been identified as a critical factor in the transcription of PD-L1 [12]. There is ample evidence supporting the notion that calcium flux blockade could be a potential strategy for PD-L1 inhibition [13–15]. However, the regulatory mechanism of calcium signaling on sEVs, especially sEV-PD-L1, remains incompletely understood.

Amidst the intricate web of calcium signaling, protein kinase C (PKC) emerges as an essential kinase participating in multiple signal transduction systems. Among the PKC family, conventional PKCs require diacylglycerol and calcium for their calcium-dependent activation. PKCα, an isoform of conventional PKCs, is typically activated by translocation and tight binding to plasma membranes, followed by exposing the binding sites of target substrates for their phosphorylation [16]. Its activation plays pivotal roles in signal transduction cascades, impacting cellular proliferation, differentiation, and migration processes [17, 18] of relevance for tumor therapy. PKCα was found to be associated with early and late endosomes by colocalization analysis [19], indicating a potential involvement of PKCα in the trafficking of intracellular vesicles. Since PKCα has the ability to influence calcium-dependent cellular events [20], further investigation should be focus on the impact of PKCα on PD-L1 sEVs.

In this study, we used florescent reporters to confirm the predominant distribution of intracellular PD-L1 in sEVs and to demonstrate that PD-L1 vesicle release paralleled that of sEVs. We also developed crucial roles of calcium and PKCα in PD-L1 sEVs release. Further, PD-L1 expression was regulated by PKCα-mediated phosphorylation of STAT1 in a p53-dependent manner. These findings provide critical insights into the complex regulatory network governing sEV-PD-L1 dynamics and offer valuable perspectives on the role of calcium signalling and its downstream effector, PKCα, in immune evasion and tumor progression. Our results constitute a significant advance of our understanding of PD-L1 immunotherapy and its potential clinical applications.

## Result

### PD-L1 is highly correlated with sEVs markers

**Fig. 1:**
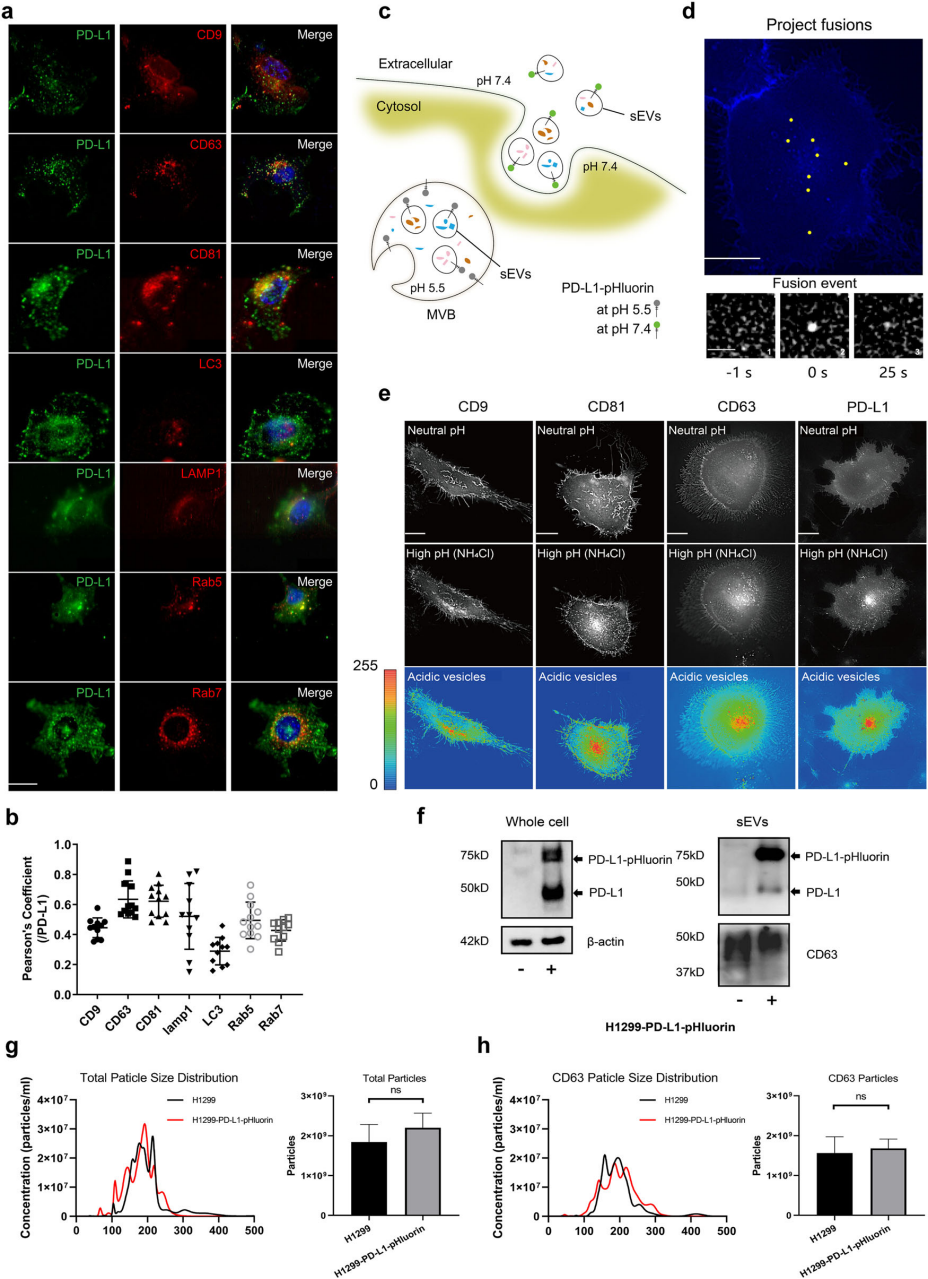
Visualization and analysis of PD-L1 localization and fusion dynamics. **a** Colocalization analysis was conducted using LBS light-sheet microscope imaging. H1299 cells were co-transfected with PD-L1-EGFP (green) along with mCherry-CD9, CD63-mCherry, mCherry-CD81, Lamp1-mScarlet, mCherry-LC3, RFP-Rab5, and RFP-Rab7 (red). Hoechst 33342 was employed for nuclear localization. Bar, 20 µm. **b** Pearson’s coefficient analysis was performed to assess the colocalization of PD-L1 with the aforementioned markers. Each group consisted of a minimum of 11 individual tests. **c** Mechanistic model for visualizing the fusion between multivesicular bodies (MVBs) and the plasma membrane (PM), the fluorescence of a pH-sensitive optical reporter (PD-L1-pHluorin) is suppressed when exposed to acidic lumen of MVB. However, upon fusion, the acidic pH within the lumen is rapidly neutralized resulting in a sudden surge in fluorescent intensity. sEVs small extracellular vesicles. **d** Upper panel: total projection of MVB-PM fusion events (yellow spots) of MVB-PM fusion events (yellow spots) over a period of time of 3 min in single cell. Bar, 15 µm. Bottom panel: representative example of MVB-PM fusion events: (1) Pre-fusion state, showing the plasma membrane before fusion event. (2) During fusion event, illustrating the fusion process between MVB and PM. (3) Post-fusion state, right before fluorescence signal depicting the disappearance after fusion event. Bar, 1.5 µm. **e** Fluorescence images of various plasmids (PD-L1, CD63, CD9, and CD81) expressed in H1299 cells under normal and elevated intracellular pH conditions (NH4Cl superfusion). On the bottom, heat maps indicate acidic vesicles in proximity to the plasma membrane (PM) obtained under high-pH conditions. Bar, 25 µm. **f** Western blotting analysis on untransfected (−) and PD-L1-pHluorin-transfected (+) cells, as well as purified sEVs, for total PD-L1 expression. Internal control protein was included for normalization. **g** Left panel: total particle size distribution of H1299 cells and PD-L1-pHluorin-transfected cells by NTA (*n* ≥ 5). Right panel: histogram of total counts of particles with the 50–250 nm range between two cell types (*n* ≥ 5). **h** left panel: CD63 particle size distribution of H1299 cells and PD-L1-pHluorin-transfected cells by NTA (*n* ≥ 5). Right panel: histogram of total counts of CD63 particles with the 50–250 nm range between two cell types (*n* ≥ 5). Statistic data are presented as means ± SEM. *P < 0.05; **P < 0.01; ***P < 0.001 using Student’s *t* test.

While PD-L1 has been identified as distributed across cell membranes and certain organelles, its specific distribution in intracellular vesicles remains unclear. To address this, we co-transfected PD-L1 together with intracellular vesicle markers into H1299 cells (Fig. 1a). Following co-localization analysis with Pearson’s correlation coefficient (Fig. 1b), we observed that PD-L1 had the most significant colocalization with sEVs markers, particularly CD63 and CD81. In contrast, CD9 enriched in sEVs originating from plasma membrane [21], displayed slightly weaker colocalization. We observed a partial co-localization of PD-L1 with markers associated with endosomes (Rab5, Rab7) and lysosomes (LAMP1), corresponding to their roles in the processes of generation and degradation of sEVs. There was only limited co-localization with the autophagosome marker LC3 implying that PD-L1 plays only a minor role in autophagy. Based on the strong co-localization observed between PD-L1 and sEVs markers, we hypothesize that PD-L1 is present in sEVs, similar to CD63.

sEVs have their own distinct secretion dynamics from other vesicles. To further explore secretion dynamic of sEVs-PD-L1, we constructed PD-L1-pHluorin, which is similar to CD63-pHluorin [22] in order to visualize vesicle fusion with the plasma membrane (Fig. 1c). We compared the fluorescence of various pHluorin reporters expressed in H1299 cells under normal (pH = 7.4) and alkaline condition (pH > 7.4). Alkaline conditions were induced by NH_4_Cl superfusion (Fig. 1e). The distributional patterns of PD-L1 fluorescence showed similarity to those of sEVs markers. Reporters were predominantly observed on the plasma membrane, exhibiting notable enrichment within vesicles of MVBs positioned around the nucleus which is consistent with the findings reported in previous studies [23]. In H1299 cells stably expressing PD-L1-pHluorin (H1299-PD-L1-pHluorin), we observed multiple bursts of pHluorin fluorescence signals at the PM (Fig. 1d, upper and Video 1), indicating that MVBs fuse with PM (MVB-PM fusion). Time lapse imaging of such individual bursts shows sudden increases of fluorescence at the PM, which closely resembles the MVBs-PM fusion signals generated by CD63-pHluorin (Fig. 1d, bottom) [22]. Whole cell protein and protein from supernatant-derived sEVs were collected for western blot analysis (Fig. 1f) and results confirmed the rich expression of PD-L1-pHluorin in both whole cell lysates and sEVs. sEVs were further characterized using nanoparticle tracking analysis (NTA), which revealed that most vesicles ranged in size from 50 to 250 nm. No significant differences were observed in the total particle count between sEVs isolated from H1299 cells and those from H1299-PD-L1-pHluorin cells (Fig. 1g). In addition, further analysis revealed no statistically significant differences in the CD63 protein expression or the total amount of CD63-positive particles between sEVs derived from these two cell types (Fig. 1f, h).

### PD-L1 shows similarities to CD63 in MVB-PM fusion

**Fig. 2:**
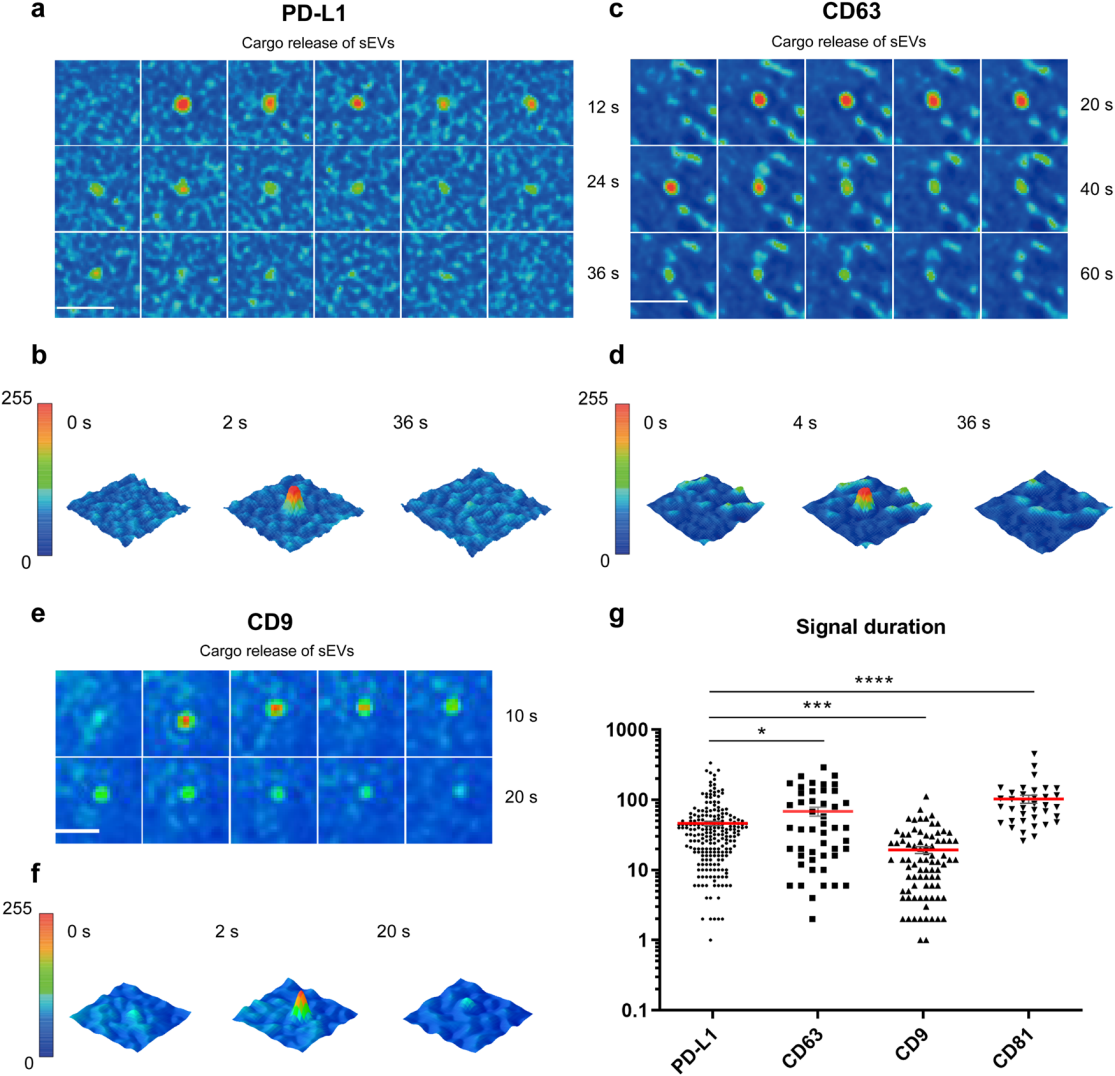
Compared the vesicle secretion dynamics of PD-L1 with others sEVs markers. **a** High-resolution time-lapse imaging (heat maps) capturing a dynamic fusion event involving the fusion of PD-L1-pHluorin-labeled sEVs proteins. Bar, 2 µm. **b** 3D heat maps illustrating distinct temporal stages of the PD-L1 fusion event: pre-fusion state, active fusion event, and post-fusion state with the disappearance of the fusion signal. **c** High-resolution time-lapse imaging (heat maps) capturing a dynamic fusion event involving the fusion of CD63-pHluorin-labeled sEVs proteins. Bar, 2 µm. **d** 3D heat maps illustrating distinct temporal stages of the CD63 fusion event: pre-fusion state, active fusion event, and post-fusion state with the disappearance of the fusion signal. **e** High-resolution time-lapse imaging (heat maps) capturing a dynamic fusion event involving the fusion of CD9-pHluorin-labeled sEVs proteins. Bar, 2 µm. **f** 3D heat maps illustrating distinct temporal stages of the CD9 fusion event: pre-fusion state, active fusion event, and post-fusion state with the disappearance of the fusion signal. **g** Fluorescent signal duration of PD-L1 (mean = 46.2 s, *n* = 215), CD63 (mean = 68.6 s, *n* = 48), CD81 (mean = 102.8 s, *n* = 37) and CD9 (mean = 19.3 s, *n* = 82) fusion events. *P < 0.05; **P < 0.01; ***P < 0.001; ****P < 0.0001 using one-way ANOVA.

Despite the high correlation between PD-L1 vesicle and sEVs, it is not known whether PD-L1 sEVs shares the same secretion dynamics as other typical sEVs. We statistically assessed the duration of burst fluorescence signals between the time-point just before a fusion event to the completion of MVB fusion with the PM, and observed longer signal duration in PD-L1 sEVs (T_PD-L1_: 46.18 s), distinct from that of synaptic vesicles (T_NPY_: 0.85 s) and dense core vesicles (T_VAMP2_: 2.12 s) (Fig. 2g and Video 2). Our findings are consistent with those of a previous study, which also reported that the secretion of sEVs is characterized by a longer signal duration compared to synaptic vesicles and dense core vesicles [22]. Notably, the duration of CD63 (T_CD63_: 68.6 s) and CD81 (T_CD81_: 102.7 s) sEV signals was longer than that of PD-L1-pHluorin (Video 3 and 4) while the duration of signals from CD9 sEVs (T_CD9_: 19.3 s) was shorter. Intriguingly, in our H1299 cells, CD9-pHluorin reporter (Video 5) appeared to exhibit extended fluorescence signals compared to those of Frederik’s study [22] in Hela cells. This observation may be attributed to differences in the compositions and components required for MVB-PM fusion in different cells. We also analyzed a specific time interval of single fusion events, starting from 1 s before the event became visible until its disappearance. We then transformed these signals into a frame-by-frame heatmap format, enabling us to visualize the fluorescence variations and duration of MVB-PM fusion. Although there were differences in the fusion duration time between PD-L1 sEVs and other sEVs, there was no significant difference in the peak fluorescence intensity during the MVB-PM fusion event (Fig. 2a–f). We conclude that the dynamics of PD-L1 MVB-PM fusion is distinct from that of synaptic vesicles and dense core vesicles but shares similarities with MVB-PM fusion dynamics of CD63.

### Calcium plays a key role in sEV-PD-L1 secretion

**Fig. 3:**
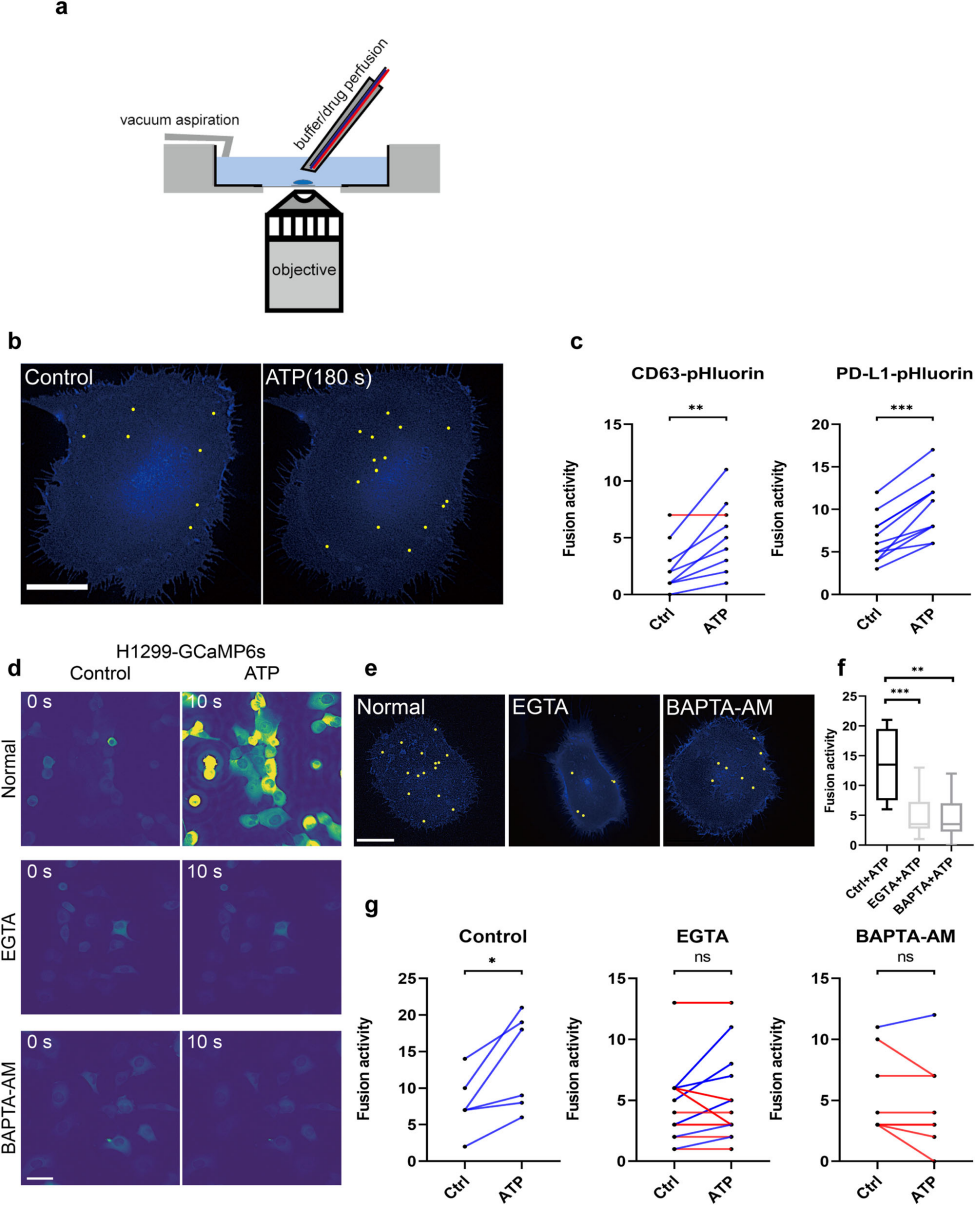
ATP-Induced PD-L1 Secretion Modulated by Calcium Ions. **a** Schematic representation of the imaging setup and drug/buffer superfusion system. **b** Cumulative representation of fusion events throughout a 180-s time course onto cells prior to (left) and following (right) stimulation with 100 µM magnesium adenosine triphosphate (ATP). Bar, 15 µm. **c** Measurement of CD63 and PD-L1 fusion activity in individual H1299 cells (*n* = 9) before and during stimulation with ATP. **d** High-resolution heat maps depicting the calcium response following ATP stimulation, utilizing the calcium fluorescent indicator protein GCaMP6s. Left panel: time point prior to ATP stimulation. Right panel: 10 s after ATP stimulation. Cells were either under control condition or calcium chelated condition. Bar, 20 µm. **e** Cumulative representation of fusion events throughout a 180-s time of Mg-ATP stimulation in cells under normal control condition or calcium chelated condition. Bar, 15 µm. **f** Quantification of basal fusion activity with ringer buffer (180 s prior to Mg-ATP stimulation) in cells under control condition or calcium chelated condition. *n* ≥ 6 individual cells per condition. **g** Quantification of fusion activity in ATP-stimulated cells under control condition or calcium chelated condition. *n* ≥ 6 cells per condition. *P < 0.05; **P < 0.01; ***P < 0.001 using Student’s *t* test. *T* tests were paired for (**c**) and (**g**). **f** One-way ANOVA, and whiskers in the box plots in (**f**) represent the highest or lowest point.

Calcium plays a pivotal role in both exocytosis and endocytosis processes, and it has been shown to promote sEVs secretion [8]. In our previous study, we observed a significant increase in the release of PD-L1 sEVs following the rise of intracellular calcium levels [15]. ATP, released as a cellular damage recognition molecule, functions as a danger signal, activating the immune system and promoting inflammation. Notably, extracellular ATP is identified as main biochemical component of the tumor microenvironment, which can promote tumor progression or suppression [24]. ATP is sensed and bound by cell surface P2Y receptors, leading to the generation of inositol trisphosphate (IP3) and subsequent increase calcium signal [25]. We utilized ATP to elevate intracellular calcium levels to examine its impact on PD-L1 sEVs. To ensure brisk local application of ATP to individual cells, we set up a superfusion system (Fig. 3a). During ATP stimulation, we observed a remarkable increase in the total projection of PD-L1 fusion events (Fig. 3b). A significant increase in fusion activity was observed in CD63-transfected H1299 cells as well (Fig. 3c). To counteract the ATP-induced increase in calcium, we employed BAPTA-AM and EGTA for chelating calcium ions (Fig. 3d and Videos 6–8). After the chelation of calcium, there was a significant reduction in fusion activity within ATP stimulation (Fig. 3e–g). These findings suggest that elevated calcium levels are crucial for ATP-induced PD-L1 sEVs release in H1299 cells.

### PKCα regulated the release of PD-L1 sEVs

**Fig. 4:**
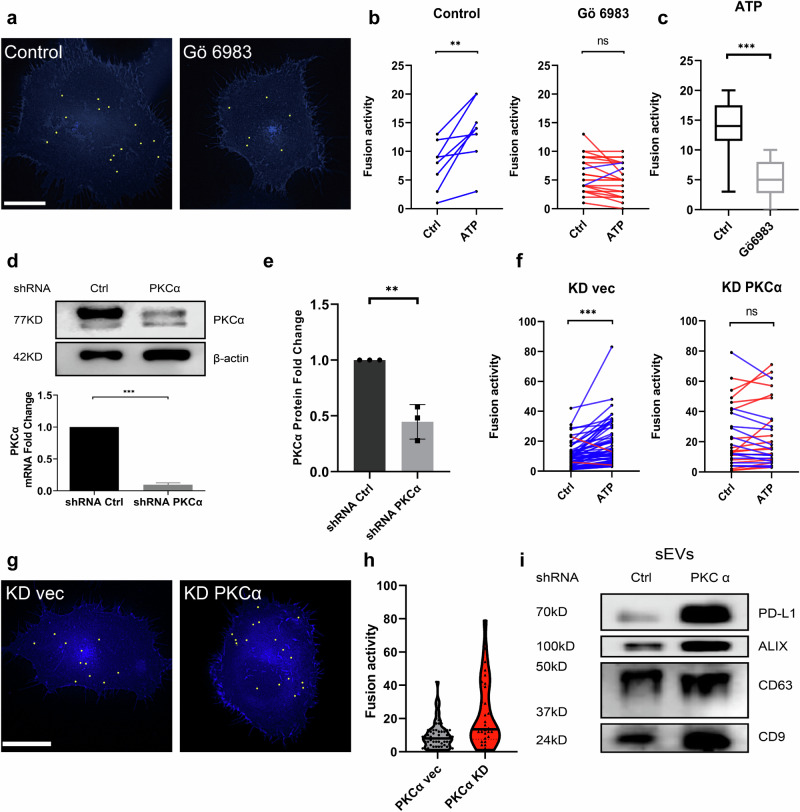
PKCα inhibition and knockdown effects on PD-L1 fusion activity in H1299 cells overexpressing PD-L1-pHluorin. **a** Cumulative representation of fusion events throughout a 180-s time of ATP (100 μM) stimulation in cells treated or untreated with protein kinase C (PKC) inhibitor Gö 6983 (1 µM). Bar, 20 μm. **b** Measurement of fusion activity in ATP-stimulated under non-treated or Gö6983 treated conditions. *n* ≥ 8 cells per condition. **c** Quantification of fusion activity with 180 s ATP stimulation in cells treated with Gö6983 or not. *n* ≥ 8 individual cells per condition. **d** Upper panel: Immunoblotting analysis confirms the reduction of PKCα expression in H1299-PD-L1-pHluorin cells following PKCα knock down. Bottom panel: Confirmation of PKCα knockdown at the mRNA level in H1299-PD-L1-pHluorin cells. data are presented as means ± SD. **e** Quantification of each blot of PKCα protein expression normalized to β-actin in H1299-PD-L1-pHluorin cells (*n* = 3). **f** Fusion activity of ATP-stimulated cells transfected with control shRNA and PKCα shRNA. *n* ≥ 3 cells per condition. **g** Cumulative representation of fusion events throughout a 180-s time at resting condition in cells transfected with control shRNA and PKCα shRNA. **h** Quantification of basal fusion activity at resting condition in cells transfected with control shRNA and PKCα shRNA. Bar, 20 μm. **i** Immunoblots for PD-L1 and sEV marker ALIX, CD63 and CD9 in sEVs isolated from cells transfected with control shRNA and PKCα shRNA. *P < 0.05; **P < 0.01; ***P < 0.001 using Student’s *t* test. *T* tests were paired for (**b**) and (**f**).

Protein kinase C (PKC) serves as an important kinase downstream of calcium elevation in the signal cascade for histamine induced MVB-PM fusion of CD63 [22]. We hypothesized that PKC and its associated pathway may serve as key elements of ATP-induced MVB-PM fusion of PD-L1. As expected, the application of the Pan-PKC inhibitor, Gö6983, resulted in a notable reduction in fusion activity induced by ATP in H1299-PD-L1-pHluorin cells (Fig. 4a–c) which suggests that the PKC pathway participate in MVB-PM fusion of PD-L1 induced by ATP.

PKCα is a calcium-dependent kinase regulating many physiological functions. Numerous investigations showed that PKCα could induce both tumor initiation and progression depending on cancer type [26]. Current studies, however, establish only poor knowledge about the link between PKCα and sEVs, particularly regarding the secretion of PD-L1 sEVs. Thus, we explored the impact of PKCα on PD-L1 sEVs. We transfected shRNA PKCα into H1299-PD-L1-pHluorin to knock down PKCα (Fig. 4d, e), and we could no longer observe the increase of PD-L1 fusion events induced by ATP (Fig. 4f). Surprisingly, we found a significant rise in the PD-L1 fusion rate under resting conditions after PKCα knockdown (Fig. 4g, h). Consistent with the increased basic fusion activity, the protein amount of PD-L1 was significantly increased in sEVs after knocking down PKCα. Furthermore, we observed an elevation in the levels of other sEVs markers (Fig. 4i). We also observed that PKCα knockdown attenuated ATP-induced fusion events in the H1975 cell line (Fig. S1a–c). However, unlike in H1299 cells, a reduction in fusion activity was noted under resting conditions following PKCα knockdown (Fig. S1d, e). Collectively, it seems that PKCα regulates PD-L1 sEVs and their release involving a complex signal cascade.

### PKCα regulates PD-L1 expression in a p53-dependent manner

**Fig. 5:**
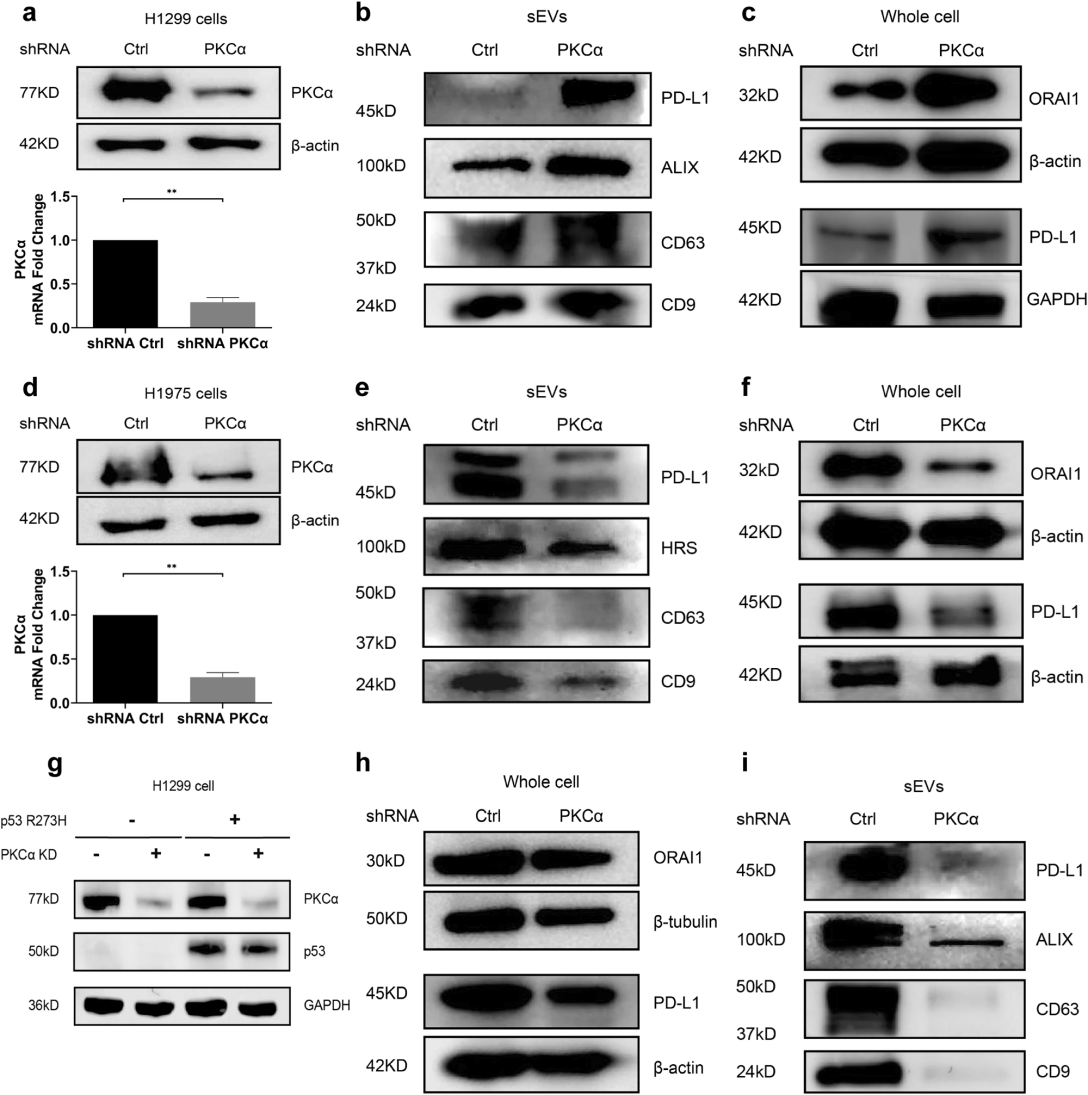
The role of p53 R273H in modulating PD-L1 expression. **a** Upper panel: Immunoblot analysis was performed on whole cell lysates from H1299 cells to validate the effectiveness of PKCα knockdown. Bottom panel: Confirmation of PKCα knockdown at the mRNA level in H1299 cells. Data are presented as means ± SD. **b** Immunoblots for PD-L1 and sEV markers ALIX, CD63 and CD9 in sEVs isolated from H1299 cells transfected with control shRNA and PKCα shRNA. **c** Immunoblot analysis was performed on PD-L1 and ORAI1 protein expression in whole cell lysates from H1299 cells to assess changes following PKCα knock down. **d** Upper panel: Immunoblot analysis was performed on whole cell lysates from H1975 cells to validate the effectiveness of PKCα knockdown. Bottom panel: Confirmation of PKCα knockdown at the mRNA level in H1975 cells. Data are presented as means ± SD. **e** Immunoblots for PD-L1 and sEV markers HRS, CD63 and CD9 in sEVs isolated from H1975 cells transfected with control shRNA and PKCα shRNA. **f** Immunoblot analysis was performed on PD-L1 and ORAI1 protein expression in whole cell lysates from H1975 cells to assess changes following PKCα knock down. **g** Immunoblot analysis was performed on PKCα and p53 protein expression in whole cell lysates from H1299 cells to validate the effectiveness of overexpress p53 R273H. **h** ORAI1 and PD-L1 protein expression were determined in p53 R273H overexpression H1299 cells transfected with control shRNA and PKCα shRNA. **i** Immunoblots for PD-L1 and sEV markers ALIX, CD63 and CD9 in sEVs isolated from p53 R273H overexpression H1299 cells transfected with control shRNA and PKCα shRNA. *P < 0.05; **P < 0.01; ***P < 0.001 using Student’s *t* test.

Building upon our earlier observations centered around H1299-PD-L1-pHluorin cells, where PD-L1 is overexpressed, we sought to investigate whether PKCα exerts a similar impact in native H1299 cells. Consequently, we knocked down PKCα in native H1299 cells (Fig. 5a and Fig. S4a), and found an enhanced PD-L1 expression in sEVs, concomitant with the upregulation of other sEVs markers (Fig. 5b).

To investigate the impact of PKCα knockdown on sEV-PD-L1 secretion, we compared the expression of key SNARE complex proteins (SNAP23, Syntaxin4, VAMP7) and the Munc18 family (Munc18-1, Munc18-2, Munc18-3) between H1299 WT and PKCα knockdown cells (Fig. S2a, c). No significant changes were observed in the SNARE complex (Fig. S2b), suggesting PKCα may regulate PD-L1 secretion independently of these SNARE proteins related to MVB-PM fusion. Within the Munc18 family, Munc18-2 decreased, and Munc18-3 increased (Fig. S2d). This aligns with previous findings [27, 28] showing Munc18-2 downregulation impairs ATP-induced secretion events, while Munc18-3 upregulation enhances vesicle secretion by promoting Syntaxin4 trafficking and MVB-PM fusion.

To further assess PKCα’s role, cells were treated with PMA/TPA, a widely used phorbol ester that activates PKC (Fig. S3a). Although PMA is known as a PKCα agonist that significantly increases PKCα activity, prolonged exposure can lead to a decrease in PKCα expression [29, 30]. In H1299 WT cells, PKCα levels initially rose at 30 min before declining, while in PKCα knockdown cells, both PKCα and phosphorylated PKCα (p-PKCα) were consistently reduced post-treatment (Fig. S3b). Munc18-1 expression remained stable in knockdown cells regardless of PMA treatment. While after 24 h of PMA exposure, Munc18-3 increased in both WT and knockdown cells, coinciding with reduced PKCα, suggesting that decreased PKCα activity promotes Munc18-3 upregulation, enhancing MVB-PM fusion and vesicle secretion.

In whole cell lysates, the expression of ORAI1, an important calcium channel, was upregulated upon PKCα knockdown. This upregulation of ORAI1 is potentially responsible for the heightened release of sEVs. Besides, the depletion of PKCα also resulted in elevated expression of PD-L1 in whole-cell lysates (Fig. 5c and Fig. S4b, c). Consistent with the results from PD-L1-pHluorin transfected H1975 cells mentioned earlier, a reduction of PD-L1 was noted in sEVs isolated from cell supernatants, and the expression of both ORAI1 and PD-L1 was downregulated in whole cell lysates of H1975 post PKCα knock down, contrary to our findings with H1299 cells (Fig. 5d–f and Fig. S4d–f).

To unravel the underlying mechanism accounting for this discrepancy, our focus shifted to the p53 gene. Notably, H1299 cells are p53-null, while H1975 cells harbor a p53 R273H mutant. Given that p53 has been reported to regulate PD-L1 protein expression [31], we sought to address its potential influence. Thus, we generated a stable H1299 cells which expressed p53 R273H (H1299-p53 R273H) (Fig. 5g). After knocking down PKCα in these cells, we observed a reduction in PD-L1 protein expression, consistent with the results in H1975 cells (Fig. 5h and Fig. S5d). Additionally, the increase in ORAI protein levels seen after PKCα knockdown was abolished in H1299-p53 R273H cells (Fig. S5c). These findings indicate that PKCα regulates PD-L1 expression in a p53-dependent manner, with distinct patterns of PD-L1 modulation observed in the presence and absence of functional p53.

### PKCα regulated STAT1 phosphorylation and nuclear distribution

**Fig. 6:**
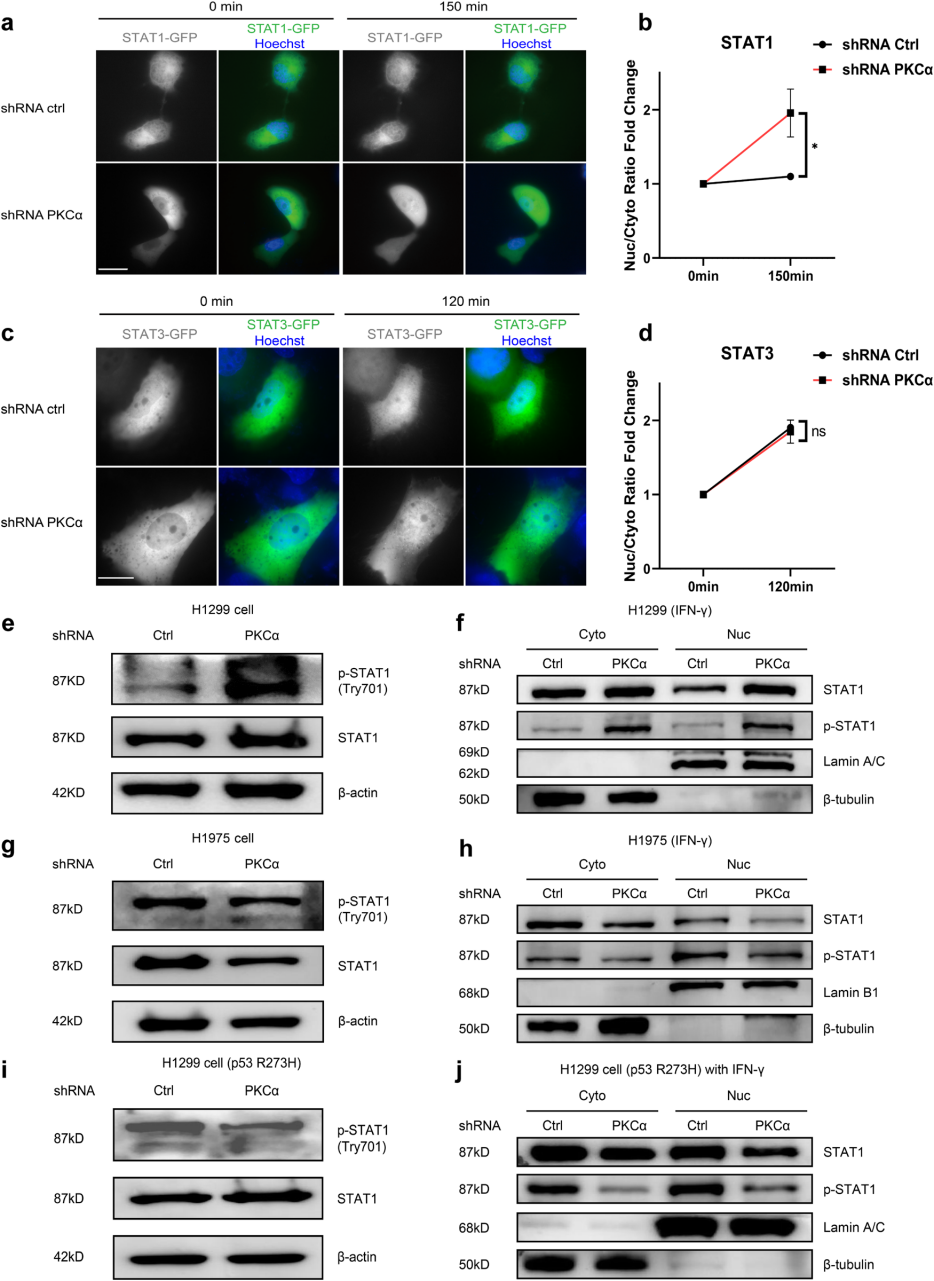
PKCα mediated STAT1 phosphorylation and nuclear distribution. **a** Representative fluorescence microscopy images showing the nuclear translocation of STAT1-GFP in H1299 cells with shRNA control (Ctrl) or shRNA PKCα knockdown over 150 min post IFN-γ (100 ng/ml) treatment. Hoechst 33342 was used to locate the position of cell nucleus. Bar, 50 μm. **b** Quantification of the nuclear/cytoplasmic (Nuc/Cyto) ratio fold change for STAT1-GFP between shRNA Ctrl and shRNA PKCα knockdown cells. Data are presented as means ± SEM. **c** Representative fluorescence microscopy images showing the nuclear translocation of STAT1-GFP in H1299 cells with shRNA Ctrl or shRNA PKCα knockdown over 120 min post IFN-γ (100 ng/ml) treatment. Hoechst 33342 was used to locate the position of cell nucleus. Bar, 20 μm. **d** Quantification of Nuc/Cyto ratio fold change for STAT3-GFP between shRNA Ctrl and shRNA PKCα knockdown cells. Data are presented as means ± SEM. **e** STAT1 and p-STAT1 (Y701) protein levels were detected in whole cell lysates of H1299 cells by immunoblots. **f** Immunoblots analysis on STAT1 and p-STAT1 (Y701) protein expression in the cytoplasm and nucleus of H1299 cells, cells were treated with IFN-γ (1000 UI). β-tubulin was used as a cytoplasmic loading control. Lamin A/C was used as a nuclear loading control. **g** STAT1 and p-STAT1 (Y701) protein levels were detected in whole cell lysates of H1975 cells by immunoblots. **h** Immunoblots analysis on p-STAT1 (Y701) and STAT1 protein expression in the cytoplasm and nucleus of H1975 cells, cells were treated with IFN-γ (1000 UI). β-tubulin was used as a cytoplasmic loading control. Lamin B1 was used as a nuclear loading control. **i** STAT1 and p-STAT1 (Y701) protein levels were detected in H1299 cells overexpressed p53 R273H by immunoblots. **j** Immunoblots analysis on p-STAT1 (Y701) and STAT1 protein expression in the cytoplasm and nucleus of H1299 cells overexpressed p53 R273H, cells were treated with IFN-γ (1000 UI). β-tubulin was used as a cytoplasmic loading control. Lamin A/C was used as a nuclear loading control.

To elucidate the mechanism underlying the regulation of PD-L1 by PKCα, we focused on the transcription factors responsible for PD-L1 expression. The Signal Transducer and Activator of Transcription (STAT) family comprises transcription factors pivotal to many cellular processes. Specifically, within this family, STAT1 and STAT3 were identified as crucial regulators in the control of PD-L1 expression on tumor cells [32]. Thus, we conducted a nuclear translocation assay in H1299 cells overexpressing STAT1 or STAT3. Our results revealed a notably accelerated nuclear translocation of STAT1 induced by IFN-γ with PKCα knock down (Fig. 6a, b), but no such phenomenon was observed with STAT3 (Fig. 6c, d).

Typically, phosphorylated STAT1 (p-STAT) undergoes nuclear translocation in a sequential manner. Upon knocking down PKCα, there was an upregulation in both p-STAT1 and STAT1 expression in H1299 cells (Fig. 6e and Fig. S6a, b). Furthermore, in the presence of IFN-γ stimulation, we also observed a notable increase in both nuclear and cytoplasmic STAT1 and p-STAT1 expression in H1299 cells with PKCα knock down (Fig. 6d and Fig. S6c, d). As expected, PKCα knockdown in H1975 cells led to a significant reduction in both p-STAT1 and STAT1 levels (Fig. 6e and Fig S6e, f) while STAT1 and p-STAT1 protein expression were significant decreased in both cytoplasm and nuclear fractions (Fig. 6h and Fig. S6g, h). Furthermore, in H1299-p53 R273H cells, p-STAT1 levels were significantly reduced, and the STAT1 upregulation observed in H1299 cells was absent (Fig. 6i and Fig. S6i, j). Importantly, the nuclear translocation of both p-STAT1 and STAT1 was also reduced in H1299-p53 R273H cells (Fig. 6j and Fig. S6k, l). Collectively, in the absence of p53, the loss of PKCα resulted in STAT1 phosphorylation and nuclear translocation, potentially contributing to heightened PD-L1 expression. Conversely, in the presence of p53 R273H, PKCα exerted an opposing effect.

### PKCα acts upstream of p53 to regulate STAT1 phosphorylation

**Fig. 7:**
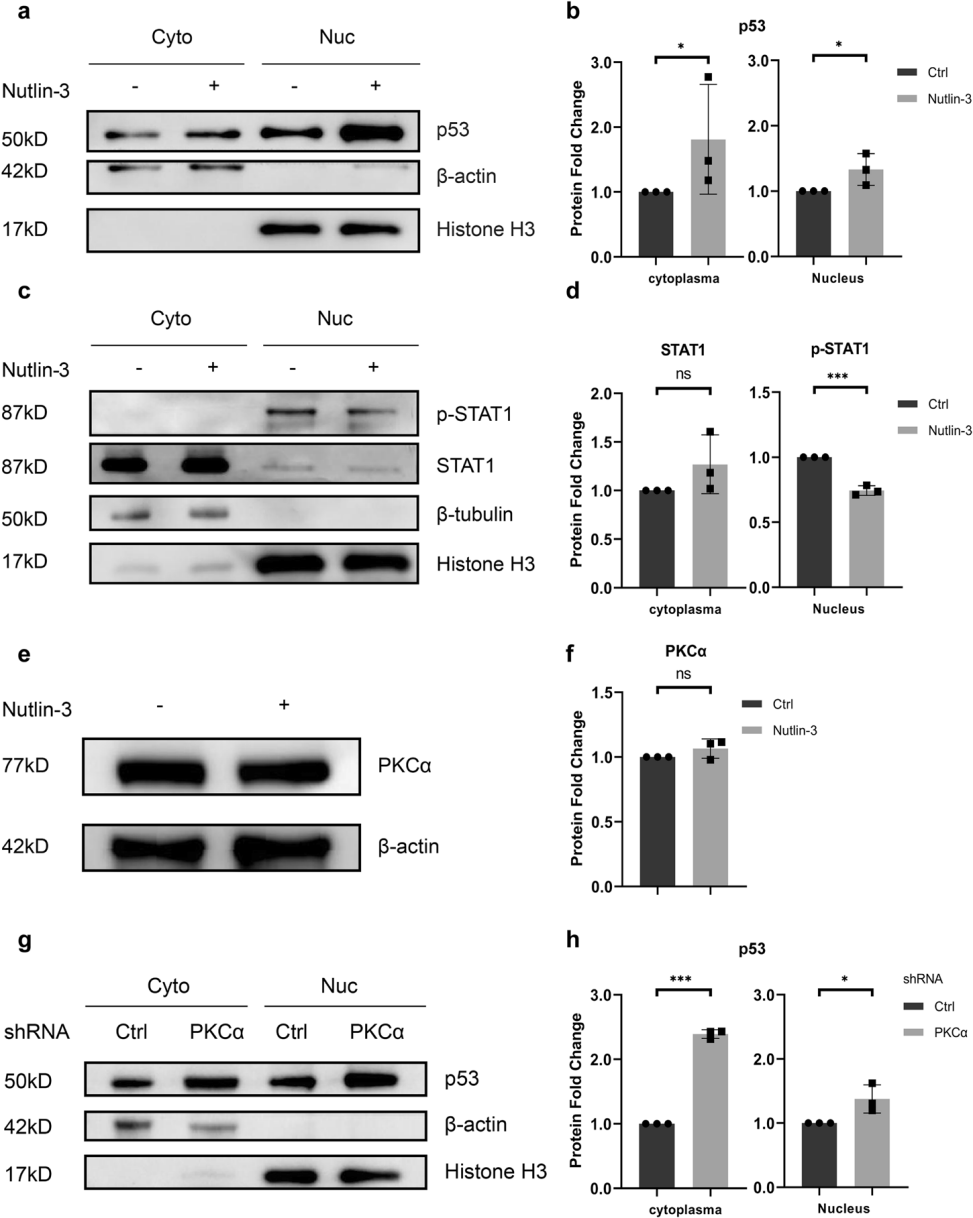
PKCα modulates STAT1 via p53 regulation in H1975 cells. **a** Immunoblot analysis of p53 protein expression in both cytoplasm and nucleus from H1975 treated with or without 15 µM Nutlin-3 for 24 h. β-actin serve as a cytoplasmic loading control, and histone H3 as a nuclear loading control. **b** Quantification of each blot of p53 protein expression normalized to loading control (*n* = 3). **c** Immunoblot analysis of p-STAT1 (Y701) and STAT1 protein expression in both cytoplasm and nucleus from H1975 treated with or without 15 µM Nutlin-3 for 24 h. β-actin serve as a cytoplasmic loading control, and histone H3 as a nuclear loading control. **d** Quantification of each blot of p-STAT1 (Y701) protein expression normalized to nuclear loading control Histone 3 (*n* = 3) and STAT1 protein expression normalized to cytoplasmic loading control β-actin (*n* = 3). **e** Immunoblot analysis on PKCα protein expression after treating with 15 µM Nutlin-3 in H1975 cells for 24 h. **f** Quantification of each blot of PKCα protein expression normalized to β-actin (*n* = 3). **g** Immunoblot analysis of p53 protein expression in the cytoplasm and nucleus of H1975 cells with PKCα knockdown. β-actin serves as a cytoplasmic loading control, and histone H3 as a nuclear loading control. **h** Quantification of each blot of p53 protein expression normalized to corresponding loading control (*n* = 3). Statistic data are presented as means ± SD. *P < 0.05; **P < 0.01; ***P < 0.001 using Student’s *t* test.

In order to explore the function of p53 in PKCα regulating PD-L1 expression, nutlin-3, a p53-MDM2 inhibitor, was used to manipulate p53 expression. After Nutlin-3 treatment, p53 expression increased in both the cytoplasm and nucleus of H1975 cells (Fig. 7a, b). Similar results were observed in H1299-p53 R273H cells (Fig. S7a, b). In H1975 cells, nuclear p-STAT1 levels were reduced following treatment (Fig. 7c, d), and the cytoplasmic STAT1 did not show the significant change (Fig. 7c, e). In H1299-p53 R273H cells, nuclear p-STAT1 was also significantly reduced, consistent with the observations in H1975 cells. Like in H1975 cells, STAT1 expression in the cytoplasm remained unchanged (Fig. S7d). These findings suggest that p53 may inhibit the nuclear translocation of STAT1.

Since both PKCα and p53 are regulators of STAT1 phosphorylation, we aimed at unravelling the relationship between PKCα and p53. The expression of PKCα was unchanged when p53 was upregulated by nutlin-3 in H1975 cells (Fig. 7f, g). However, after knocking down PKCα, the expression of p53 was significantly increased (Fig. 7h, i). We conducted the same experiment in H1299-p53 R273H cells, and the results were consistent with those observed in H1975 cells (Fig. S7e–h). Therefore, we suggest that PKCα is present upstream of p53, and its loss of function causes p53 upregulation, thereby inhibiting STAT1 phosphorylation and reducing total PD-L1 expression.

## Discussion

PD-L1 has been identified as a component of sEVs released contributing to tumor progression by inducing systemic immunosuppression [33]. Nonetheless, most of the evidence on the role of PD-L1 sEVs was generated at the protein and biochemical level of analyses [34, 35]. For a comprehensive understanding of the phenomenon, the acquisition of real-time data is imperative for delving into the dynamics of sEVs-PD-L1 biogenesis and release. Previous work has demonstrated colocalization of PD-L1 with the HRS subunit of the ESCRT upon stimulation with IFN-γ. Additionally, the co-localization of PD-L1 with CD63 was observed under the same IFN-γ stimulation [36]. Building on this, we co-expressed PD-L1 with candidate markers for sEVs, endosomes, lysosomes and autophagosomes to scrutinize their colocalization patterns. As anticipated, PD-L1 showed the highest colocalization score with the sEVs markers, such as CD63 and CD81. Moreover, a substantial colocalization score was obtained between PD-L1 and markers of endosomes (Rab5, Rab7) and lysosomes (Lamp1). This observation can be attributed to their roles in cargo trafficking and degradation processes of sEVs [37, 38].

The study of the dynamics of sEVs release has posed a long-standing challenge, yet it holds paramount promise for understanding in vivo physiological processes [39]. A previously study developed a pH sensitive reporter for monitoring the sEVs release of CD63, enabling real-time visualization. This work suggested that signal duration of MVB-PM fusion was influenced by the heterogeneous composition and size of MVBs [22]. In our study, we adopted a similar approach to explore the dynamics and characteristics of PD-L1 sEVs release. While we observed that the dynamics of PD-L1 sEVs release overlapped with those of other sEVs markers, differences in the duration of MVB-PM fusion events were apparent. This discrepancy between PD-L1 sEVs and other sEVs reflected the variability in the composition of MVBs, as evidenced by incomplete co-localization between PD-L1 and other sEVs markers.

It has been well-documented that various biological processes involving sEVs exhibit calcium dependence. Enhancing calcium influx has been shown to significantly boost sEVs production through the ESCRT pathway [8]. Both calcium influx and calcium-triggered depolarization have been linked to sEVs secretion [40–42]. In our study, ATP stimulation promoted sEVs secretion for both PD-L1 and CD63. After chelating calcium with BAPTA-AM and EDTA, the early ATP-induced increase of the PD-L1 sEVs fusion event rate was reduced, which is different from the results of a previous study using histamine stimulation [22]. A likely cause for the discrepant results could be the different types of G protein-coupled receptors (GPCRs) which are activated either by histamine or by ATP [43, 44]. Additionally, differences in cell types and sEVs components may contribute to the different findings.

The conventional PKCs (PKCα, PKCβ, PKCγ) are recognized as calcium-dependent members within the broader PKC family. Notably, PKCα demonstrates ubiquitous expression and becomes activated in response to diverse physiological stimuli. Its association with cancer progression is underscored by its regulatory impact on cell growth, migration, and anti-apoptotic activities [45]. In terms of the regulation of PD-L1 expression, PKCα has been reported to exert variable effects in different cell types. Depleting PKCα through RNA interference in prostate cancer cells resulted in a reduction of PD-L1 expression [46]. In another study on lung cancer cells, it was found that knock down of PKCα inhibits the downregulation of PD-L1 induced by GSK3β dephosphorylation [47]. In our investigation, we inhibited PKCα activity by using the PKC inhibitor GÖ6983 and by shRNA PKCα and found that the ATP-induced fusion process of PD-L1 sEVs was reduced. Besides, we found that the rate of fusion events of PD-L1 sEVs under resting conditions was increased, in line with PD-L1 expression in sEVs. Thus, our findings indicated that PKCα has complex effects on PD-L1 sEVs secretion.

In the regulation of PD-L1 in sEVs by PKCα, p53 plays a crucial role. In H1299 cells with p53 deficiency, the knockdown of PKCα resulted in a significant increase in PD-L1 expression in sEVs, accompanied by a concurrent upregulation of other sEVs markers. We also observed an elevated expression of ORAI1 in whole cell lysates, indicating an enhancement of intracellular calcium signaling. This enhancement in calcium signaling aligns with previous reports linking increased calcium levels to augmented sEVs secretion [40]. Recent research indicates that the SNARE complex may play a pivotal role in the MVB-PM fusion of CD63-positive vesicles [48]. However, the same key SNARE proteins do not appear to be responsible for the increased sEV-PD-L1 secretion induced by PKCα knockdown. Previous studies have demonstrated that Munc18-2 deletion in an allergic asthma model inhibits ATP-mediated mucin secretion, reducing airway mucus blockage and emphysema [27]. Given the similarity in airway mucin secretion and our findings, we propose that PKCα knockdown leads to Munc18-2 downregulation, which disrupts ATP-induced fusion events. Munc18-3, localized at the apical plasma membrane where exocytosis occurs, interacts with syntaxin4. Takuma et al. [28] suggested that Munc18-3 plays a role in targeting syntaxin4 in HSY human parotid epithelial cells to promote MVB-PM fusion and vesicular secretion, implying that the enhanced PD-L1 sEVs fusion events at resting state in PKCα knockdown H1299 cells may rely on the upregulated Munc18-3. Notably, the expression of PD-L1 in whole cell lysates also exhibited an elevation, suggesting that PKCα knock down not only promoted the sEVs secretion, but also increased the total PD-L1 amount. Furthermore, aiming at mechanistic insights into the increased PD-L1 expression, the STAT family is often explored as a potential cause for enhanced PD-L1 transcription [49]. In our experiments, we observed an accelerated nuclear translocation of STAT1, accompanied by increased total expression, phosphorylation and nuclear localization following PKCα knockdown. Therefore, we propose that the augmented PD-L1 levels resulting from PKCα knock down may be attributed to an increased phosphorylation and nuclear translocation of STAT1 in H1299 cells. However, in both H1975 cells harboring p53 R273H mutation and H1299 cells exogenously expressing p53 R273H, we obtained results contrary to those in p53-deficient cells. We observed a reduction of PD-L1 expression in both sEVs and whole cell lysates, accompanied by a decreased STAT1 phosphorylation and nuclear translocation. These findings suggest that the PKCα-mediated regulation of PD-L1 is dependent on p53. Currently, there is a controversy surrounding the role of PKCα in lung cancer [50–54]. In cells expressing the p53 WT gene, the activation of PKCα is often associated with tumor suppression. Conversely, in cases where p53 undergoes mutations, inhibiting PKCα typically imposes restrictions on the growth of tumor cells. Thus, we speculate that p53 is likely the possible explanation for this controversy because the frequently mutated p53 protein could exert variable oncogenic functions.

In elucidating the intricate interdependence of p53 and PKCα, blockade of PKCα activity or silencing PKCα gene has been associated with the activation of p53 and the upregulation of the p53 gene expression [55, 56]. A study by Smith et al. [57] revealed that integrin αv promotes the expression of PKCα, thereby suppressing apoptosis in melanoma cells. The underlying mechanism may involve PKCα facilitating the nuclear export of p53 or impeding the translocation of p53 from the cytoplasm to the nucleus. Nevertheless, investigations into the role of PKCα remain limited, particularly concerning its role in lung cancer cells. Our findings indicate that the expression of PKCα remained unaltered following the upregulation of p53 using nutlin-3. However, upon the depletion of PKCα, we observed an augmentation in p53 expression in both the nucleus and cytoplasm. Consistent with prior research, we found that loss of PKCα upregulates p53, indicating that PKCα functions as an upstream regulator of p53, exerting an influence over p53 expression and nuclear translocation.

**Fig. 8:**
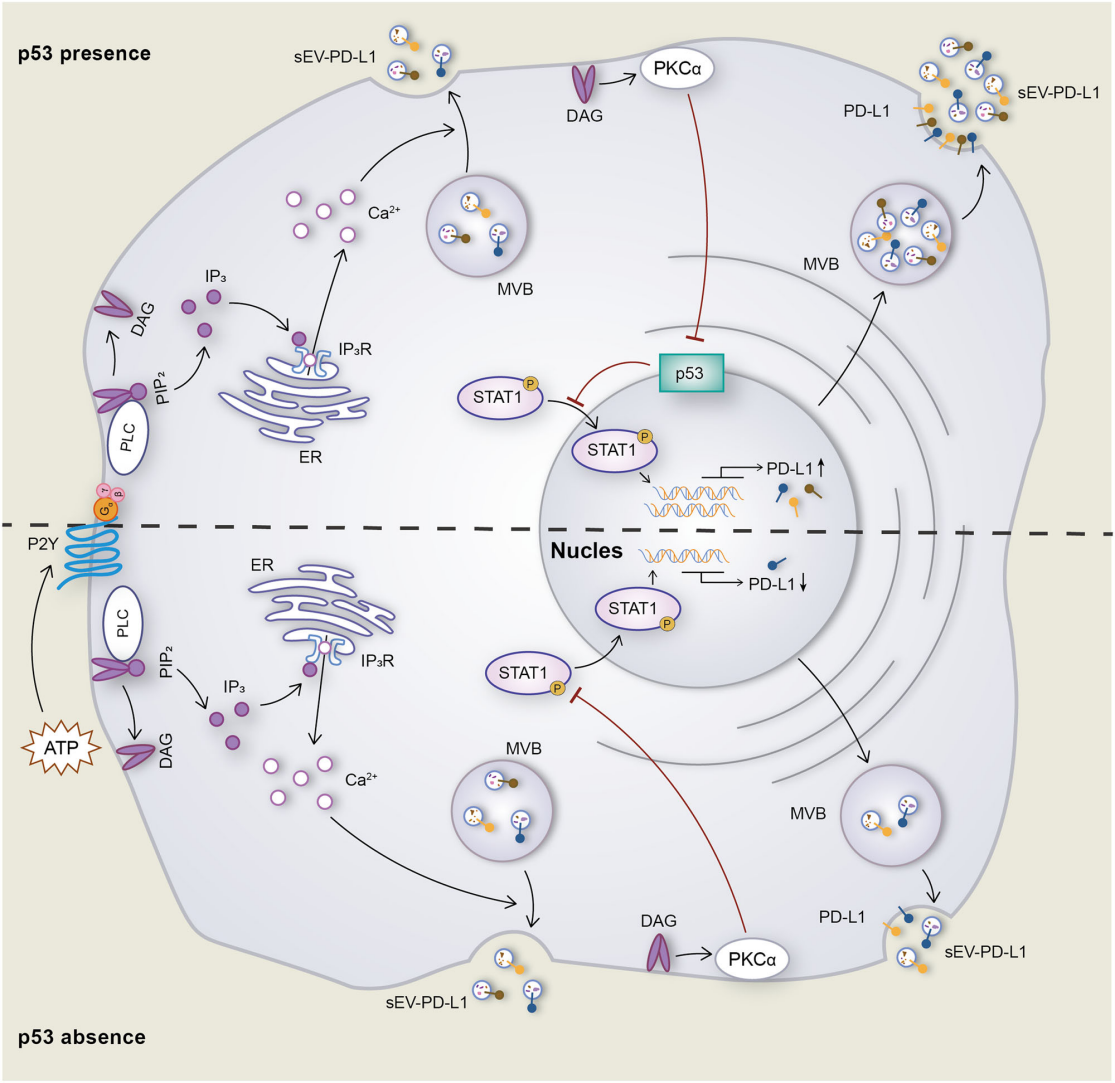
Schematic representation of PKCα regulation of PD-L1 secretion in NSCLC cells. In p53 absent situation, PKCα inhibited STAT1 phosphorylation and nuclear translocation which led to a reduction of PD-L1 transcription, ultimately leading to a decrease in PD-L1 secretion. In contrast, PKCα inhibited nuclear translocation of p53, leading to increased STAT1 phosphorylation and nuclear translocation which led to increasing of PD-L1 transcription and PD-L1 secretion.

In summary, our study demonstrates that PD-L1 exhibits a high degree of co-localization and shares remarkably similar secretion kinetics with CD63, as observed in real-time experiments. This highlights PD-L1’s potential utility as a novel sEVs marker for future investigations. Our findings also emphasize the pivotal role of calcium and PKCα for the secretion of sEVs-PD-L1 in NSCLC cells. In line with the important role of p53 in tumorigenesis and its mutations being closely associated with the prognosis of cancer patients, we found that PKCα-regulated PD-L1 expression is dependent on p53 (Fig. 8). Our findings provide innovative insights into potential strategies for immune escape therapy in lung cancer targeting PD-L1. We conclude that it is imperative to consider the role of p53, PKCα and the associated signaling network in future studies.

## Materials and methods

### Cell culture

H1299 (NCI-H1299) and H1975 (NCI-H1975) cells were acquired from the American Typical Culture Collection (ATCC, Manassas, VA, USA). H1299 and H1975 cells were cultured in PRMI-1640 Medium (31800022, Thermo Fisher Scientific, Waltham, MA, USA) supplemented with 10% fetal bovine serum and 1% penicillin/streptomycin.293 T cells were cultured in DMEM medium (12800017, Thermo Fisher Scientific, Waltham, MA, USA) supplemented with 10% fetal bovine serum (10270106, Thermo Fisher Scientific, Waltham, MA, USA) and 1% penicillin/streptomycin (A1113802, Thermo Fisher Scientific, Waltham, MA, USA). The incubator was set at 37 °C with an environment of 5% CO_2_.

### Plasmids

The pLJM1-PD-L1-pHluorin plasmid was generated as our previous study described [15]. The pCMV-CD63-pHluorin was a gift from Tom Martin (University of Wisconsin-Madison). The pHR-CLIP-PD-L1 was a gift from Zhao (Enfu Hui’s lab in UCSD). The pLJM1_FLAG was a gift from Zhang (Central South University).

pCMV-Sport6-CD9-pHluorin (# 130905), pCMV-Sport6-CD81-pHluorin (# 130903), mCherry-CD9-10 (# 55013), mCherry-CD81-10 (# 55012) pKT2-CAGXSP-CD63mScarlet (# 182972), Lamp1-mScarlet-I (# 98827), iRFP-FRB-Rab5 (# 51612), iRFP-FRB-Rab7 (# 51613), mCherry-hLC3B-pcDNA3.1 (# 40827), eGFP-STAT1-WT (# 12301), pEGFP-N1-STAT3 (# 111934) and pLenti6/V5-p53_R273H (# 22934) were purchase from Addgene Plasmid (MA, USA).

### shRNA sequences

shRNA sequences were constructed and packaged by VectorBuilder (Yunzhou Bio, China). pLV[shRNA]-hPRKCA (ACCATCCGCTCCACACTAAAT) and pLV[shRNA]-Scamblem (GCTTCGCGCCGTAGTCTTA). Lenti-viral packaging plasmids were showed as follow: pMDLg/pRRE, pRSV-Rev and pMD2.G (# 12251, # 12253, # 12259) were purchase from Addgene Plasmid (MA, USA).

### Transient transfection

Plasmid transfections were performed using UltraFection 3.0 reagent (Beijing 4A Biotech, China) typically on a 6-well scale with a 1 µg:3 µl ratio of DNA to Ultra Fection reagent according to the manufacturer’s instruction. Cells were transfected with plasmid at 50–70% confluency. After 6 h of transfection, fresh medium exchange is required. Cells would be examined after 24 h transfection.

### Stable cell line generation

293 T cells were used to generate the lentiviral lentivirus-containing medium for stable cell lines. 293 T cells were grown until fusion reached 60-70% prior to co-transfected using pLJM1_pHluorin-PD-L1, pLV[shRNA]-Neo-U6>hPRKCA, pLV[shRNA]-Scamblem and pLenti6/V5-p53_R273H (# 22934) with the lentiviral packaging plasmids. After 6-h transfection fresh medium exchange was needed. Followed by 48 and 72 h of incubation, lentivirus-containing supernatant were collected for centrifuging at 1000*g* to eliminate cell debris, and filtered through a 0.45 μm filter. After wild type cells grown to 50–60% confluency in 6-well plates, we replaced the medium with 2 ml of medium containing lentivirus and 10 ug/mL polybrene. Fresh medium needed to exchange in the next day. After 48 h of infection, cells were treated with 1 μg/mL puromycin, 1000 μg/mL G418, or 10 μg/mL blasticidin to select for stable cell lines. Purification duration usually last for 2 weeks, then stable cells lines were identified by fluorescence microscopy (Olympus ORCA-Flash 40 LT Plus Scientific CMOS Digital Camera), RT-qPCR and Western blot analysis.

### RT-PCR

Total RNA was isolated from whole cell with TRIzol reagent (Invitrogen, California, USA) as described in the manufactures’ protocol. RNA concentration was determined by Nano Drop 2000c spectrophotometer (Thermo Science, California, USA). Total RNA was converted to complementary DNA (cDNA) using Transcriptor Universal cDNA Master. cDNA samples were mixed with SYBR^TM^ Green Master Mix in a total volume of 20 μl and run on ViiA^TM^7 Real Time PCR System (Applied Biosystems). Amplification and melting curves were analyzed using the LightCycler480 Software release 1.5.1.

The primers used for PKCα (PRKCA) were as follow: Forward primer, 5′- GCCTATGGCGTCCTGTTGTATG-3′, and reverse primer 5′- GAAACAGCCTCCTTGGACAAGG-3′.

### sEVs isolation

Small extracellular vesicles (sEVs) were isolated from the supernatant of 48 h cultured cells after transient transfection and stable cell line by the following steps. We seed 5 × 10^6^ cells per 175 flask and incubated for 24 h. Next cells were washed 3 times with phosphate-buffered saline (PBS) and then cultured in RPMI-1640 Medium containing 10% sEVs-depleted Fetal Bovine Serum. We collected supernatant of the 48 h of incubated cells, the supernatant of two flasks were combined. Cells medium supernatant was subjected to a centrifugation protocol. First centrifugation at 300*g* for 10 min at room temperature, followed by 2000*g* for 20 min at 4 °C, then 10,000*g* for 60 min at 4 °C. Next the supernatant was centrifuged at 100k g for 70 min at 4 °C and precipitate was washed with PBS, centrifuged again at 100k g for 70 min at 4 °C. Centrifugal products were resuspended in PBS for image acquisition and RIPA buffer for protein immunoblot analysis. To ensure consistency in both Nanoparticle Tracking Analysis and Western Blot analysis, all comparative sEV samples were collected from an equal number of cells.

### sEVs immunolabeling

sEV samples were incubated with CD63 antibody (MX-49.129.5, Santa Cruz, sc5275) at a dilution of 1:50 in a total volume of 100 μl at 4 °C overnight. Following incubation, the samples underwent ultracentrifugation at 100,000×*g* for 2 h to remove unbound antibodies. The precipitate was then incubated with a 1:1000 dilution of goat anti-mouse IgG H&L secondary antibody conjugated to Alexa Fluor® 405 (ab175660) in a total volume of 100 μl at 4 °C for 2 h. To further purify the labeled sEVs, a second round of ultracentrifugation at 100,000×*g* for 2 h was performed, followed by supernatant removal. The final precipitate was resuspended in PBS for nanoparticle tracking analysis (NTA).

### Nanoparticle tracking analysis (NTA)

The size distribution and particle concentration of isolated sEV samples were analyzed using the NanoSight NS300 (Malvern Instruments, Ltd., UK). Prior to analysis, the samples were diluted 10- to 100-fold in PBS to ensure that fewer than 200 particles were visible within the field of view. The detection of sEVs was proportional to the number of sEV-secreting cells. For consistency, all comparative sEV samples were collected from an identical number of cells and diluted using the same dilution factor. Each sample was measured more than three times, and data were processed with the NanoSight NS300 NTA software (version 3.0, Malvern Instruments, Ltd., UK).

### Protein immunoblot analysis

Cells and sEVs samples were generated as described in previous study [15]. Nuclear and cytoplasmic protein were isolated using the Nuclear and Cytoplasmic Protein Extraction Kit (Beyotime, P0027), following the manufacturer’s instructions meticulously. Protein samples were electrophoresed on 10% SDS-PAGE gels and transferred to Nitrocellulose Transfer Membrane. 5% lipid milk prepared by Tris-buffered saline solution (TBS) containing 0.1% Tween 20 was used for 1-h membrane blocking at room temperature, and the membranes were incubated with primary antibodies overnight at 4 °C in shaker. After washing 3 times for 5 min each time with TBS, the membranes were incubated with secondary antibodies for 2 h at room temperature. Protein bands images were acquired by ECL reagents and Amersham Imager 800 (GE Healthcare, CA, USA), software ImageJ (NIH, Bethesda, USA) was used for images processing and quantified.

### Antibodies and reagents

The anti-PKCα (Rabbit, Ab32376) and ORAI1 (Mouse, Ab111960) monoclonal antibodies from Abcam (Cambridge, England) were used at a 1:2000 dilution. Monoclonal antibodies including CD9 (Rabbit, CST#13403), ALIX (Mouse, CST#2171), PD-L1 (Rabbit, CST#13684), β-actin (Mouse, CST 3700S), and β-tubulin (Rabbit, CST#2146) were obtained from Cell Signaling Technology (MA, USA) and used at a 1:1000 dilution. Monoclonal antibodies CD63 (Mouse, sc5275), Lamin A/C (Mouse, sc7292), SANP23(Mouse, sc166244), Synataxin4(Mouse, sc14455), VAMP7(Mouse, sc166394), Munc18-2 (Mouse, sc-390503), Munc18-3 (Mousesc-373813), p53 (Mouse, sc-126), and GAPDH (Mouse, sc-47724) were obtained from Santa Cruz (TX, USA) were used at a 1:1000 dilution. Monoclonal antibodies Munc18-1 (Rabbit, HA722250), p-PKCα T638 (Rabbit, ET1702-17), STAT1 (Rabbit, ET1606-39), p-STAT1 Y701 (Rabbit, HA722083), Lamin B1 (Rabbit, ET1606-27) from HuaBio (Hangzhou, China) was used at a 1:1000 dilution. Monoclonal antibodies Histone H3 (Mouse, EM30605) from HuaBio was used at a 1:5000 dilution.

For secondary antibody were Goat Anti-mouse IgG-HRP (abmart, #M21001), Goat Anti-rabbit IgG-HRP (abmart, #M21002) used as 1:3000. Goat Anti-Mouse IgG H&L Alexa Fluor® 405 (ab175660) were used as 1:1000.

PMA/TPA (Beyotime, S819), human interferon-gamma (IFN-γ) (Sigma-Aldrich, I17001), and Nutlin-3 (Absin, abs813389) were prepared in culture medium for use in subsequent experiments. Adenosine 5′-triphosphate magnesium salt (Mg-ATP) (Sigma-Aldrich, A9187) was prepared in Ringer buffer with a final concentration of 100 µM. BAPTA-AM (Sigma-Aldrich, A1076) was prepared in medium with a final concentration of 20 µM and incubated for 20 min at 37 °C. EGTA (Sigma-Aldrich, E4378) was prepared at 2 mM in medium or Mg-ATP solution for incubated and drug stimulation. GÖ 6983 (LC laboratories, G-7700) were applied at a final concentration of 1 µM for 2 h at 37 °C.

### Image acquisition and data analysis

#### LBS Light-sheet microscope

Ø 5-mm coverslips were soaked in 75% ethanol for 3–5 h and irradiated with UV light for 30 min before use. Transfected cells were seeded on coverslips at a density of 10^4^–10^5^. Coverslips would be handled in a specimen holder for imaging acquiring. 15 ml ringer buffer (2 mM CaCl_2_, 2.5 mM KCl, 145 mM NaCl, 1 mM MgCl_2_, 10 mM glucose, 10 mM Hepes, pH 7.4) or PBS solution were prepared as buffer solution during light-sheet microscopy. All images were acquired on Litone LBS high resolution lightsheet microscope (Light Innovation Technology Ltd.) equipped with 25×1.1NA widefield objective (N25X-APO-MP) which is suitable for both water dipping and water immersion setups. LitScan 2.0 software was used for controlling and acquiring data. Image J (NIS, USA) software was used for images optimization and Pearson’s correlation coefficients calculating.

### Live cell fluorescence microscopy assay

Coverslips were coated with poly-l-lysine for 1 h, using deionized water wash and dry. After 24-h transfection, cells were cultured in petri dish with several coverslips overnight. Coverslips containing cells were placed in an imaging chamber and perfused with Ringer buffer (2 mM CaCl_2_, 2.5 mM KCl, 145 mM NaCl, 1 mM MgCl_2_, 10 mM glucose, 10 mM Hepes, pH 7.4). All images and videos were taken by API Delta Vision Live-cell Imaging System (GE Healthcare Company, CA, USA) using 60× or 100× flat-field apochromatic mirrors. The time interval for each video was 1 s or 2 s with a 0.25 s exposure time. All videos were acquired with sofwWoRx 7.0 software.

ATP-stimulation protocol was performed with 6 min imaging in total, including 3 min superfusion with 100 µM Mg-ATP in Ringer buffer. Intracellular pH was neutralized with normal Ringer buffer containing 50 mM NH_4_Cl instead of NaCl.

For the nuclear translocation assay, coverslips with cells were positioned in an imaging chamber containing a small volume of complete culture medium. Subsequently, superfusion with 100 ng/ml IFN-γ (diluted in complete culture medium), images were captured both before administration and 150 min after administration. All imaging experiments were performed at RT (21–24 °C).

### Statistical analysis

Data statistical analysis was performed using GraphPad Prism version 8.0. Two groups’ data analysis was used Student’s *t* test for significance. One-way ANOVA was used for three or more groups’ data analysis. Pearson correlation was used for co-location analysis. The *p*-value less than 0.05 were the threshold for us to consider significance different.

## Supplementary information

Video 1

Video 2

Video 3

Video 4

Video 5

Video 6

Video 7

Video 8

Supplement Figure and legend Cell Death X Disease

Original Data

## Data Availability

All primary data presented in this study are available upon reasonable request to the corresponding authors.

## References

[1] Zaborowski MP, Balaj L, Breakefield XO, Lai CP. Extracellular vesicles: composition, biological relevance, and methods of study. Bioscience. 2015;65:783–97. 26955082 10.1093/biosci/biv084 PMC4776721

[2] Lim HJ, Yoon H, Kim H, Kang YW, Kim JE, Kim OY, et al. Extracellular vesicle proteomes shed light on the evolutionary, interactive, and functional divergence of their biogenesis mechanisms. Front Cell Dev Biol. 2021;9:734950. 34660591 10.3389/fcell.2021.734950 PMC8517337

[3] Xu M, Ji J, Jin D, Wu Y, Wu T, Lin R, et al. The biogenesis and secretion of exosomes and multivesicular bodies (MVBs): intercellular shuttles and implications in human diseases. Genes Dis. 2023;10:1894–907. 37492712 10.1016/j.gendis.2022.03.021 PMC10363595

[4] Dong H, Strome SE, Salomao DR, Tamura H, Hirano F, Flies DB, et al. Tumor-associated B7-H1 promotes T-cell apoptosis: a potential mechanism of immune evasion. Nat Med. 2002;8:793–800. 12091876 10.1038/nm730

[5] Sanchez-Alvarez M, Del Pozo MA. An unexpected role for PD-L1 in front-rear polarization and directional migration. J Cell Biol. 2022;221:e202203137. 35416931 10.1083/jcb.202203137 PMC9011200

[6] Yu ZL, Liu JY, Chen G. Small extracellular vesicle PD-L1 in cancer: the knowns and unknowns. NPJ Precis Oncol. 2022;6:42. 35729210 10.1038/s41698-022-00287-3 PMC9213536

[7] Liu J, Peng X, Yang S, Li X, Huang M, Wei S, et al. Extracellular vesicle PD-L1 in reshaping tumor immune microenvironment: biological function and potential therapy strategies. Cell Commun Signal. 2022;20:14. 35090497 10.1186/s12964-021-00816-w PMC8796536

[8] Ambattu LA, Ramesan S, Dekiwadia C, Hanssen E, Li H, Yeo LY. High frequency acoustic cell stimulation promotes exosome generation regulated by a calcium-dependent mechanism. Commun Biol. 2020;3:553. 33020585 10.1038/s42003-020-01277-6 PMC7536404

[9] Burtenshaw D, Regan B, Owen K, Collins D, McEneaney D, Megson IL, et al. Exosomal composition, biogenesis and profiling using point-of-care diagnostics-implications for cardiovascular disease. Front Cell Dev Biol. 2022;10:853451. 35721503 10.3389/fcell.2022.853451 PMC9198276

[10] Scarpellino G, Munaron L, Cantelmo AR, Fiorio Pla A. Calcium-permeable channels in tumor vascularization: peculiar sensors of microenvironmental chemical and physical cues. Rev Physiol Biochem Pharmacol. 2022;182:111–37. 32809072 10.1007/112_2020_32

[11] Romito O, Gueguinou M, Raoul W, Champion O, Robert A, Trebak M, et al. Calcium signaling: a therapeutic target to overcome resistance to therapies in cancer. Cell Calcium. 2022;108:102673. 36410063 10.1016/j.ceca.2022.102673

[12] Pan X, Li R, Guo H, Zhang W, Xu X, Chen X, et al. Dihydropyridine calcium channel blockers suppress the transcription of PD-L1 by inhibiting the activation of STAT1. Front Pharmacol. 2020;11:539261. 33519429 10.3389/fphar.2020.539261 PMC7838064

[13] Alam MR, Rahman MM, Li Z. The link between intracellular calcium signaling and exosomal PD-L1 in cancer progression and immunotherapy. Genes Dis. 2024;11:321–34. 37588227 10.1016/j.gendis.2023.01.026 PMC10425812

[14] Khan FM, Saleh E, Alawadhi H, Harati R, Zimmermann WH, El-Awady R. Inhibition of exosome release by ketotifen enhances sensitivity of cancer cells to doxorubicin. Cancer Biol Ther. 2018;19:25–33. 29244610 10.1080/15384047.2017.1394544 PMC5790333

[15] Chen X, Li J, Zhang R, Zhang Y, Wang X, Leung EL, et al. Suppression of PD-L1 release from small extracellular vesicles promotes systemic anti-tumor immunity by targeting ORAI1 calcium channels. J Extracell Vesicles. 2022;11:e12279. 36482876 10.1002/jev2.12279 PMC9732629

[16] Newton AC. Protein kinase C: poised to signal. Am J Physiol Endocrinol Metab. 2010;298:E395–402. 19934406 10.1152/ajpendo.00477.2009 PMC2838521

[17] Lim PS, Sutton CR, Rao S. Protein kinase C in the immune system: from signalling to chromatin regulation. Immunology. 2015;146:508–22. 26194700 10.1111/imm.12510 PMC4693901

[18] Singh RK, Kumar S, Gautam PK, Tomar MS, Verma PK, Singh SP, et al. Protein kinase C-alpha and the regulation of diverse cell responses. Biomol Concepts. 2017;8:143–53. 28841566 10.1515/bmc-2017-0005

[19] Lum MA, Pundt KE, Paluch BE, Black AR, Black JD. Agonist-induced down-regulation of endogenous protein kinase c alpha through an endolysosomal mechanism. J Biol Chem. 2013;288:13093–109. 23508961 10.1074/jbc.M112.437061 PMC3642351

[20] Fioravante D, Chu Y, Myoga MH, Leitges M, Regehr WG. Calcium-dependent isoforms of protein kinase C mediate posttetanic potentiation at the calyx of Held. Neuron. 2011;70:1005–19. 21658591 10.1016/j.neuron.2011.04.019 PMC3113702

[21] Kowal J, Arras G, Colombo M, Jouve M, Morath JP, Primdal-Bengtson B, et al. Proteomic comparison defines novel markers to characterize heterogeneous populations of extracellular vesicle subtypes. Proc Natl Acad Sci USA. 2016;113:E968–77. 26858453 10.1073/pnas.1521230113 PMC4776515

[22] Verweij FJ, Bebelman MP, Jimenez CR, Garcia-Vallejo JJ, Janssen H, Neefjes J, et al. Quantifying exosome secretion from single cells reveals a modulatory role for GPCR signaling. J Cell Biol. 2018;217:1129–42. 29339438 10.1083/jcb.201703206 PMC5839777

[23] Edgar JR, Manna PT, Nishimura S, Banting G, Robinson MS. Tetherin is an exosomal tether. Elife. 2016;5:e17180. 27657169 10.7554/eLife.17180 PMC5033606

[24] Vultaggio-Poma V, Sarti AC, Di Virgilio F. Extracellular ATP: a feasible target for cancer therapy. Cells. 2020;9:2496. 33212982 10.3390/cells9112496 PMC7698494

[25] Martinez-Martinez E, Sanchez-Vazquez VH, Leon-Aparicio D, Sanchez-Collado J, Gallegos-Gomez ML, Rosado JA, et al. PKC-mediated orai1 channel phosphorylation modulates Ca(2+) signaling in HeLa cells. Cells. 2022;11:2037. 35805121 10.3390/cells11132037 PMC9266177

[26] Black AR, Black JD. The complexities of PKCalpha signaling in cancer. Adv Biol Regul. 2021;80:100769. 33307285 10.1016/j.jbior.2020.100769 PMC8141086

[27] Jaramillo AM, Piccotti L, Velasco WV, Delgado ASH, Azzegagh Z, Chung F, et al. Different Munc18 proteins mediate baseline and stimulated airway mucin secretion. JCI Insight. 2019;4:e124815. 30721150 10.1172/jci.insight.124815 PMC6483006

[28] Takuma T, Arakawa T, Okayama M, Mizoguchi I, Tanimura A, Tajima Y. Trafficking of green fluorescent protein-tagged SNARE proteins in HSY cells. J Biochem. 2002;132:729–35. 12417022 10.1093/oxfordjournals.jbchem.a003280

[29] Hermoso M, Olivero P, Torres R, Riveros A, Quest AF, Stutzin A. Cell volume regulation in response to hypotonicity is impaired in HeLa cells expressing a protein kinase Calpha mutant lacking kinase activity. J Biol Chem. 2004;279:17681–9. 14960580 10.1074/jbc.M304506200

[30] Srivastava J, Procyk KJ, Iturrioz X, Parker PJ. Phosphorylation is required for PMA- and cell-cycle-induced degradation of protein kinase Cdelta. Biochem J. 2002;368:349–55. 12207561 10.1042/BJ20020737 PMC1222988

[31] Cortez MA, Ivan C, Valdecanas D, Wang X, Peltier HJ, Ye Y, et al. PDL1 regulation by p53 via miR-34. J Natl Cancer Inst. 2016;108:303. 10.1093/jnci/djv303 PMC4862407 26577528

[32] Doi T, Ishikawa T, Okayama T, Oka K, Mizushima K, Yasuda T, et al. The JAK/STAT pathway is involved in the upregulation of PD-L1 expression in pancreatic cancer cell lines. Oncol Rep. 2017;37:1545–54. 28112370 10.3892/or.2017.5399

[33] Poggio M, Hu T, Pai CC, Chu B, Belair CD, Chang A, et al. Suppression of exosomal PD-L1 induces systemic anti-tumor immunity and memory. Cell. 2019;177:414–27 e13. 30951669 10.1016/j.cell.2019.02.016 PMC6499401

[34] Lamberti G, Sisi M, Andrini E, Palladini A, Giunchi F, Lollini PL, et al. The mechanisms of PD-L1 regulation in non-small-cell lung cancer (NSCLC): which are the involved players? Cancers (Basel). 2020;12:3129. 33114576 10.3390/cancers12113129 PMC7692442

[35] Tang Y, Zhang P, Wang Y, Wang J, Su M, Wang Y, et al. The biogenesis, biology, and clinical significance of exosomal PD-L1 in cancer. Front Immunol. 2020;11:604. 32322256 10.3389/fimmu.2020.00604 PMC7158891

[36] Chen G, Huang AC, Zhang W, Zhang G, Wu M, Xu W, et al. Exosomal PD-L1 contributes to immunosuppression and is associated with anti-PD-1 response. Nature. 2018;560:382–6. 30089911 10.1038/s41586-018-0392-8 PMC6095740

[37] Cheerathodi M, Nkosi D, Cone AS, York SB, Meckes DG Jr. Epstein-Barr virus LMP1 modulates the CD63 interactome. Viruses. 2018;13(4), 675. 10.3390/v13040675 PMC8071190 33920772

[38] Zou Y, Wang H, Shapiro JL, Okamoto CT, Brookes SJ, Lyngstadaas SP, et al. Determination of protein regions responsible for interactions of amelogenin with CD63 and LAMP1. Biochem J. 2007;408:347–54. 17708745 10.1042/BJ20070881 PMC2267358

[39] Kalluri R, LeBleu VS. The biology, function, and biomedical applications of exosomes. Science. 2020;367:eaau6977. 32029601 10.1126/science.aau6977 PMC7717626

[40] Messenger SW, Woo SS, Sun Z, Martin TFJ. A Ca(2+)-stimulated exosome release pathway in cancer cells is regulated by Munc13-4. J Cell Biol. 2018;217:2877–90. 29930202 10.1083/jcb.201710132 PMC6080937

[41] Sako Y, Sato-Kaneko F, Shukla NM, Yao S, Belsuzarri MM, Chan M, et al. Identification of a novel small molecule that enhances the release of extracellular vesicles with immunostimulatory potency via induction of calcium influx. ACS Chem Biol. 2023;18:982–93. 37039433 10.1021/acschembio.3c00134 PMC10127211

[42] Shaheen H, Singh S, Melnik R. A neuron-glial model of exosomal release in the onset and progression of Alzheimer’s disease. Front Comput Neurosci. 2021;15:653097. 34616283 10.3389/fncom.2021.653097 PMC8489198

[43] Nguyen PL, Cho J. Pathophysiological roles of histamine receptors in cancer progression: implications and perspectives as potential molecular targets. Biomolecules. 2021;11:1232. 34439898 10.3390/biom11081232 PMC8392479

[44] Streifel KM, Gonzales AL, De Miranda B, Mouneimne R, Earley S, Tjalkens R. Dopaminergic neurotoxicants cause biphasic inhibition of purinergic calcium signaling in astrocytes. PLoS ONE. 2014;9:e110996. 25365260 10.1371/journal.pone.0110996 PMC4217743

[45] Isakov N. Protein kinase C (PKC) isoforms in cancer, tumor promotion and tumor suppression. Semin Cancer Biol. 2018;48:36–52. 28571764 10.1016/j.semcancer.2017.04.012

[46] Cooke M, Zhang X, Zhang S, Eruslanov E, Lal P, Daniel RE, et al. Protein kinase C alpha is a central node for tumorigenic transcriptional networks in human prostate cancer. Cancer Res Commun. 2022;2:1372–87. 36818489 10.1158/2767-9764.CRC-22-0170 PMC9933888

[47] Zhang N, Dou Y, Liu L, Zhang X, Liu X, Zeng Q, et al. SA-49, a novel aloperine derivative, induces MITF-dependent lysosomal degradation of PD-L1. EBioMedicine. 2019;40:151–62. 30711516 10.1016/j.ebiom.2019.01.054 PMC6414307

[48] Liu C, Liu D, Wang S, Gan L, Yang X, Ma C. Identification of the SNARE complex that mediates the fusion of multivesicular bodies with the plasma membrane in exosome secretion. J Extracell Vesicles. 2023;12:e12356. 37700095 10.1002/jev2.12356 PMC10497535

[49] Rhee SH, Jones BW, Toshchakov V, Vogel SN, Fenton MJ. Toll-like receptors 2 and 4 activate STAT1 serine phosphorylation by distinct mechanisms in macrophages. J Biol Chem. 2003;278:22506–12. 12686553 10.1074/jbc.M208633200

[50] Oliva JL, Caino MC, Senderowicz AM, Kazanietz MG. S-Phase-specific activation of PKC alpha induces senescence in non-small cell lung cancer cells. J Biol Chem. 2008;283:5466–76. 18162471 10.1074/jbc.M707576200

[51] Akakura S, Nochajski P, Gao L, Sotomayor P, Matsui S, Gelman IH. Rb-dependent cellular senescence, multinucleation and susceptibility to oncogenic transformation through PKC scaffolding by SSeCKS/AKAP12. Cell Cycle. 2010;9:4656–65. 21099353 10.4161/cc.9.23.13974 PMC3048035

[52] Wang C, Wang X, Liang H, Wang T, Yan X, Cao M, et al. miR-203 inhibits cell proliferation and migration of lung cancer cells by targeting PKCalpha. PLoS ONE. 2013;8:e73985. 24040137 10.1371/journal.pone.0073985 PMC3769394

[53] Hill KS, Erdogan E, Khoor A, Walsh MP, Leitges M, Murray NR, et al. Protein kinase Calpha suppresses Kras-mediated lung tumor formation through activation of a p38 MAPK-TGFbeta signaling axis. Oncogene. 2014;33:2134–44. 23604119 10.1038/onc.2013.147 PMC3895109

[54] Salama MF, Liu M, Clarke CJ, Espaillat MP, Haley JD, Jin T, et al. PKCalpha is required for Akt-mTORC1 activation in non-small cell lung carcinoma (NSCLC) with EGFR mutation. Oncogene. 2019;38:7311–28. 31420605 10.1038/s41388-019-0950-z PMC6883150

[55] Chatterjee E, Chaudhuri RD, Sarkar S. Cardiomyocyte targeted overexpression of IGF1 during detraining restores compromised cardiac condition via mTORC2 mediated switching of PKCdelta to PKCalpha. Biochim Biophys Acta Mol Basis Dis. 2019;1865:2736–52. 31299218 10.1016/j.bbadis.2019.07.003

[56] Serrano-Lopez EM, Coronado-Parra T, Marin-Vicente C, Szallasi Z, Gomez-Abellan V, Lopez-Andreo MJ, et al. Deciphering the role and signaling pathways of PKCalpha in luminal A breast cancer cells. Int J Mol Sci. 2022;23:14023. 36430510 10.3390/ijms232214023 PMC9696894

[57] Smith SD, Enge M, Bao W, Thullberg M, Costa TD, Olofsson H, et al. Protein kinase Calpha (PKCalpha) regulates p53 localization and melanoma cell survival downstream of integrin alphav in three-dimensional collagen and in vivo. J Biol Chem. 2012;287:29336–47. 22773839 10.1074/jbc.M112.341917 PMC3436133

