# Supplementary material for: PKCα regulates the secretion of PDL1-carrying small extracellular vesicles in a p53-dependent manner
Source: Cell Death Dis. 2025 Jan 14;16(1):19. doi: 10.1038/s41419-025-07341-5 (PMC11733117; doi:10.1038/s41419-025-07341-5)
Supplement: Supplementary file 9 — Supplement Figure and legend Cell Death X Disease [file 41419_2025_7341_MOESM9_ESM.pdf]

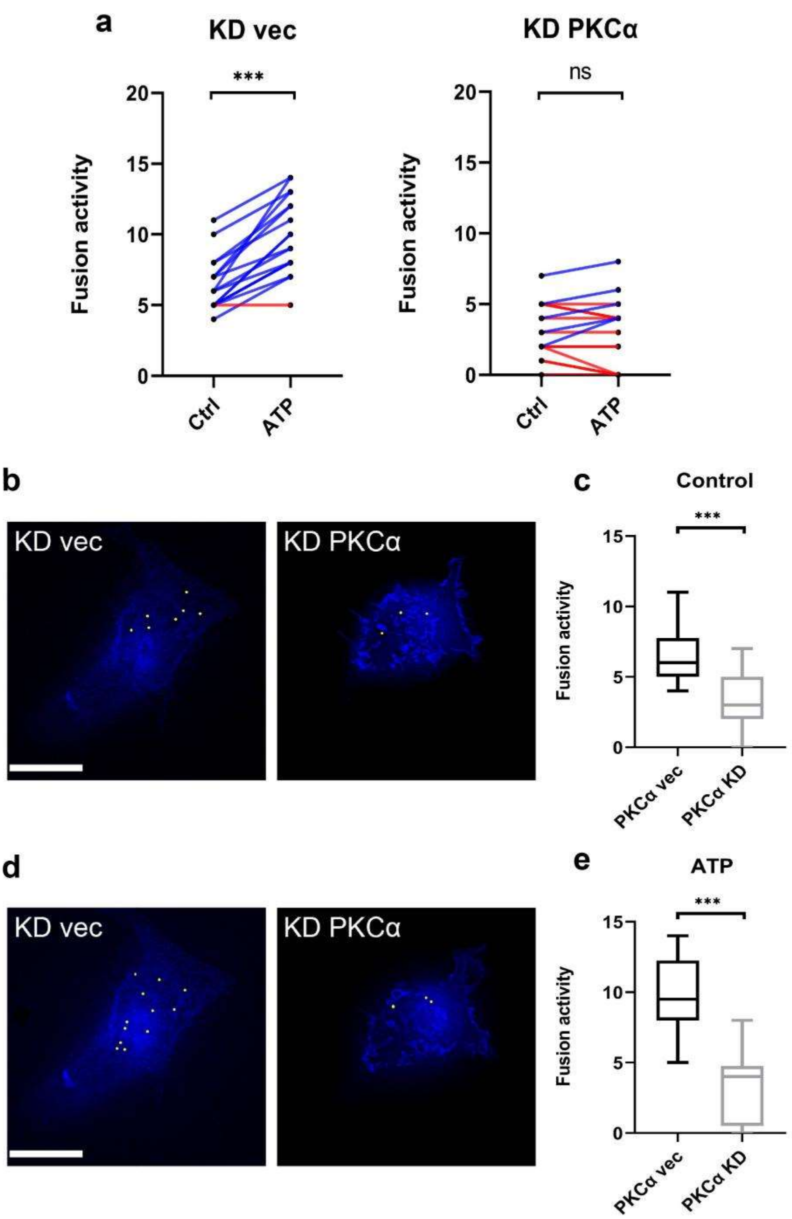

Figure S1 PKCα knock down effects on PD-L1 fusion activity in H1975 cells overexpressing PD-L1-pHluorin

a) Measurement of fusion activity of ATP-stimulated cells transfected with control shRNA and PKCα shRNA. n ≥ 3 cells per condition. b) Cumulative representation of fusion events throughout a 180-second time at resting condition in cells transfected with control shRNA and PKCα shRNA. Bar, 20 μm. c) Quantification of basal fusion activity at resting condition in cells transfected with control shRNA and PKCα shRNA. d) Cumulative representation of fusion events throughout a 180-second time at ATP stimulation condition in cells transfected with control shRNA and PKCα shRNA. Bar, 20 μm. e) Quantification of fusion activity at ATP stimulation condition in cells transfected with control shRNA and PKCα shRNA. *, P<0.05; **, P<0.01; ***, P<0.001 using paired Student's t test.

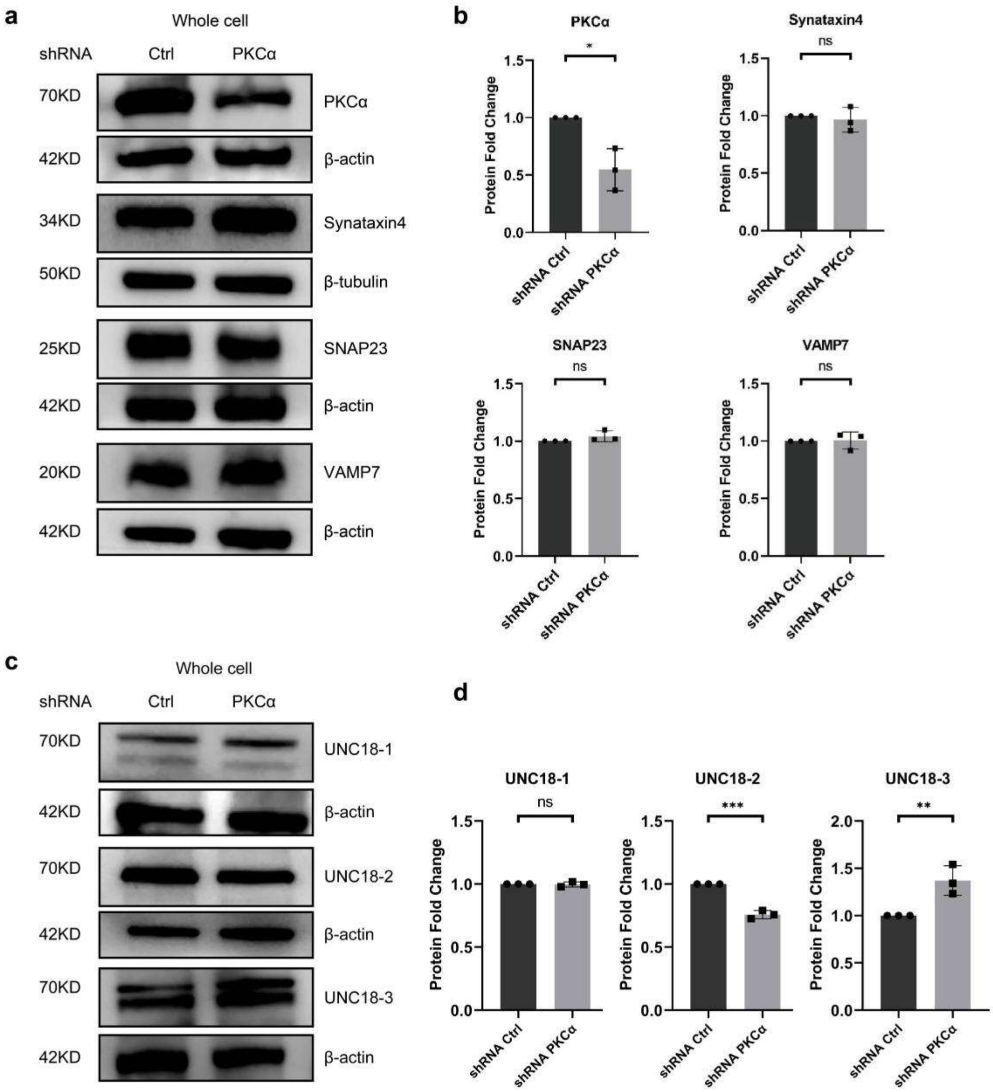

Figure S2 PKCα knockdown selectively affects the expression of Munc18 isoforms but not key SNARE proteins in H1299 cells.

a) Immunoblot analysis of whole cell lysates from H1299 cells with shRNA control (Ctrl) or PKCα knockdown, showing expression levels of PKCα, Syntaxin4, SNAP23, and VAMP7. β-tubulin and β-actin were used as loading controls. b) Quantification of each blot of PKCα, Syntaxin4, SNAP23, and VAMP7 protein expression normalized to loading control (n = 3). c) Immunoblot analysis of UNC18 isoforms (UNC18-1, UNC18-2, and UNC18-3) in whole cell lysates of H1299 cells with shRNA control or PKCα knockdown. β-actin was used as a loading control. d) Quantification of UNC18 isoforms protein expression normalized to loading control (n = 3). Statistic data are presented as means ± SD.

*, $P < 0.05$; **, $P < 0.01$; ***, $P < 0.001$ using Student's t test.

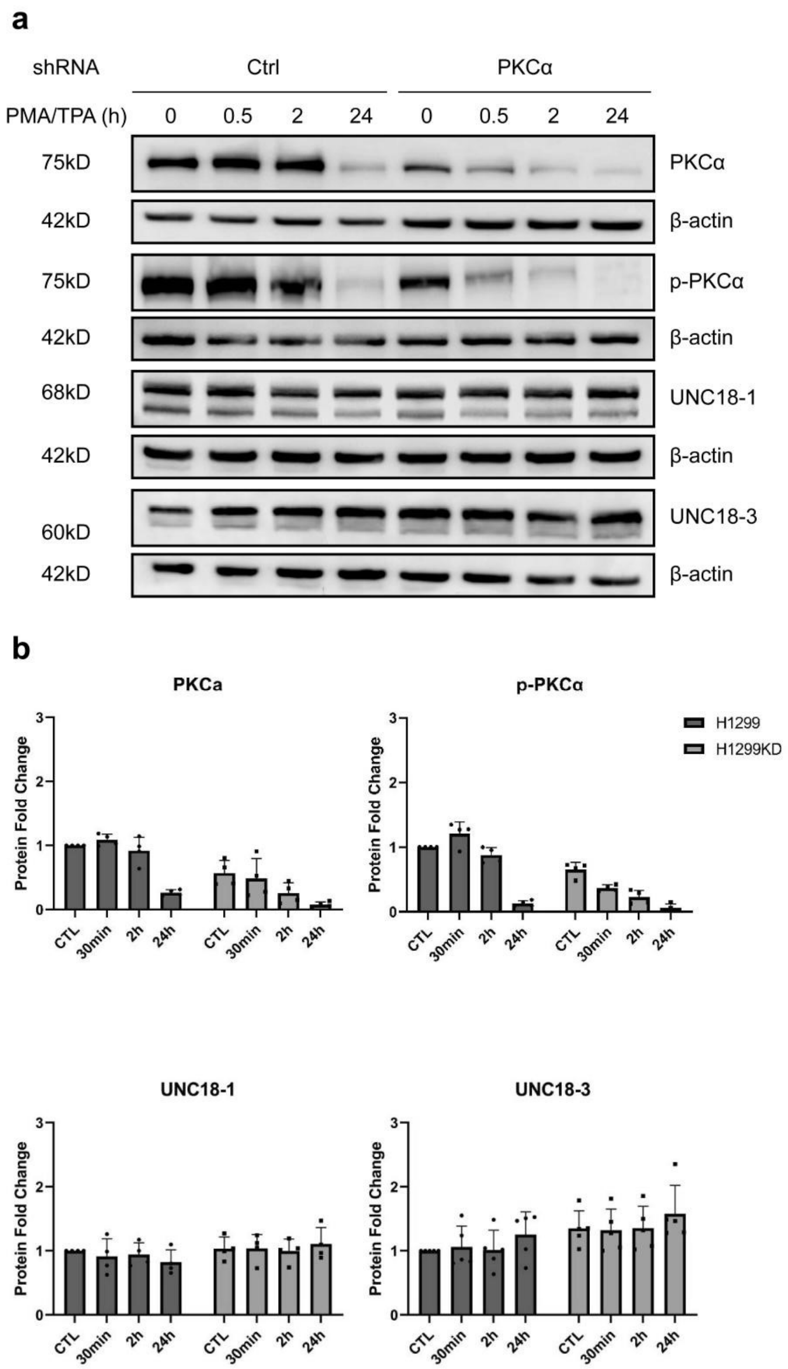

Figure S3 KCα knockdown alters UNC18 isoform expression dynamics following PMA/TPA stimulation in H1299 cells.

a) Immunoblot analysis of PKCα, phosphorylated PKCα (p-PKCα), UNC18-1, and UNC18-3 protein expression in H1299 cells with shRNA control (Ctrl) or PKCα knockdown after stimulation with 1μM PMA/TPA for 0, 0.5, 2, and 24 hours. β-actin was used as a loading control. b) Quantification of protein levels of PKCα, p-PKCα, UNC18-1 and UNC18-3 normalized to loading control ($n \geq 3$). Statistic data are presented as means ± SD.

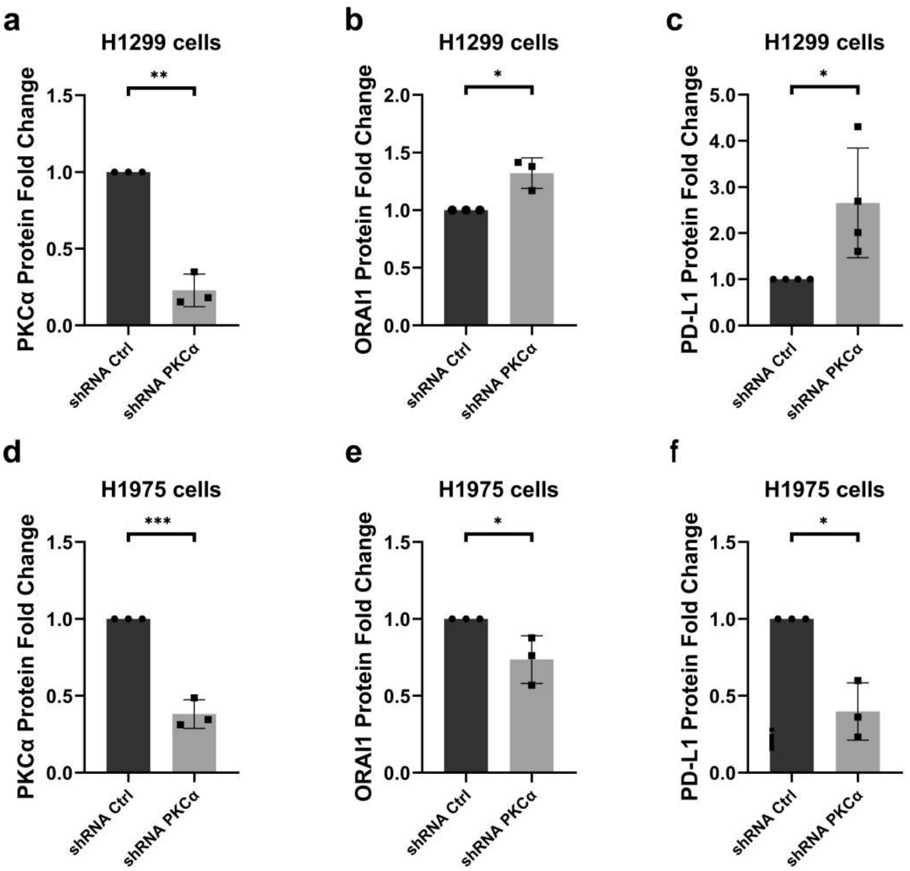

Figure S4 Quantification statistic of immunoblot analysis in Figure 5 a-f

a, b, c) Quantification of each blot of PKCα, ORAI1 and PD-L1 protein expression normalized to loading control in H1299 cells ($n \geq 3$). d, e, f) Quantification of each blot of PKCα, ORAI1 and PD-L1 protein expression normalized to loading control in H1299 cells ($n = 3$). Statistic data are presented as means ± SD.

*, $P < 0.05$; **, $P < 0.01$; ***, $P < 0.001$ using Student's t test.

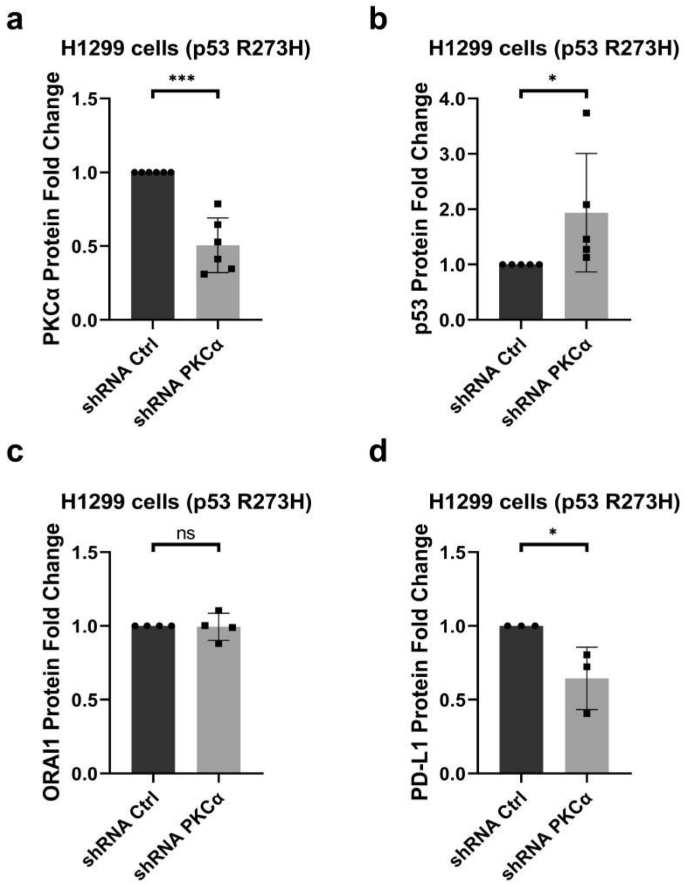

Figure S5 Quantification statistic of immunoblot analysis in Figure 5 g-i

a, b) Quantification of each blot of PKCα, p53 protein expression normalized to loading control in p53 R273H overexpression H1299 cells transfected with control shRNA and PKCα shRNA (n = 6). c, d) Quantification of each blot ORAI1and PD-L1 protein expression normalized to loading control in p53 R273H overexpression H1299 cells transfected with control shRNA and PKCα shRNA (n ≥ 3). Statistic data are presented as means ± SD.

*, P < 0.05; **, P < 0.01; ***, P < 0.001 using Student's t test.

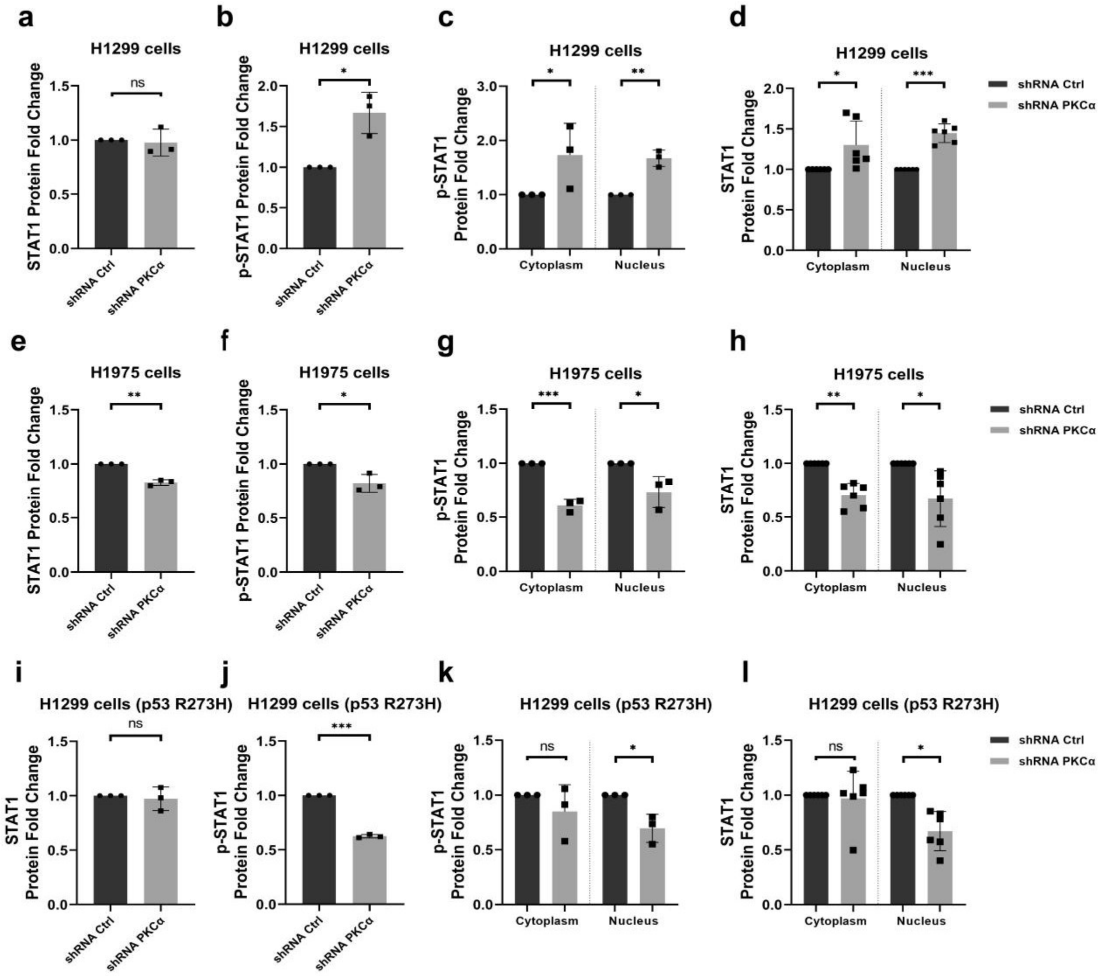

Figure S6 Quantification statistic of immunoblot analysis in Figure 6

a, b) Quantification of each blot of pSTAT1(Y701) and STAT1 protein expression normalized to loading control in H1299 cells (n = 3). c, d) Quantification of pSTAT1(Y701) and STAT1 protein levels, normalized to cytoplasmic and nuclear loading controls in H1299 cells (n ≥ 3). e, f) Quantification of each blot of pSTAT1(Y701) and STAT1 protein expression normalized to loading control in H1975 cells (n = 3). g, h) Quantification of pSTAT1(Y701) and STAT1 protein levels, normalized to cytoplasmic and nuclear loading controls in H1975 cells (n ≥ 3). i, j) Quantification of each blot of pSTAT1(Y701) and STAT1 protein expression normalized to loading control in H1299 cells overexpressed p53 R273H (n = 3). k, l) Quantification of pSTAT1(Y701) and STAT1 protein levels, normalized to cytoplasmic and nuclear loading controls in H1299 cells overexpressed p53 R273H (n ≥ 3). Statistic data are presented as means ± SD.

*, $P < 0.05$; **, $P < 0.01$; ***, $P < 0.001$ using Student's t test.

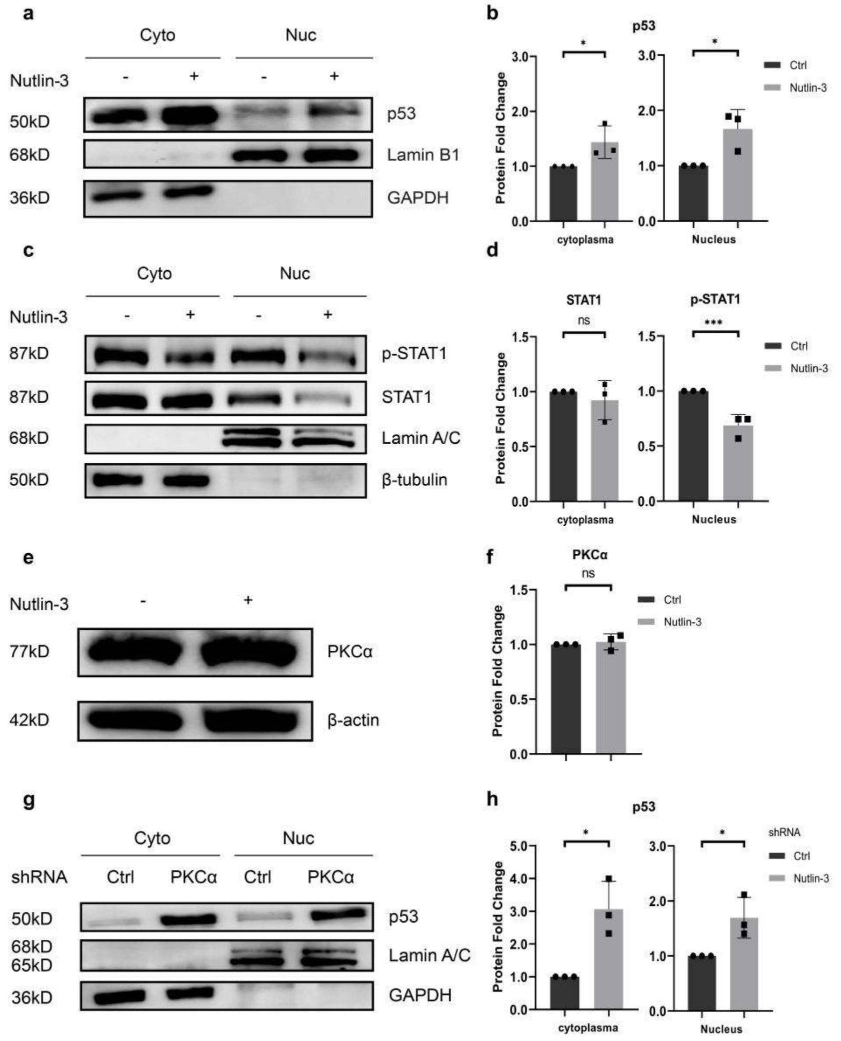

Figure S7 PKCα modulates STAT1 via p53 regulation in H1299-p53 R273H cells

a) Immunoblot analysis of p53 protein expression in both cytoplasm and nucleus fractions from p53 R273H overexpression H1299 cells (H1299-p53 R273H cells) treated with or without 15 μM Nutlin-3 for 24 h. GAPDH and Lamin B1 were used as cytoplasmic and nuclear markers, respectively. b) Quantification of each blot of p53 protein expression normalized to loading control (n = 3). c) Immunoblot analysis of p-STAT1 (Y701) and STAT1 protein expression in both cytoplasm and nucleus fractions from H1299-p53 R273H cells treated with or without 15 μM Nutlin-3 for 24 h. β-tubulin and Lamin B1 were used as loading controls for cytoplasmic and nuclear fractions, respectively. d) Quantification of each blot of p-STAT1 (Y701) protein expression normalized to nuclear loading control Lamin A/C (n = 3) and STAT1 protein expression normalized to cytoplasmic loading control β-tubulin (n = 3). e) Immunoblot analysis on PKCα protein expression after treating with 15 μM Nutlin-3 in H1975 cells for 24 h. f) Quantification of each blot of PKCα protein expression normalized to β-actin (n = 3). g) Immunoblot analysis of p53 protein expression in the cytoplasm and nucleus of H1299-p53 R273H cells with shRNA control or PKCα knockdown. GAPDH serves as a cytoplasmic loading control, and Lamin A/C as a nuclear loading control. h) Quantification of each blot of p53 protein expression normalized to corresponding loading control (n = 3). Statistic data are presented as means ± SD.

*, P<0.05; **, P<0.01; ***, P<0.001 using using Student's t test.

Video 1 shows time-lapse imaging of PD-L1-pHluorin in H1299 cells at 12× normal speed.

Video 2 shows the time-lapse imaging of typical MVB-PM fusion process of PD-L1 at 12× normal speed.

Video 3 shows the time-lapse imaging of typical MVB-PM fusion process of CD63 at 12× normal speed.

Video 4 shows the time-lapse imaging of typical MVB-PM fusion process of CD81 at 12× normal speed.

Video 5 shows the time-lapse imaging of typical MVB-PM fusion process of CD9 at 12× normal speed.

Video 6 shows the calcium changes induced by ATP stimulation indicated by H1299-GCaMP6s in normal at 50× normal speed.

Video 7 shows the calcium changes induced by ATP stimulation indicated by H1299-GCaMP6s pretreated with EGTA at 50× normal speed.

Video 8 shows the calcium changes induced by ATP stimulation indicated by H1299-GCaMP6s pretreated with BAPTA-AM at 50× normal speed.
