# Supplementary material for: PKCα regulates the secretion of PDL1-carrying small extracellular vesicles in a p53-dependent manner
Source: Cell Death Dis. 2025 Jan 14;16(1):19. doi: 10.1038/s41419-025-07341-5 (PMC11733117; doi:10.1038/s41419-025-07341-5)

Source Data Fig.1

Fig.1 f left

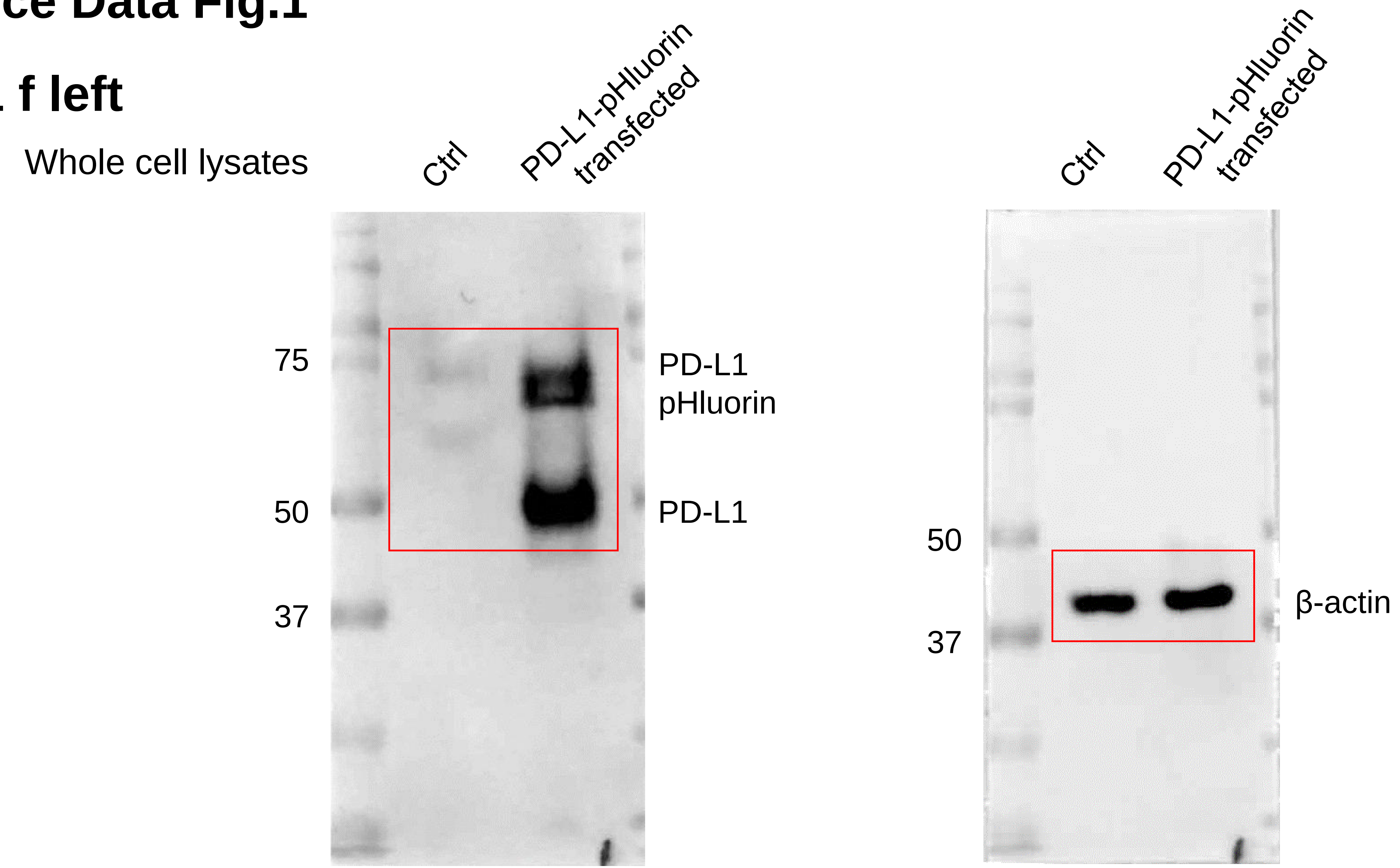

Fig.1 f right

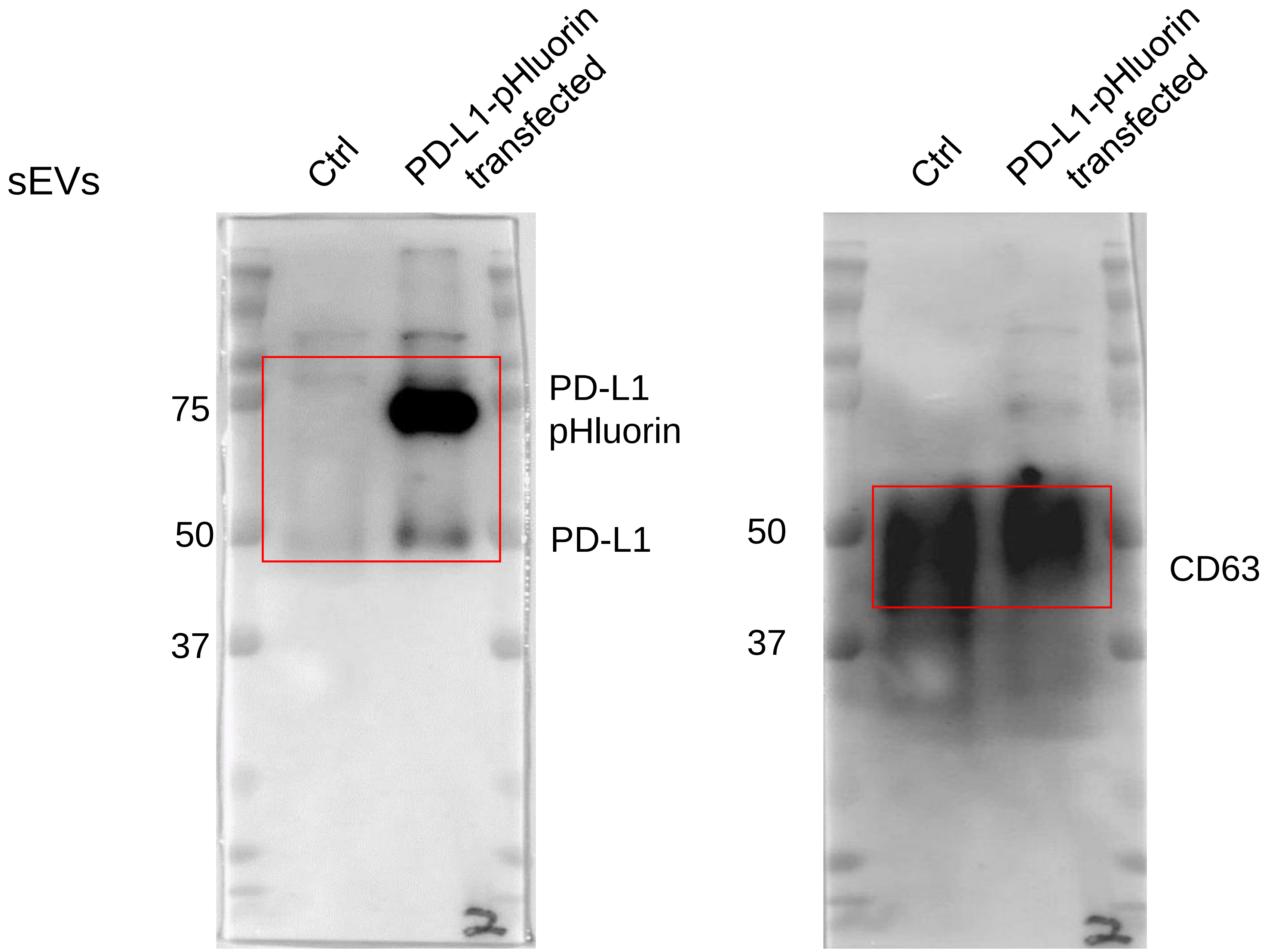
sEVs
Ctrl
PD-L1-pHluorin
transfected
75
50
37
PD-L1
pHluorin
PD-L1
Ctrl
PD-L1-pHluorin
transfected
50
37
CD63

Source Data Fig.4

Cell line: H1299-PD-L1-pHluorin

Whole cell lysates

Fig.4 d

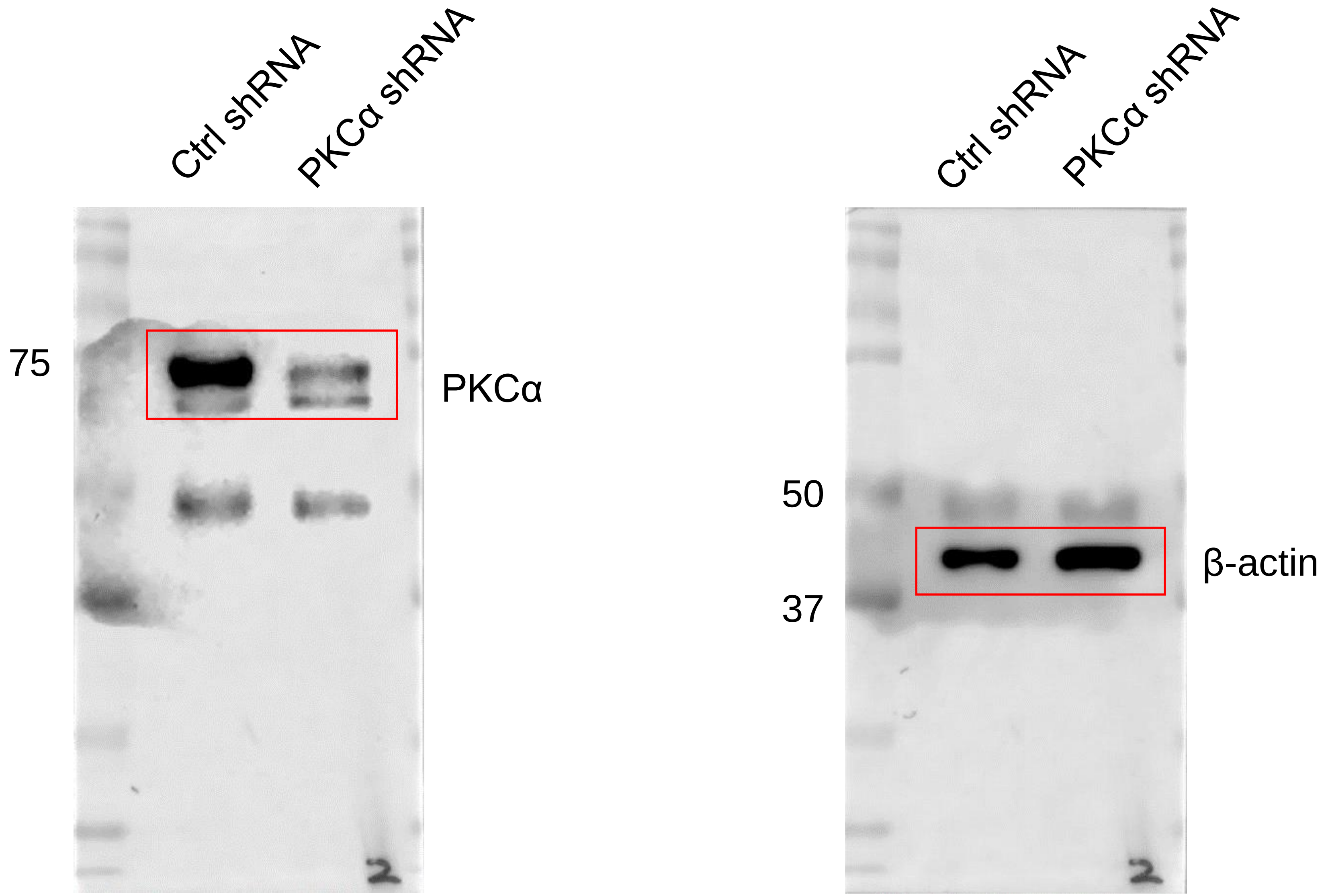

Source Data Fig.4

Cell line: H1299-PD-L1-pHluorin

sEVs

Fig.4 i

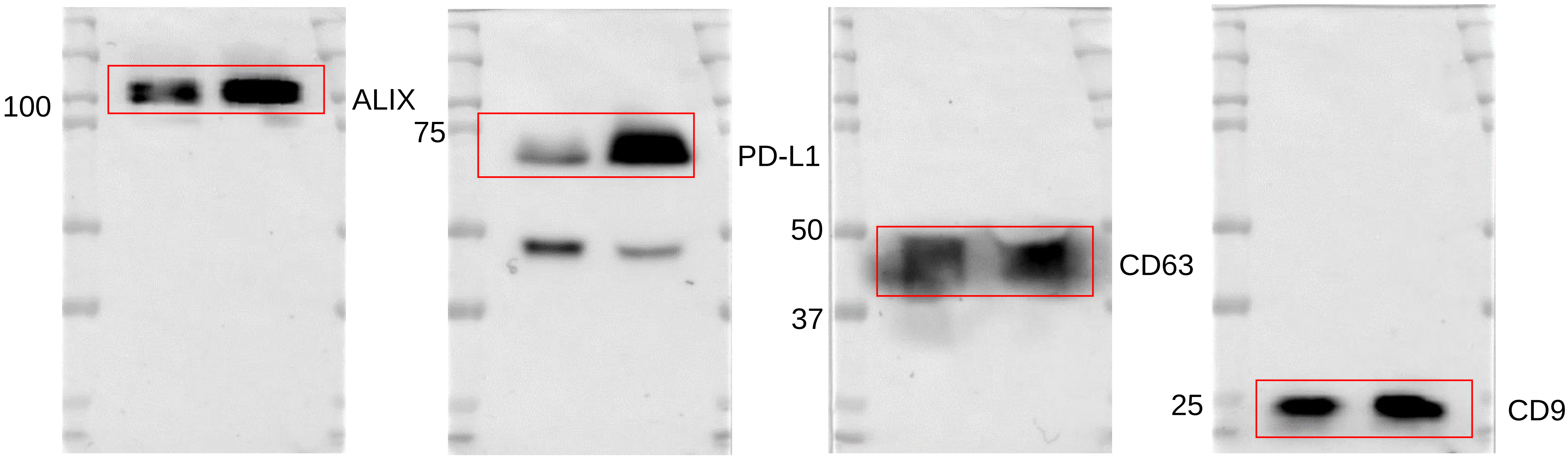

Source Data Fig.5

Fig.5 a

Cell line: H1299

Whole cell lysates

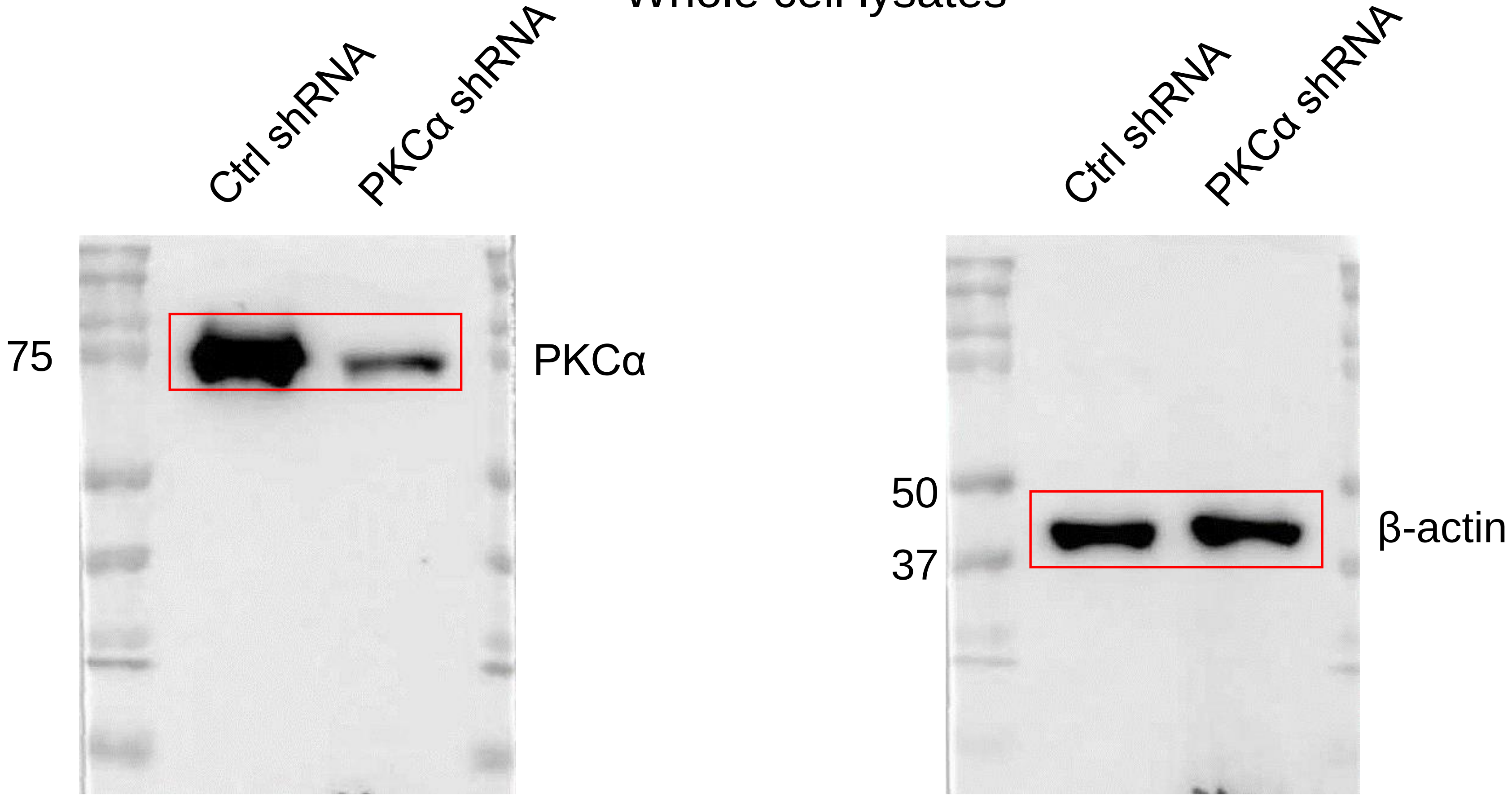

Source Data Fig.5

Cell line: H1299

sEVs

Fig.5 b

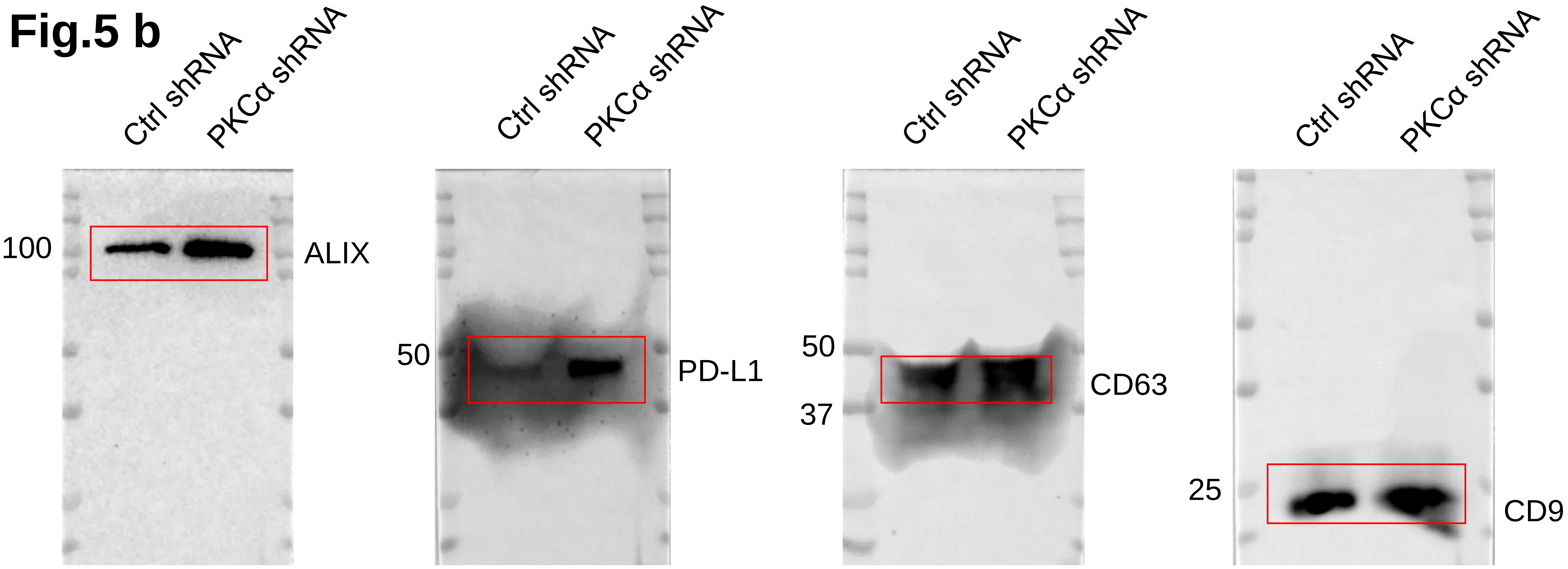

Source Data Fig.5

Cell line: H1299
Whole cell lysates

Fig.5 c

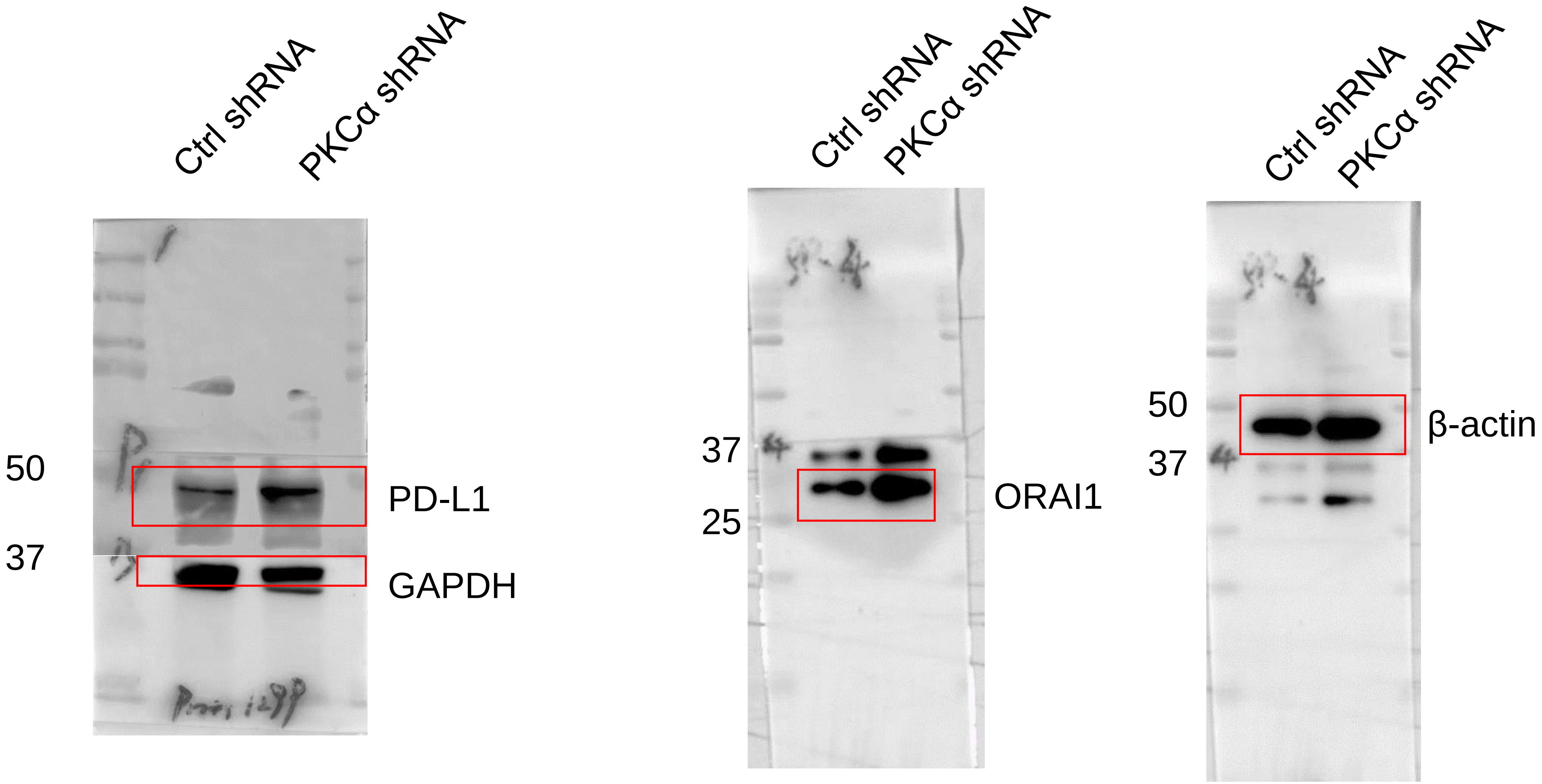

Source Data Fig.5

Cell line: H1975

Whole cell lysates

Fig.5 d

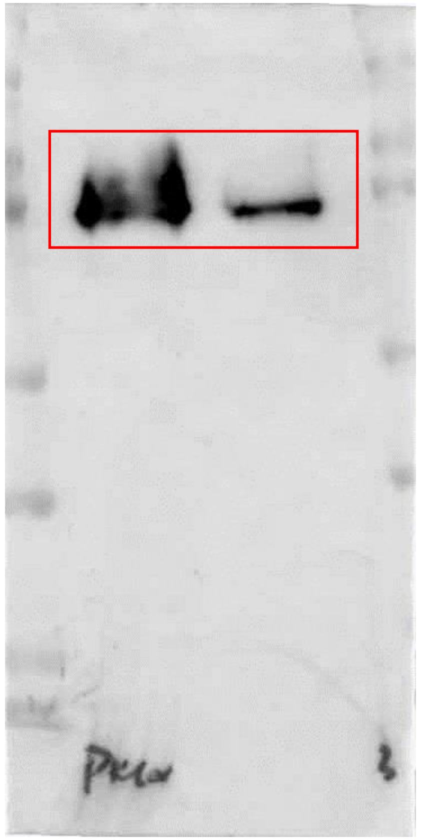

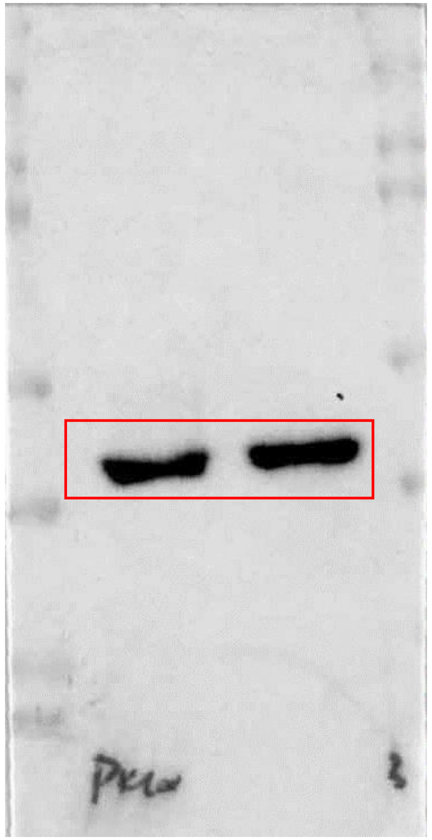

Source Data Fig.5

Cell line: H1975

sEVs

Fig.5 e

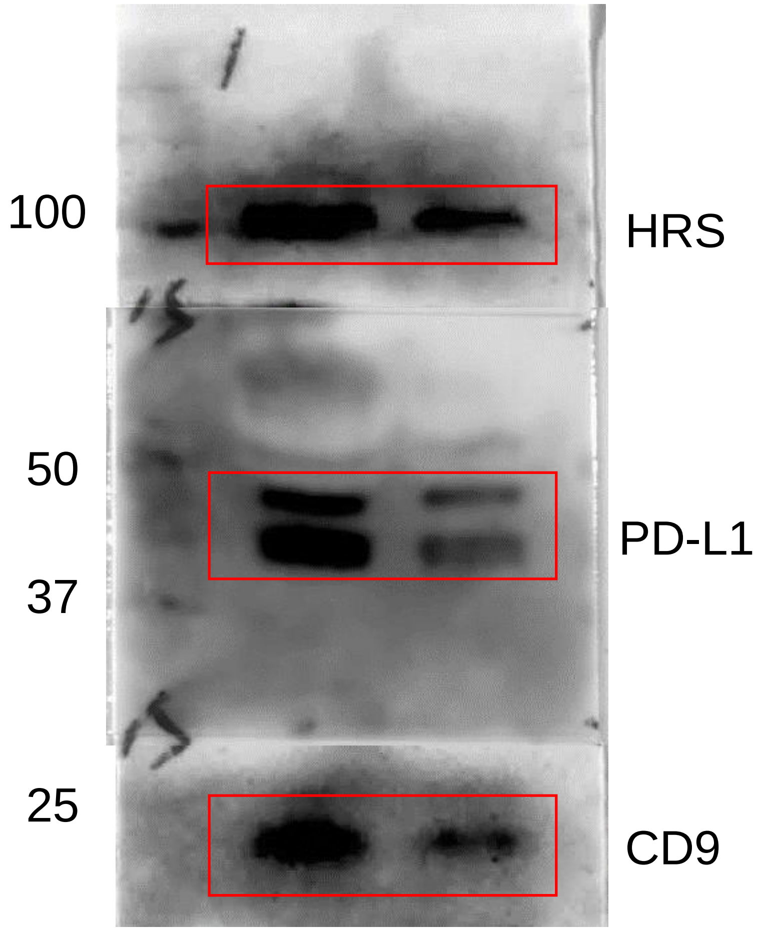

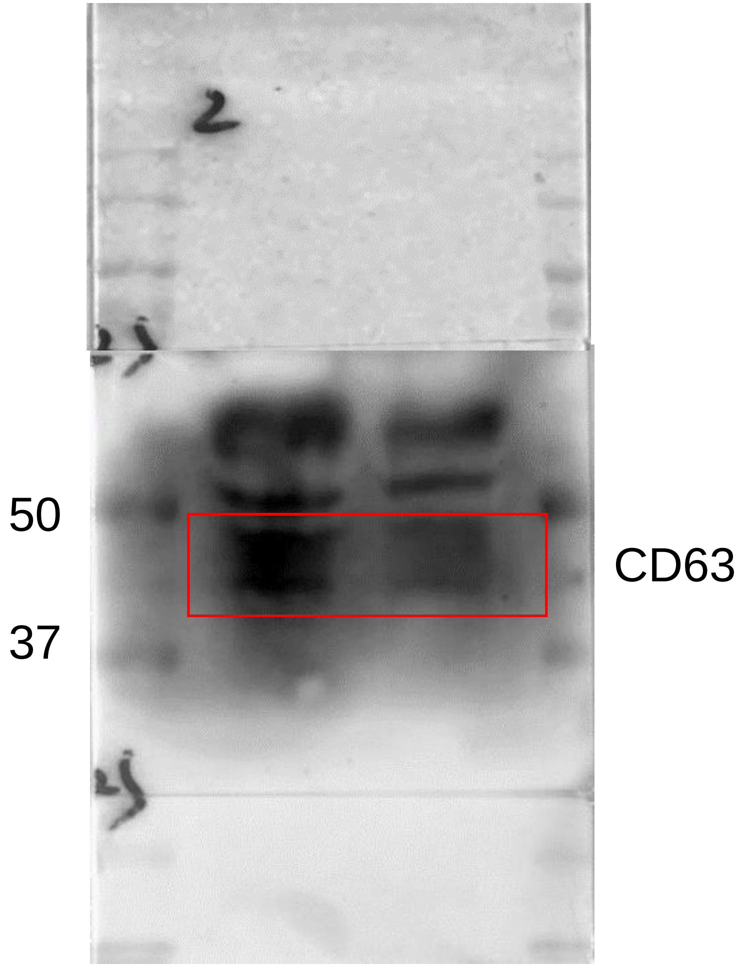

Source Data Fig.5

Cell line: H1975

Whole cell lysates

Fig.5 f

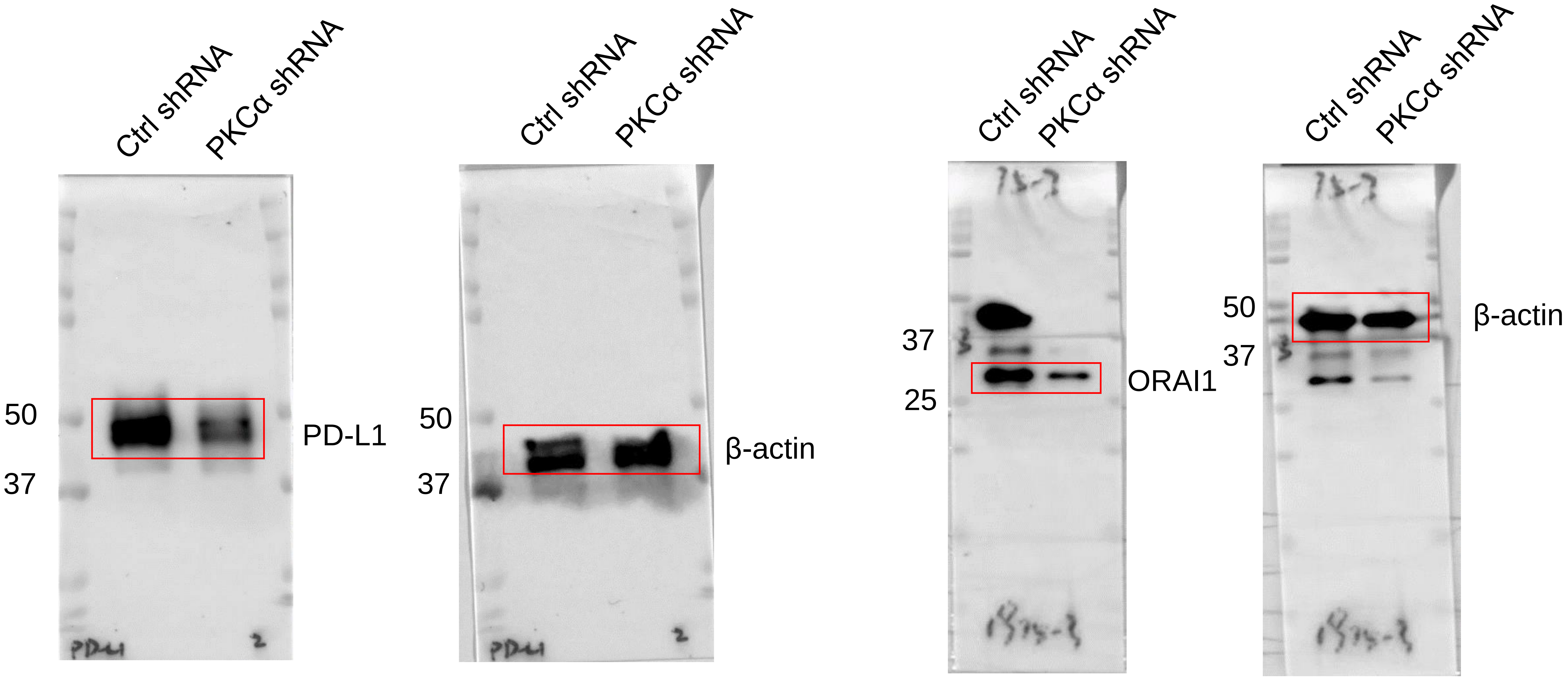

Source Data Fig.5

Cell line: H1299

Whole cell lysates

Fig.5 g

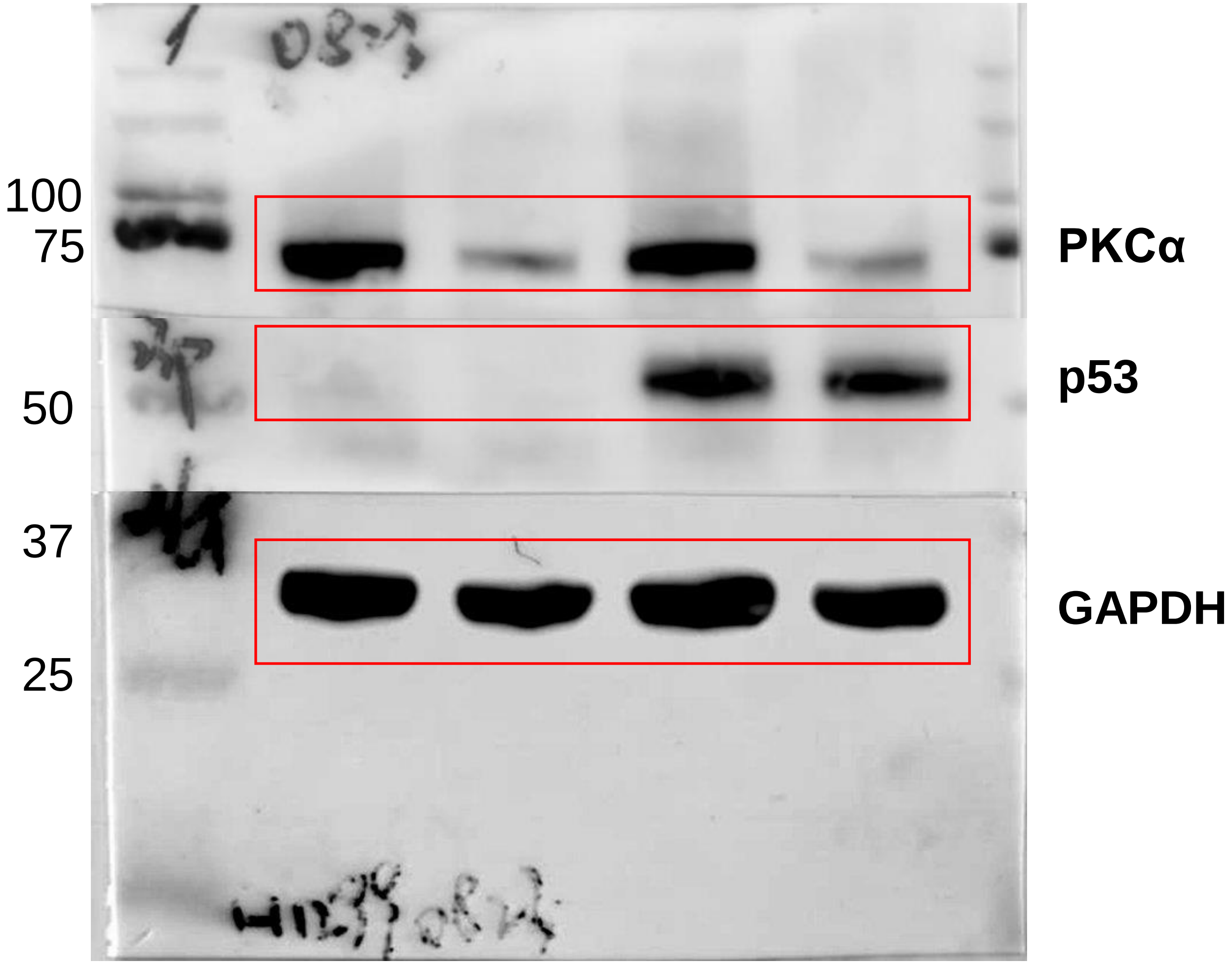

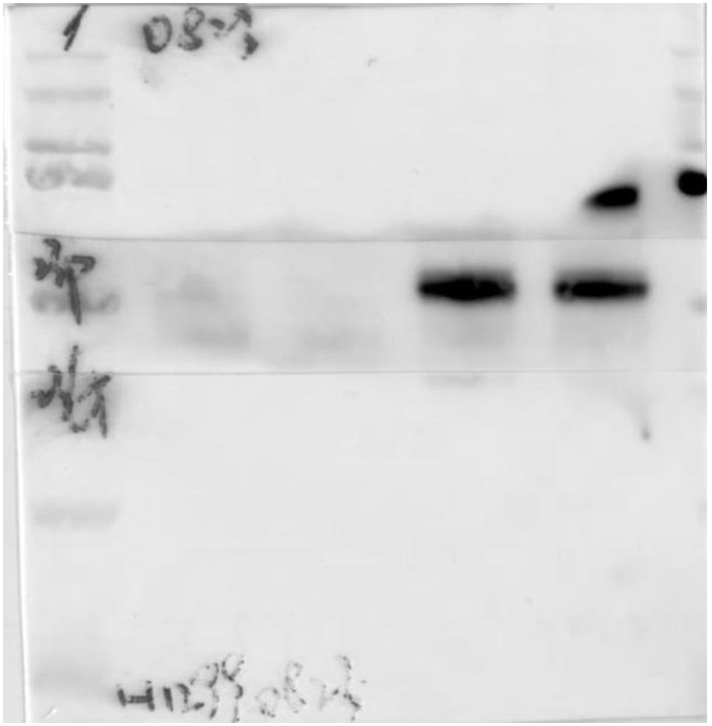

Source Data Fig.5

Cell line: H1299 p53 R273H

Whole cell lysates

Fig.5 h

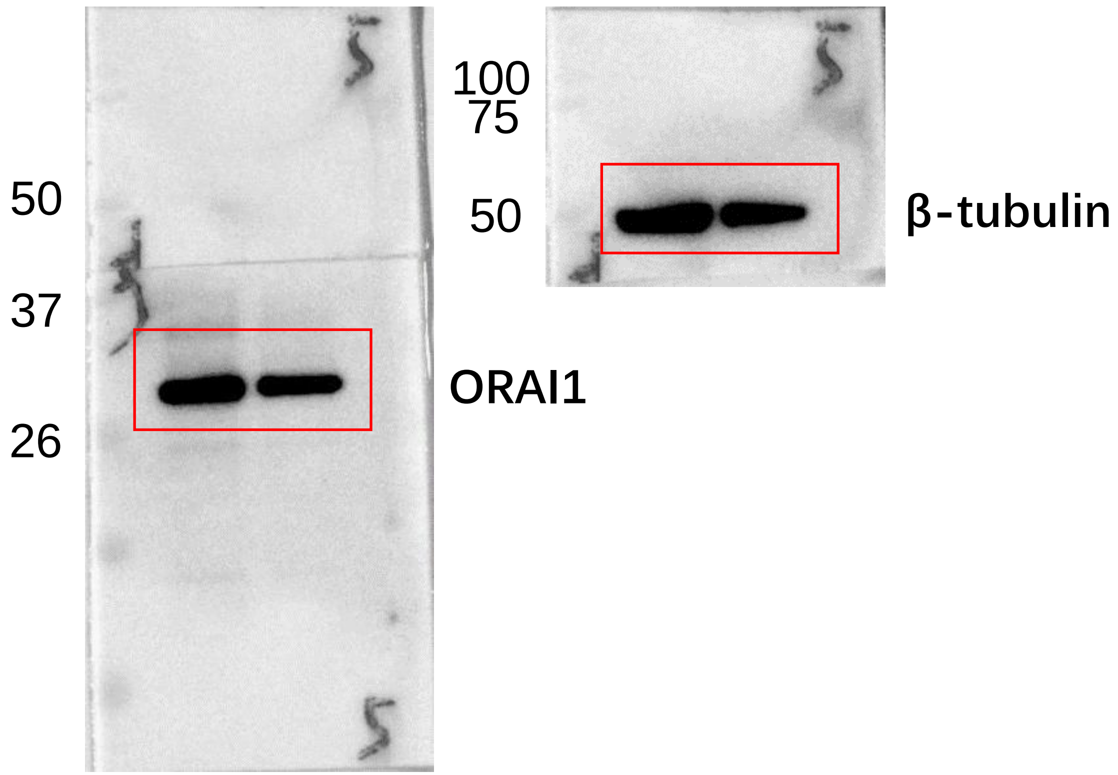

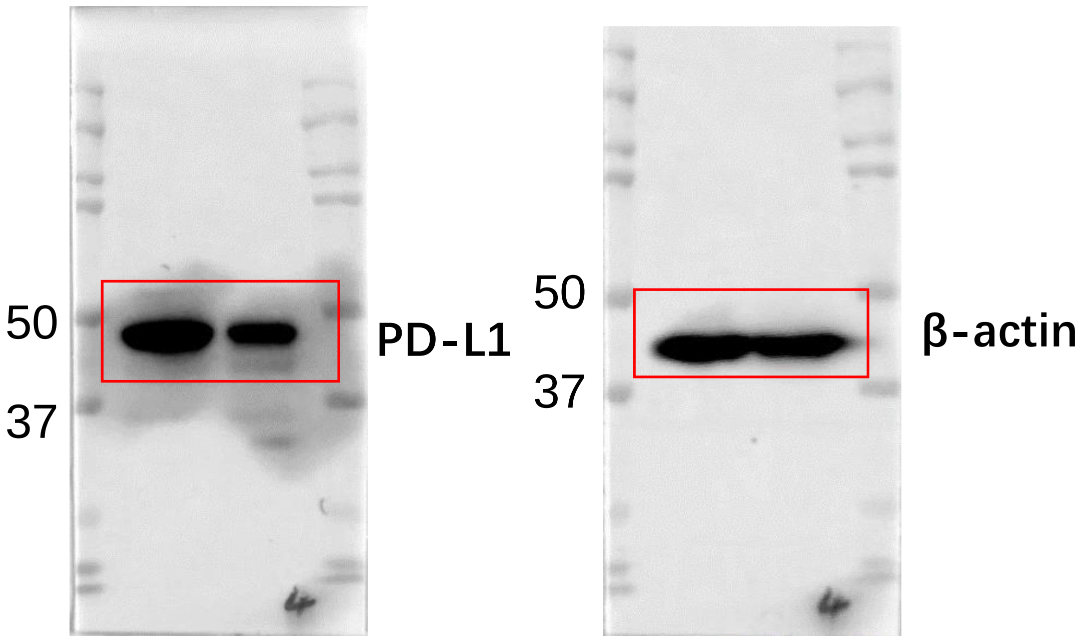

Source Data Fig.5

Cell line: H1299 p53 R273H

sEVs

Fig.5 i

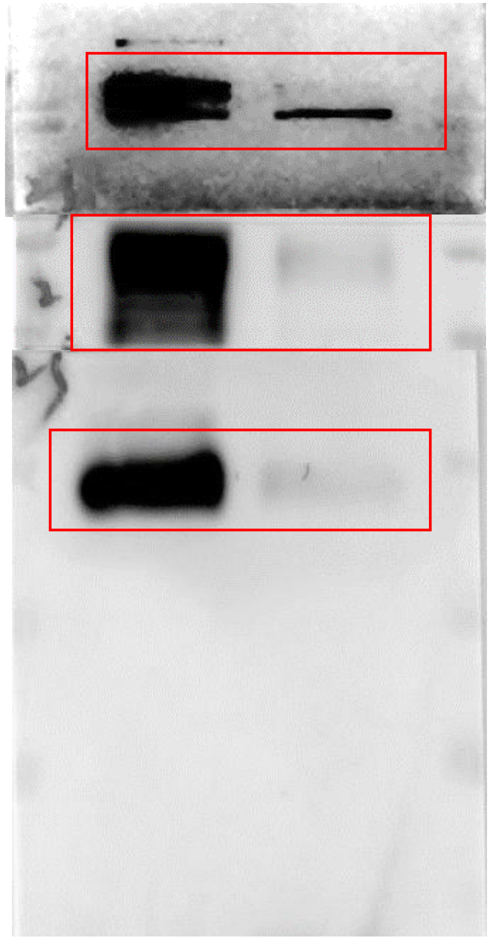

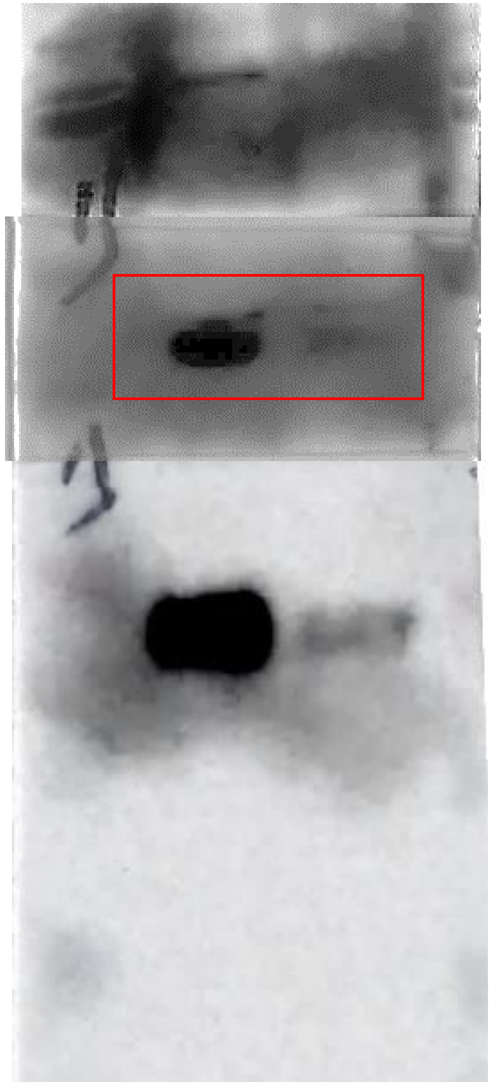

Source Data Fig.6

Fig.6 e

Cell line: H1299

Whole cell lysates

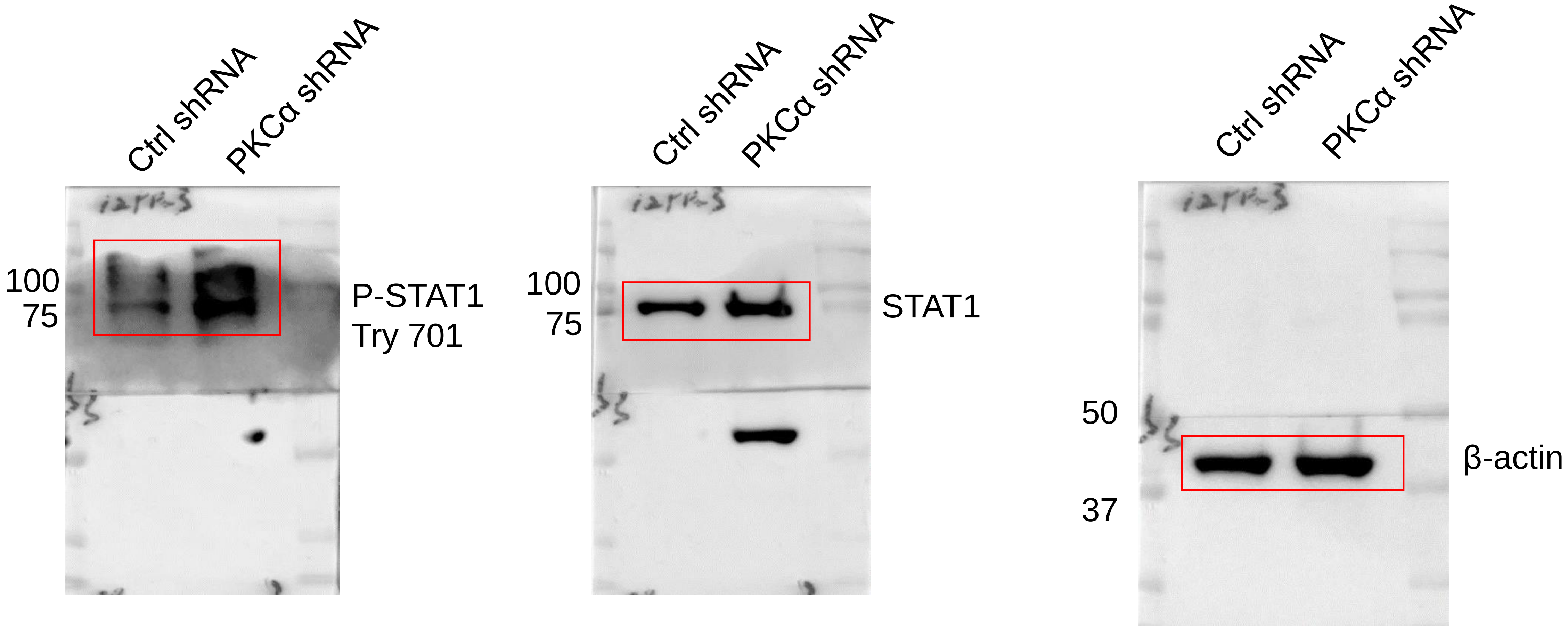

Source Data Fig.6

Cell line: H1299

Fig.6 f

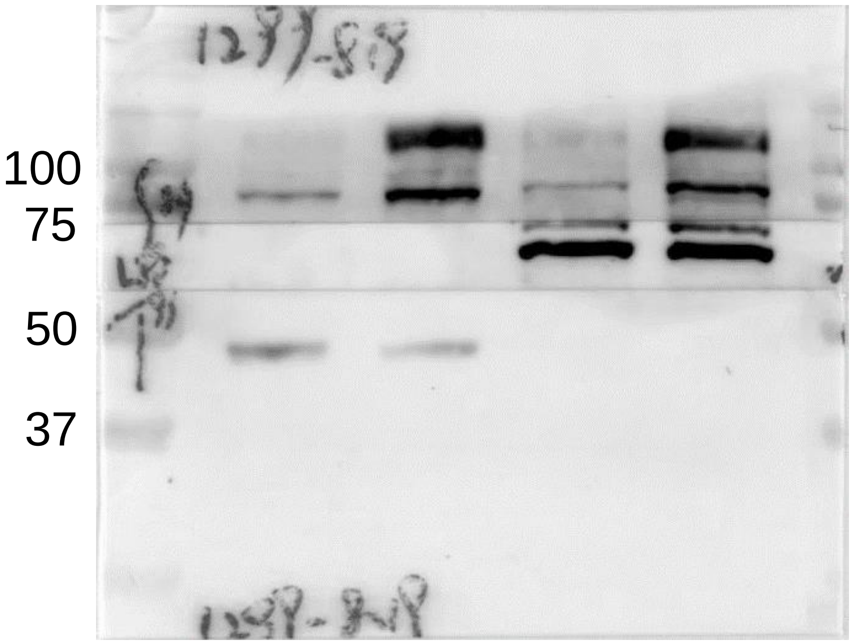

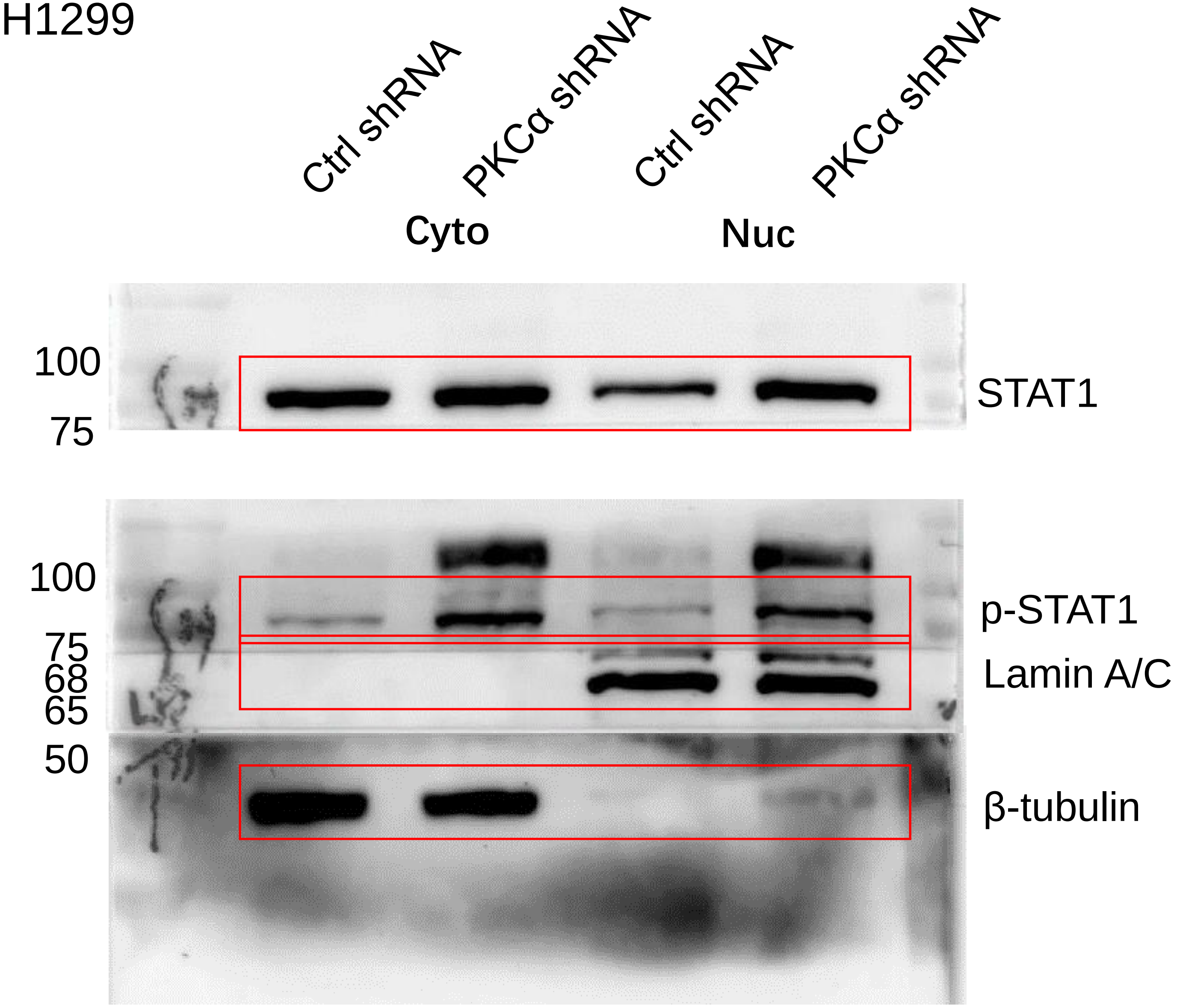

Source Data Fig.6

Cell line: H1975
Whole cell lysates

Fig.6 g

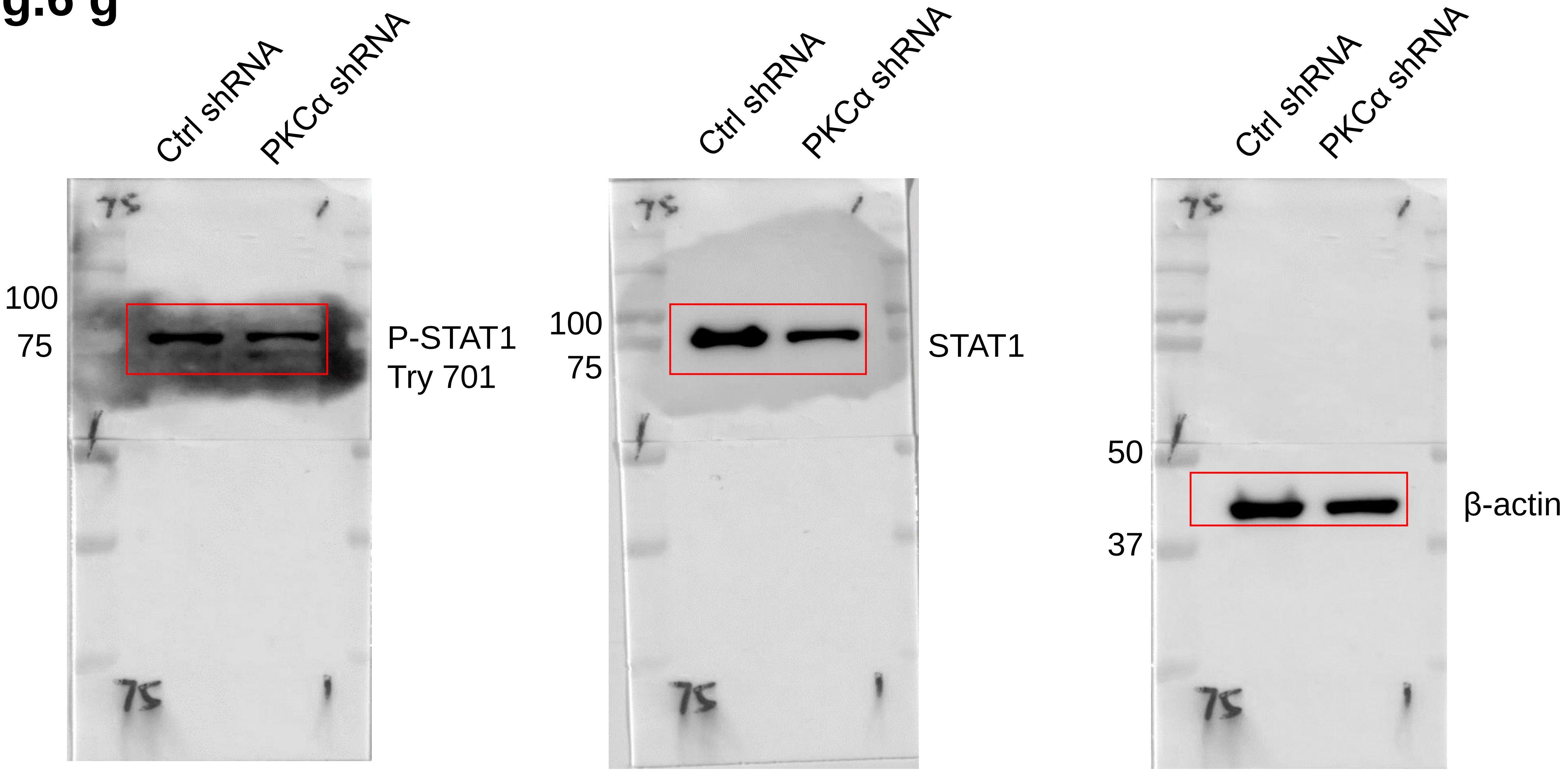

Source Data Fig.6

Cell line: H1975

Fig.6 h

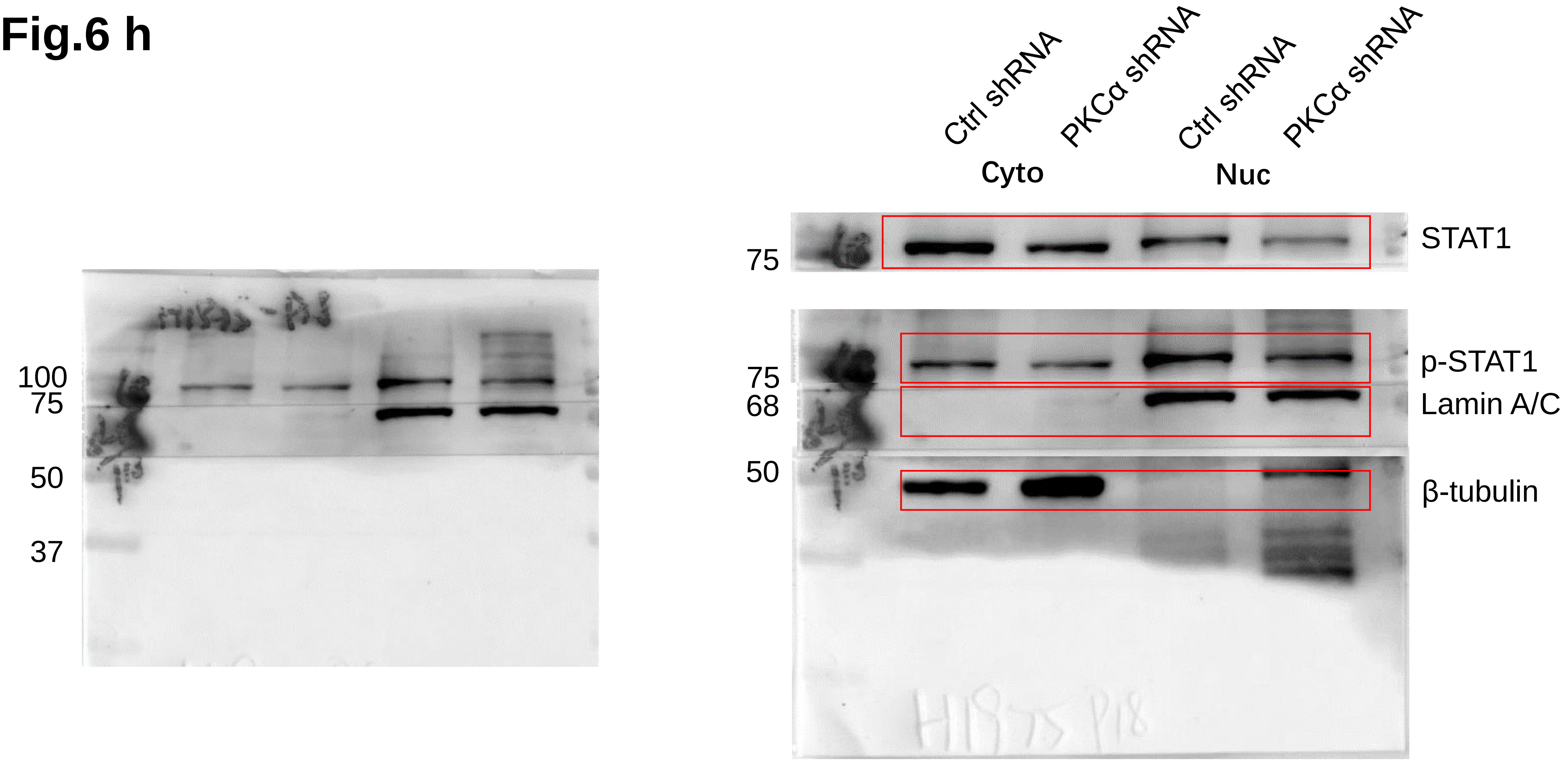

Source Data Fig.6

Cell line: H1299-p53 R273H cell
Whole cell lysates

Fig.6 i

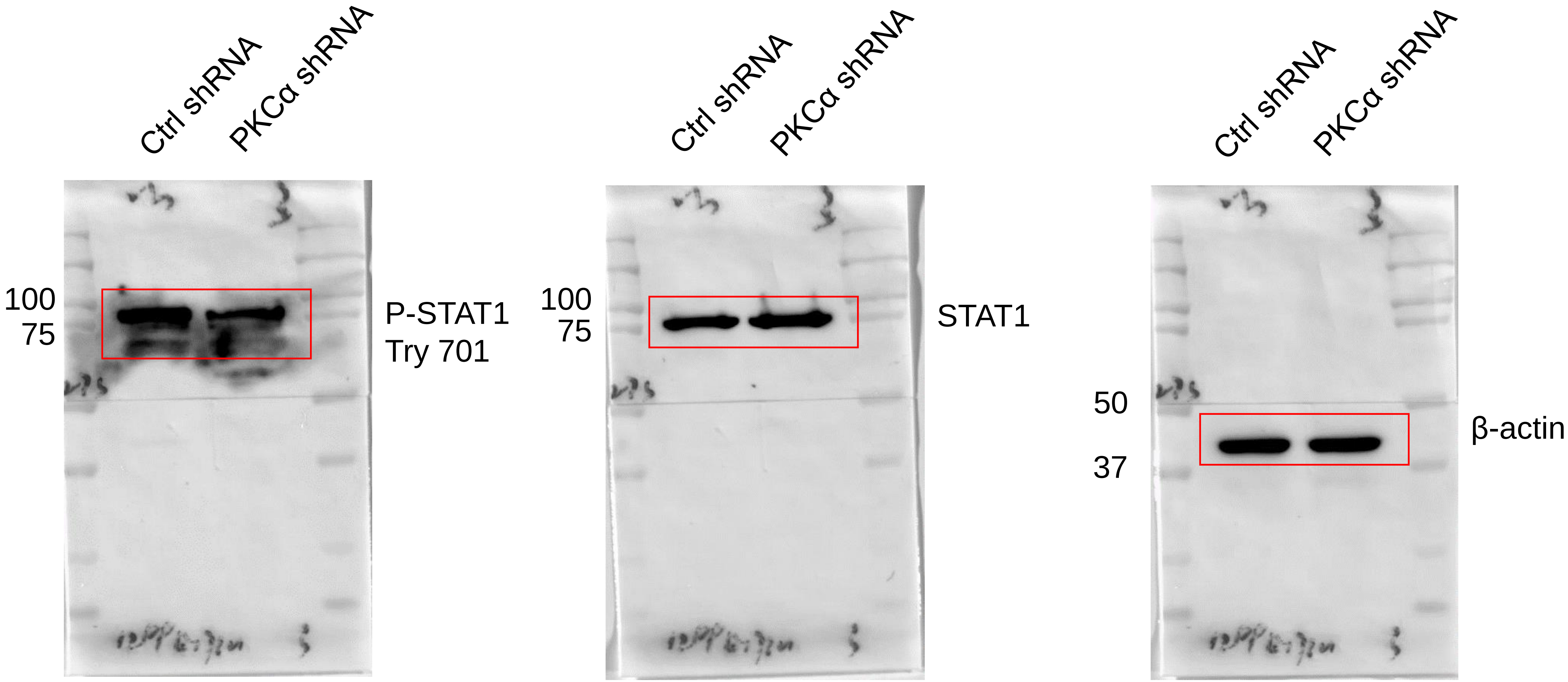

Source Data Fig.6

Fig.6 j

Cell line: H1299-p53 R273H cell

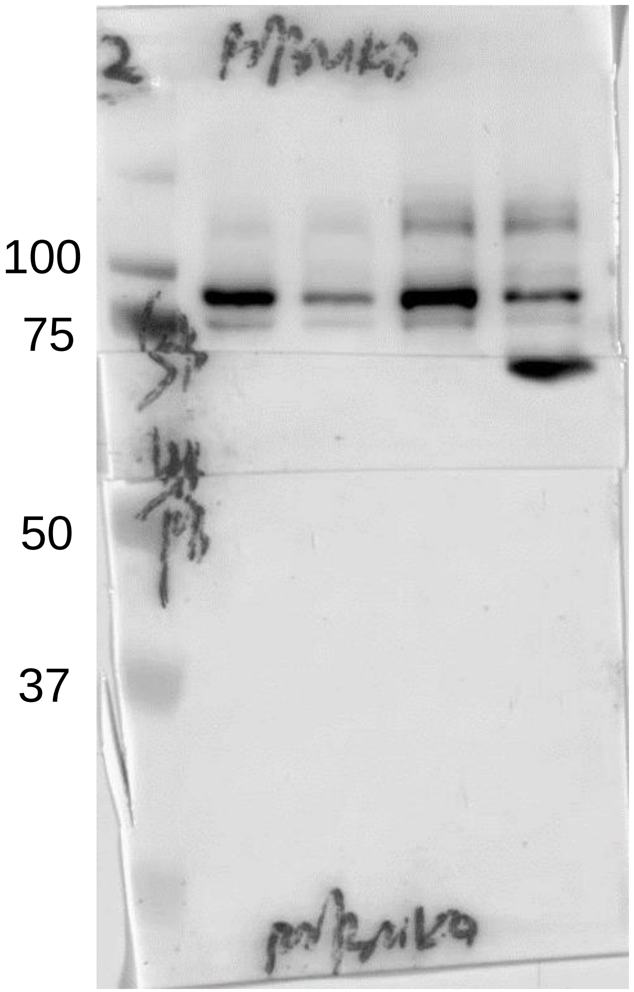

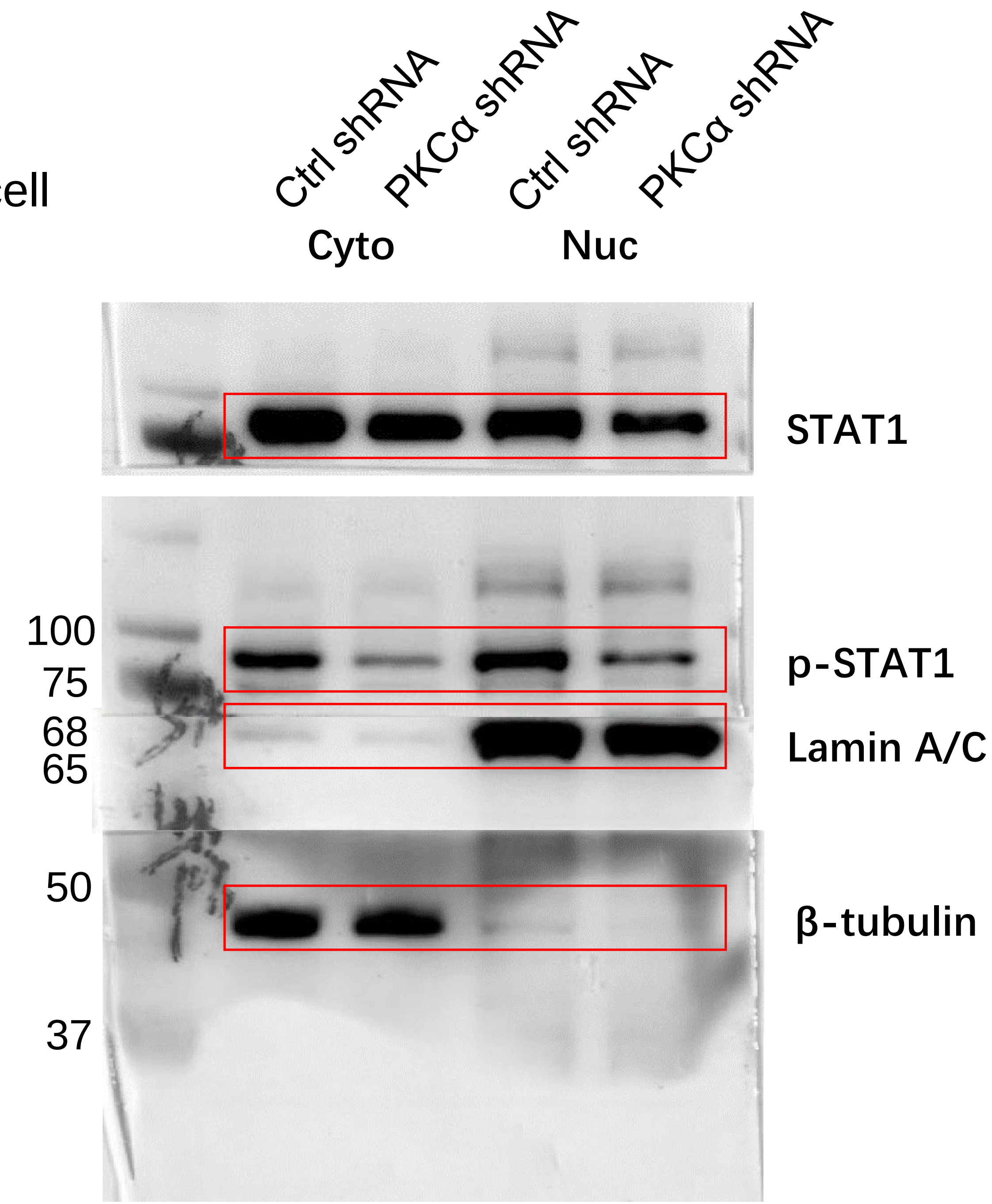

Source Data Fig.7

Cell line: H1975

Fig.7 a

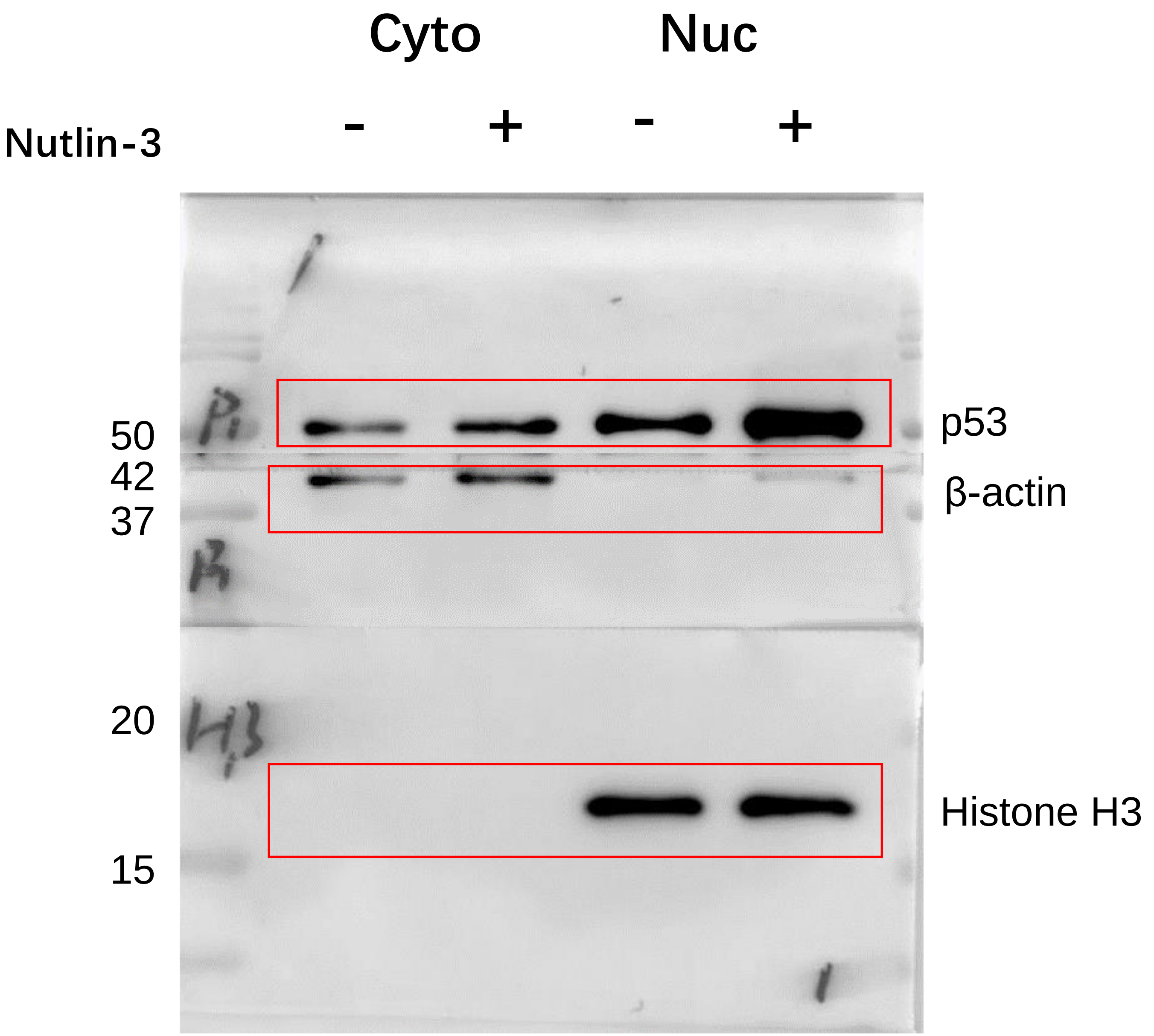

Source Data Fig.7

Cell line: H1975

Fig.7 c

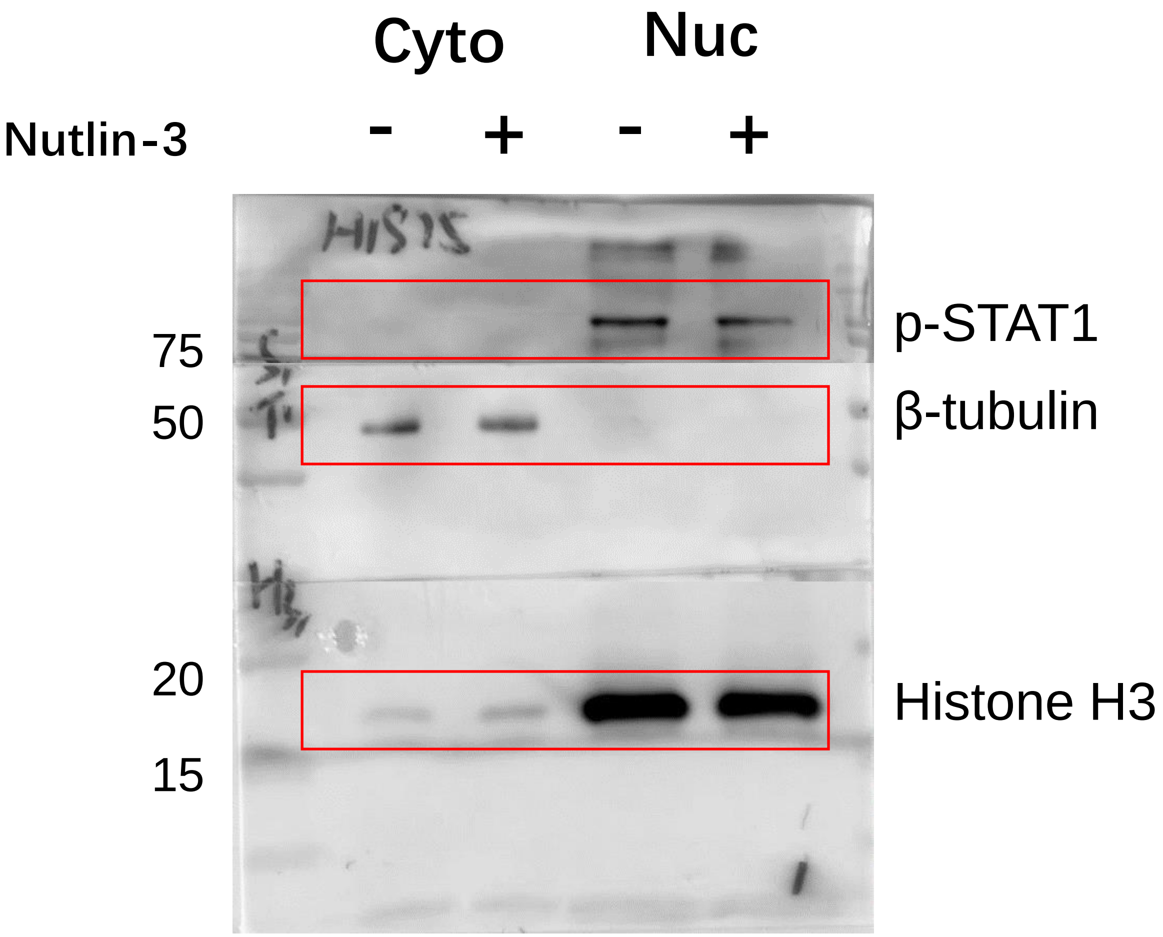

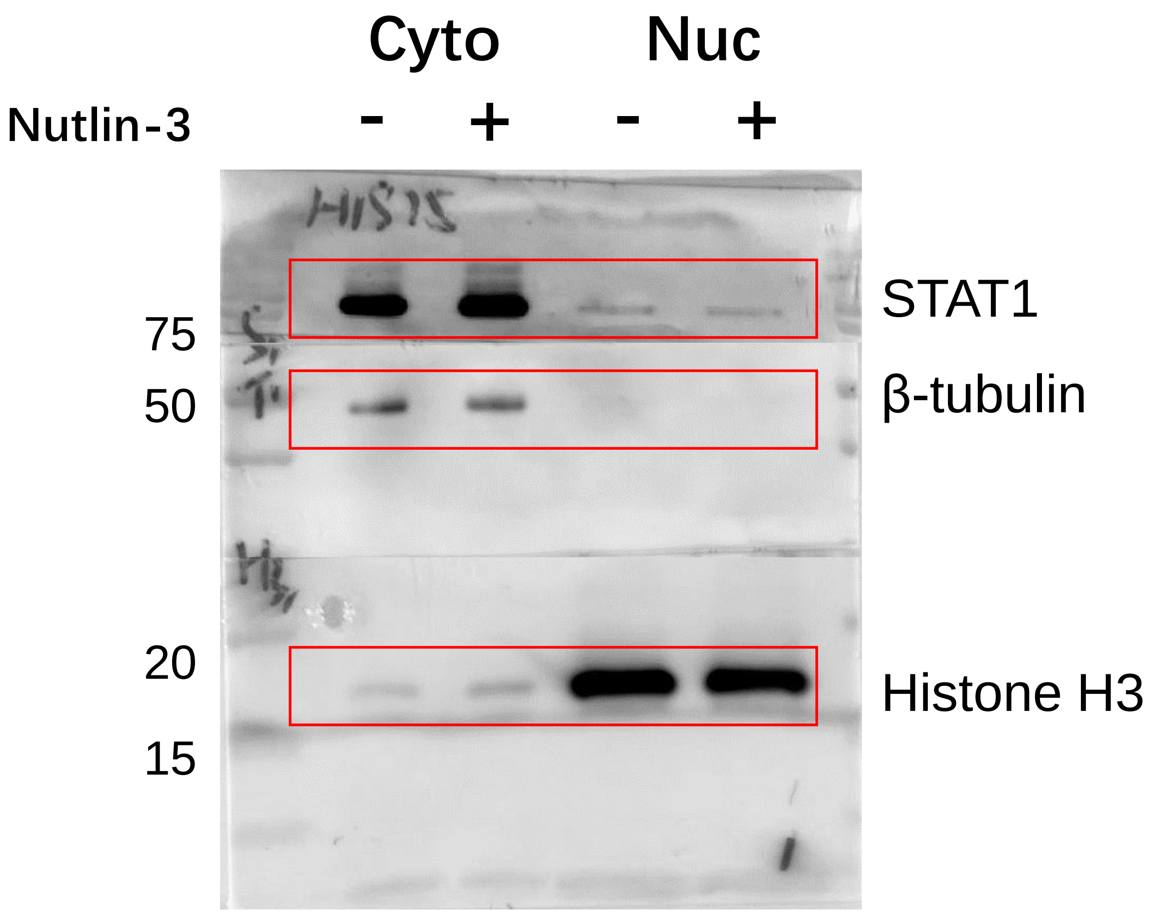

Source Data Fig.7

Cell line: H1975

Fig.7 e

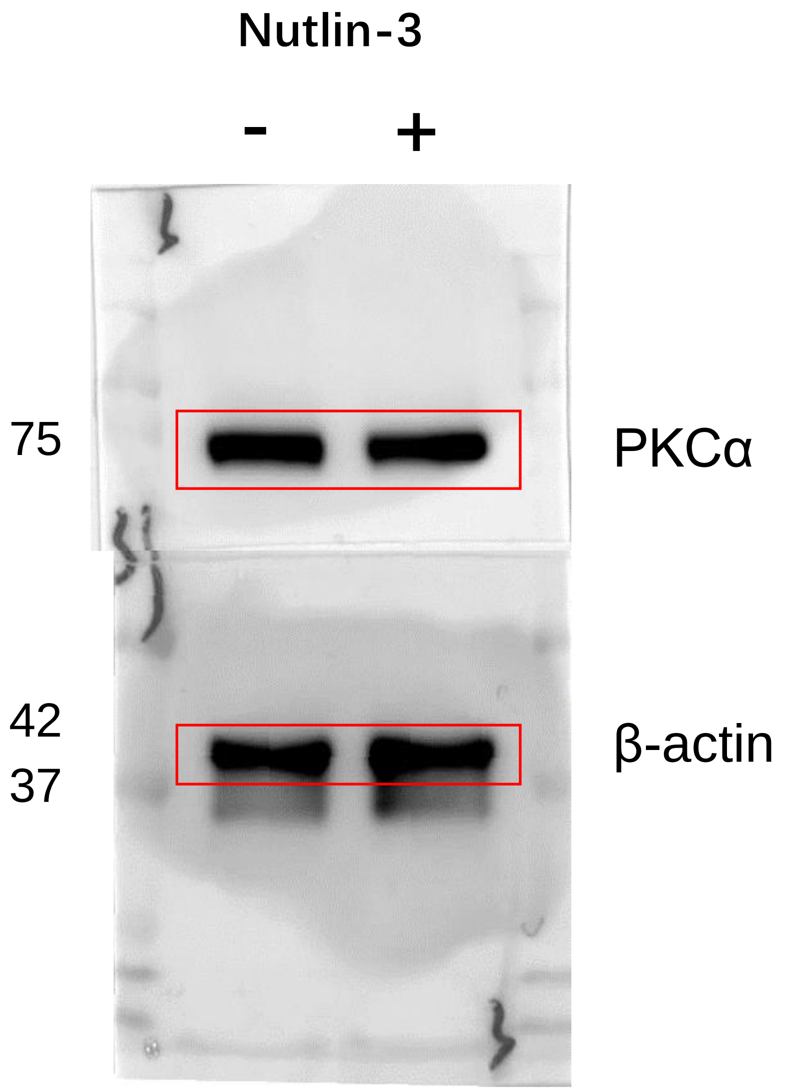

Cell line: H1975

Source Data Fig.7

Fig.7 g

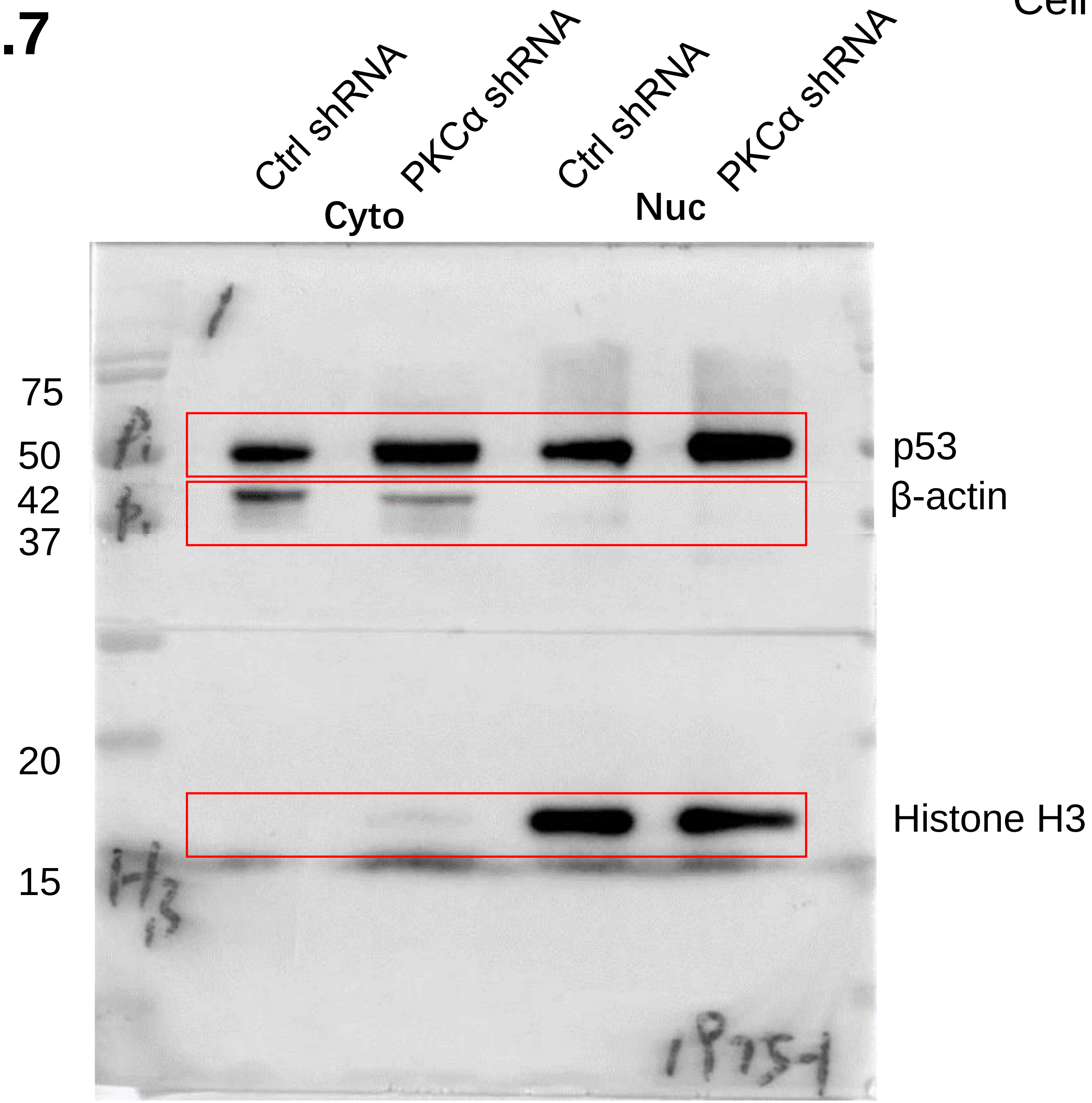

Source Data FigS.2

Cell line: H1299
Whole cell lysates

Fig.S2 a

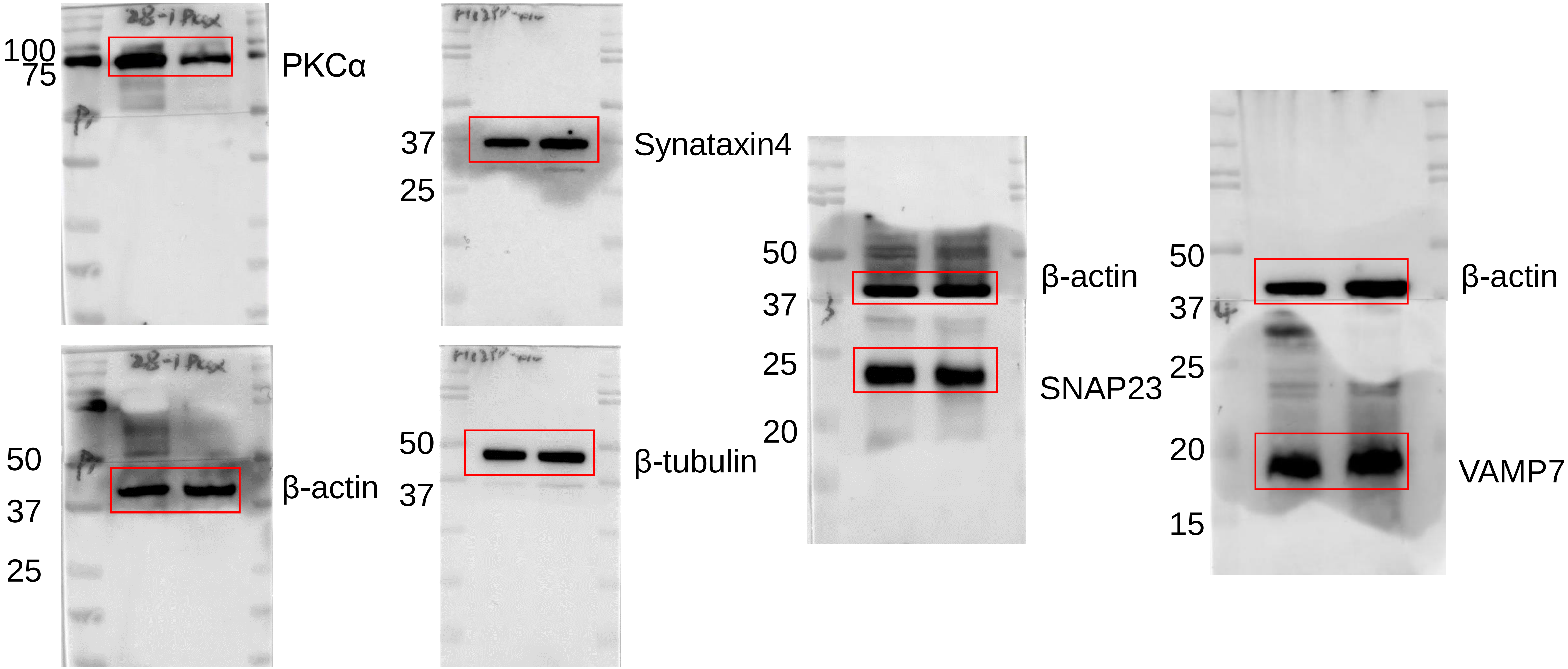

Source Data FigS.2

Cell line: H1299

Whole cell lysates

Fig.S2 c

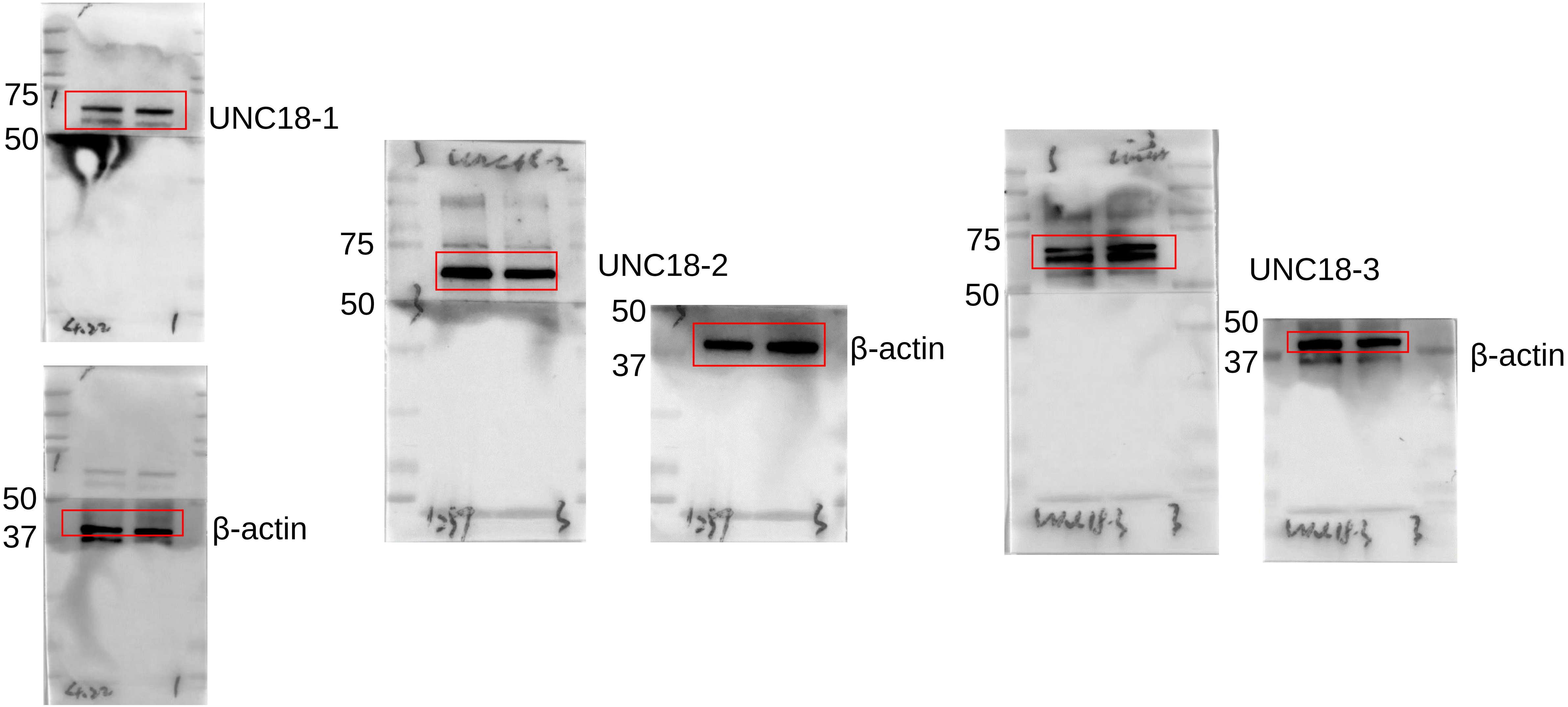

Source Data FigS.3

Fig.S3 a

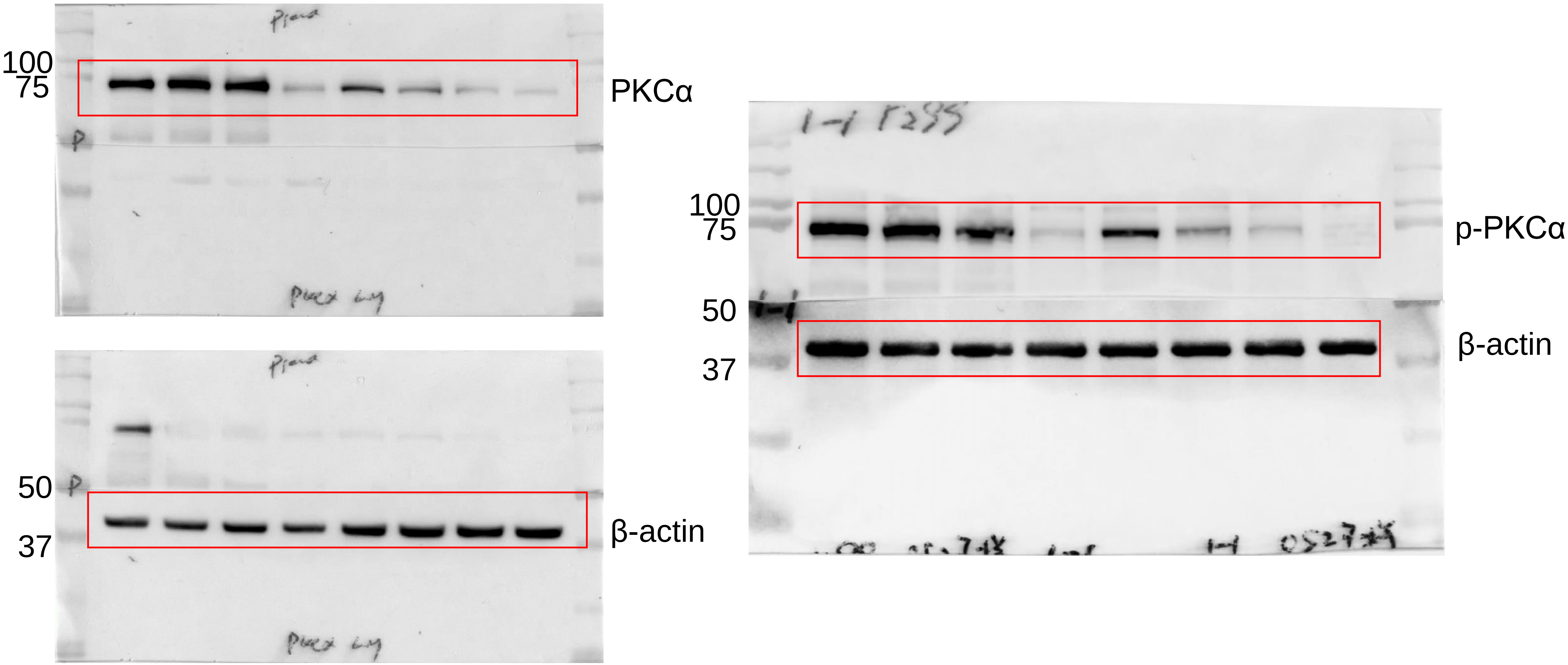

Source Data FigS.3

Fig.S3 c

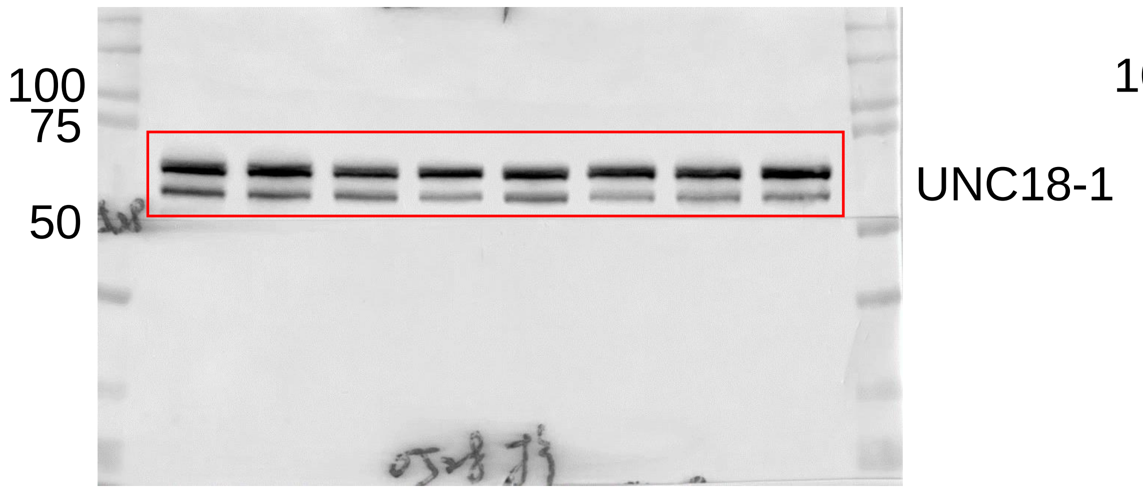

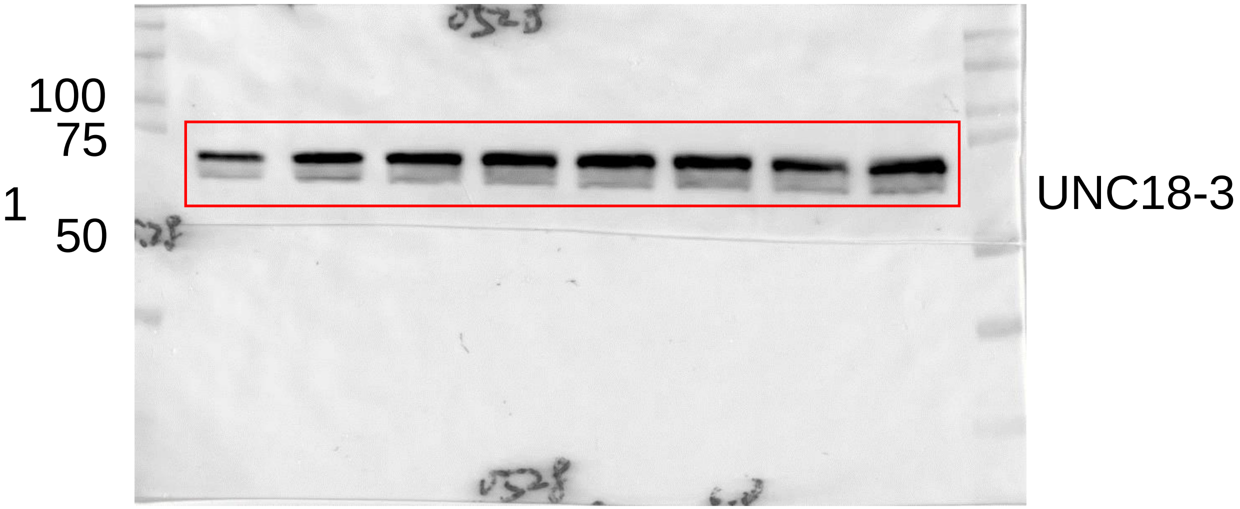

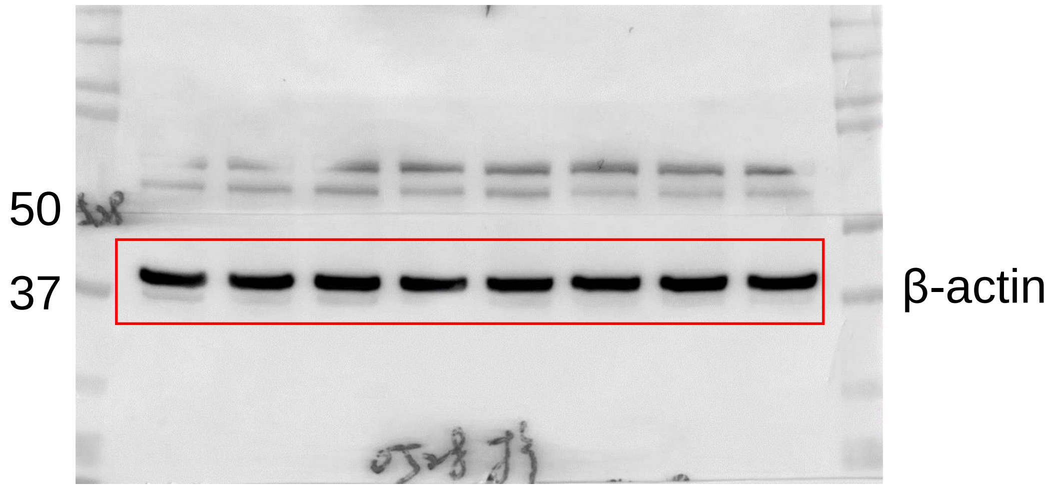

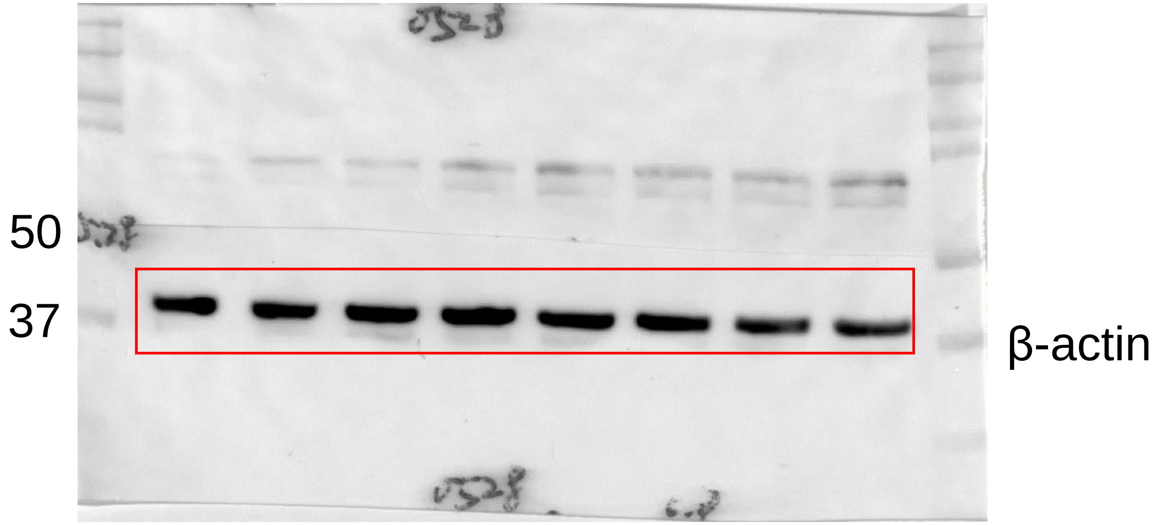

Source Data Fig.S7

Cell line: H1299-p53 R273H cell

Fig.S7 a

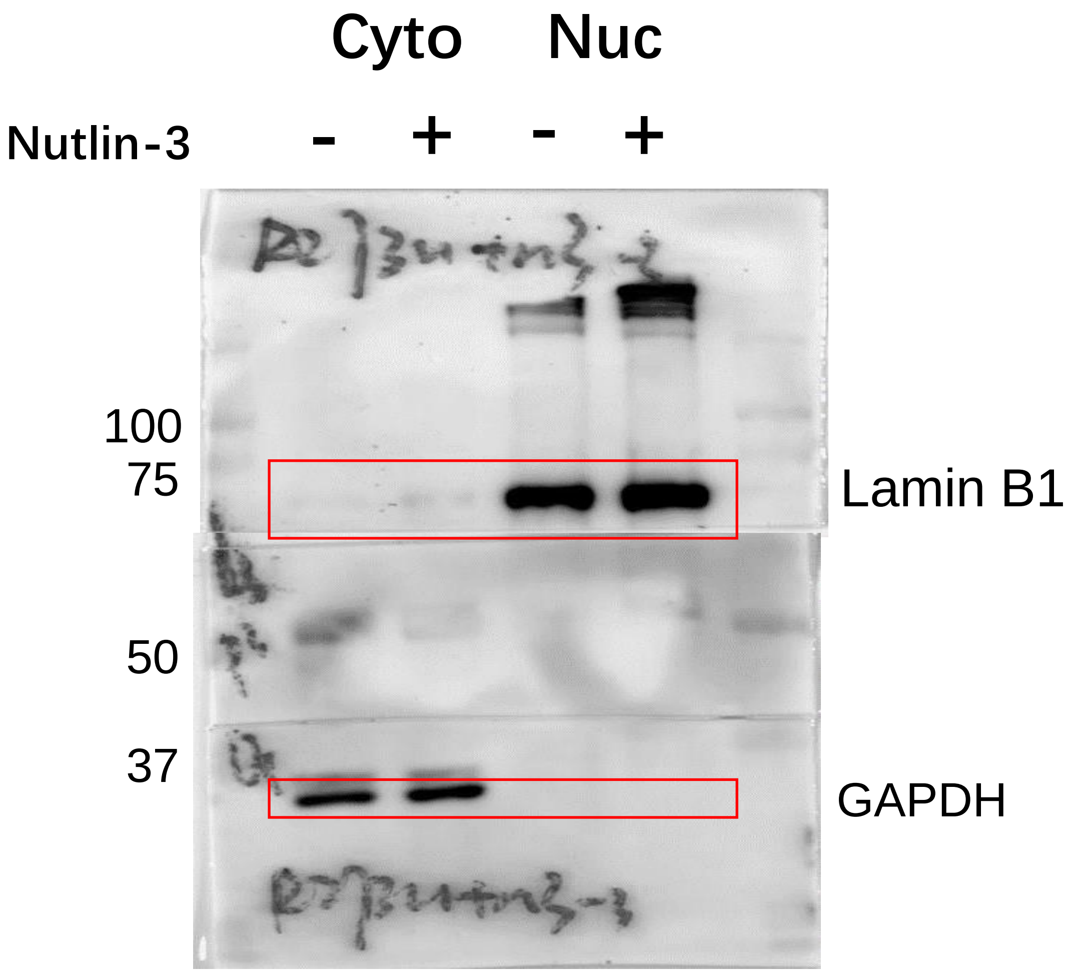

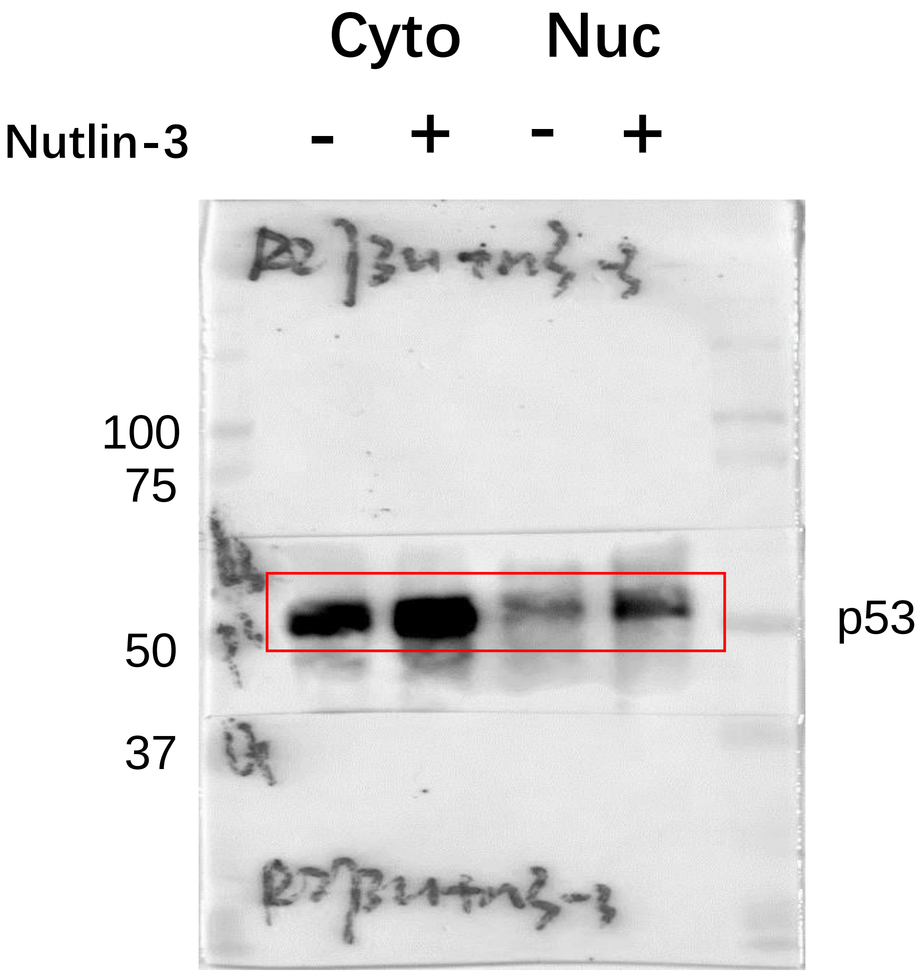

Source Data Fig.S7

Cell line: H1299-p53 R273H cell

Fig.S7 c

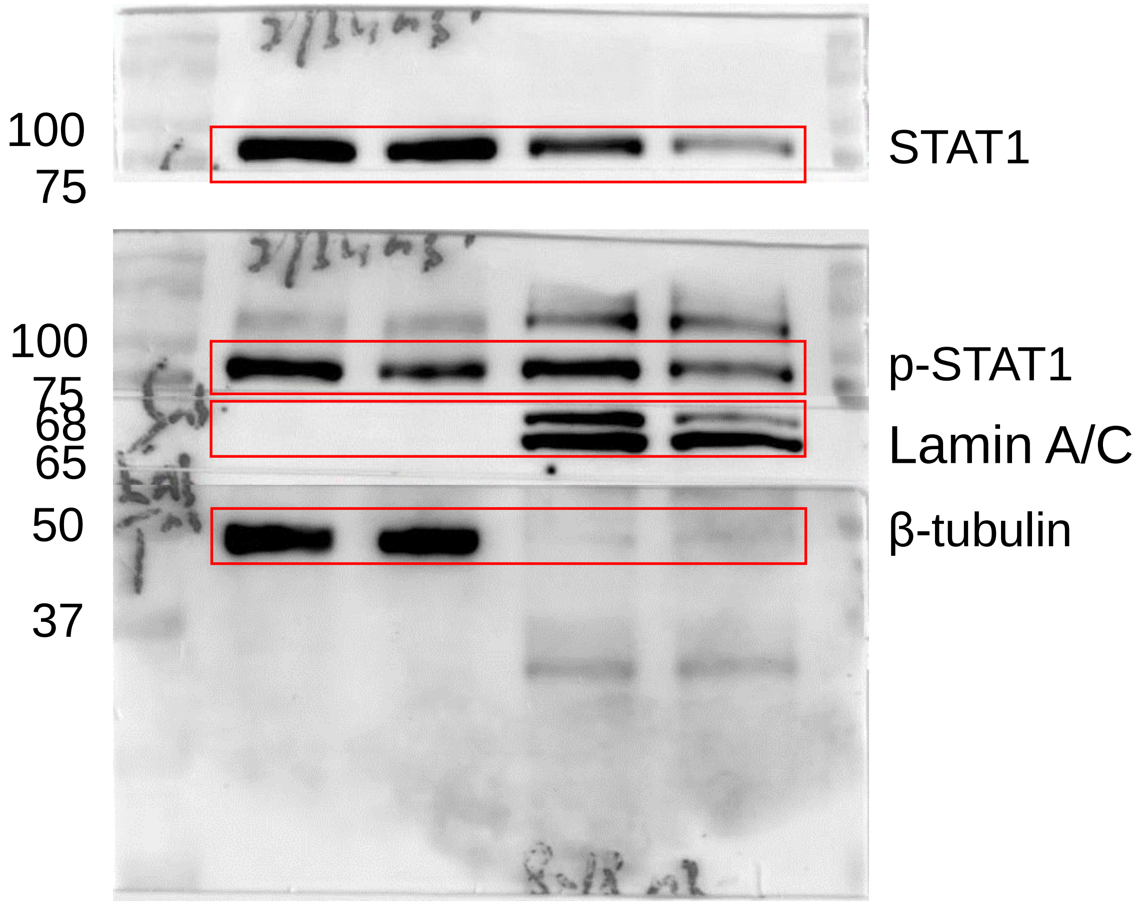

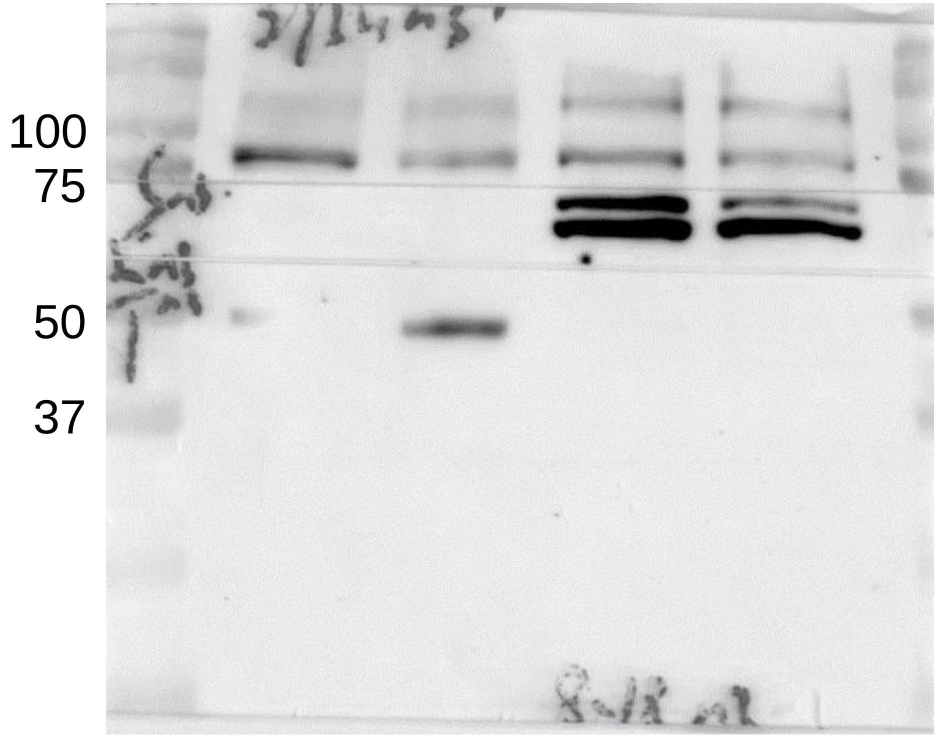

Source Data Fig.S7

Cell line: H1299-p53 R273H cell

Fig.S7 e

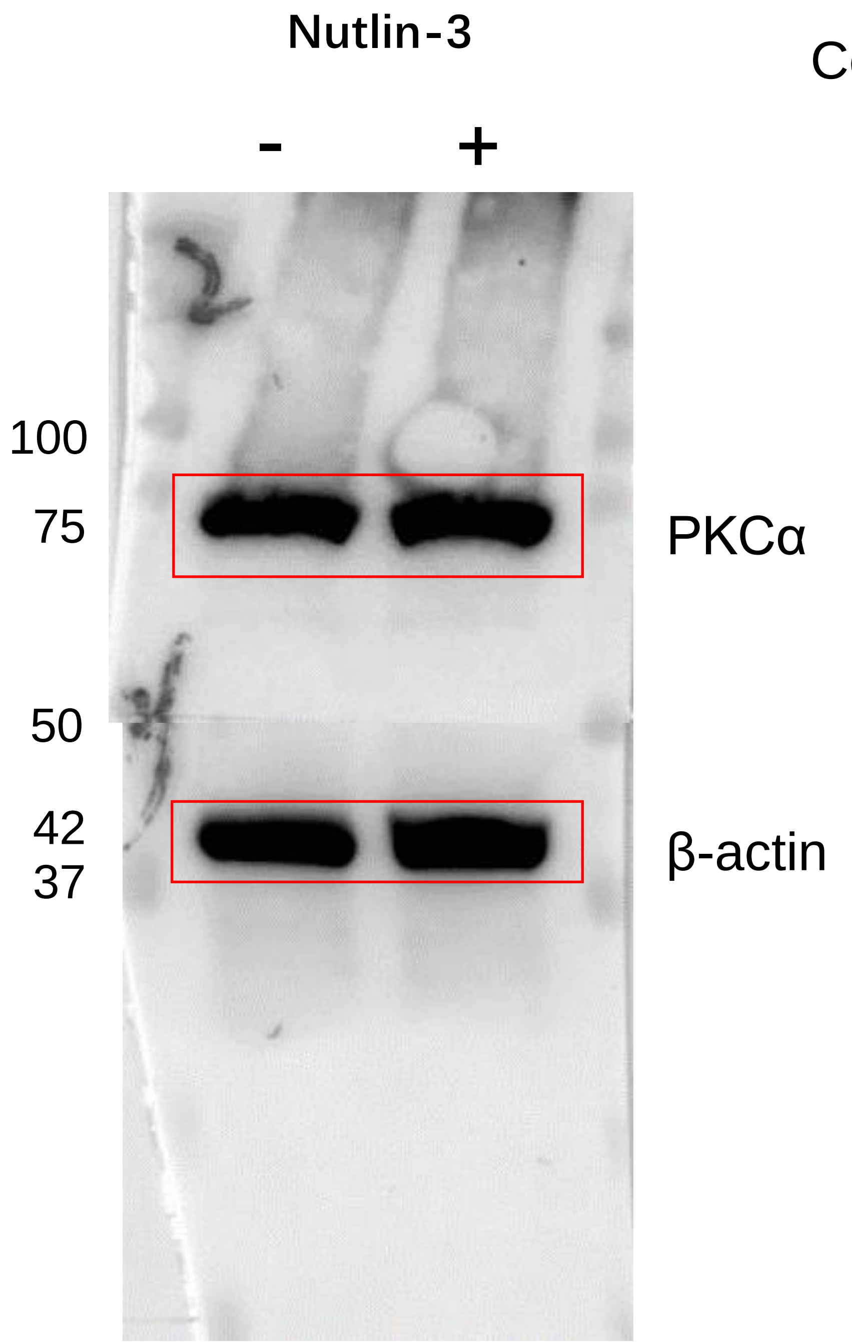

Source Data FigS.7

Cell line: H1299-p53 R273H cell

Fig.S7 g

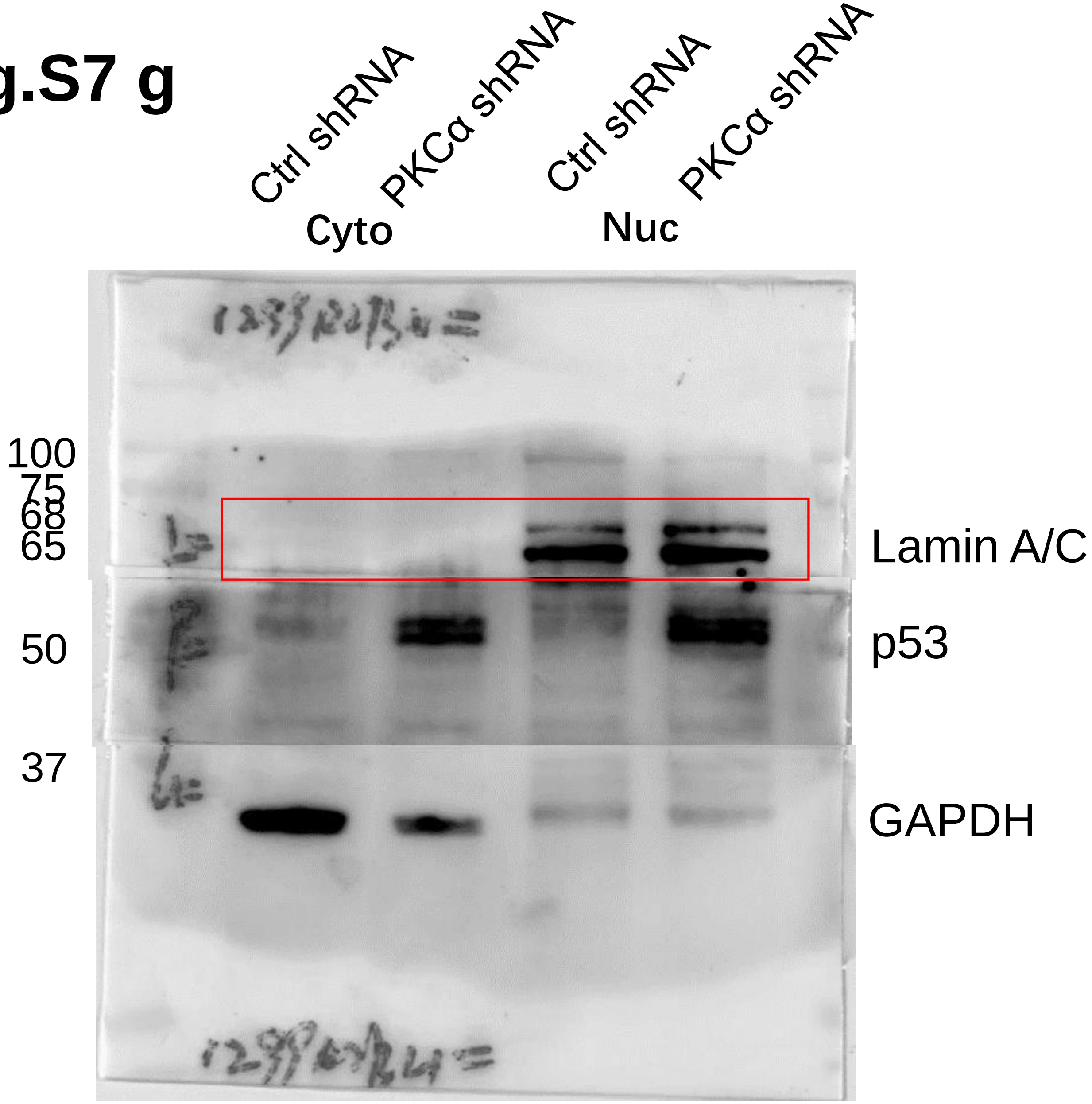

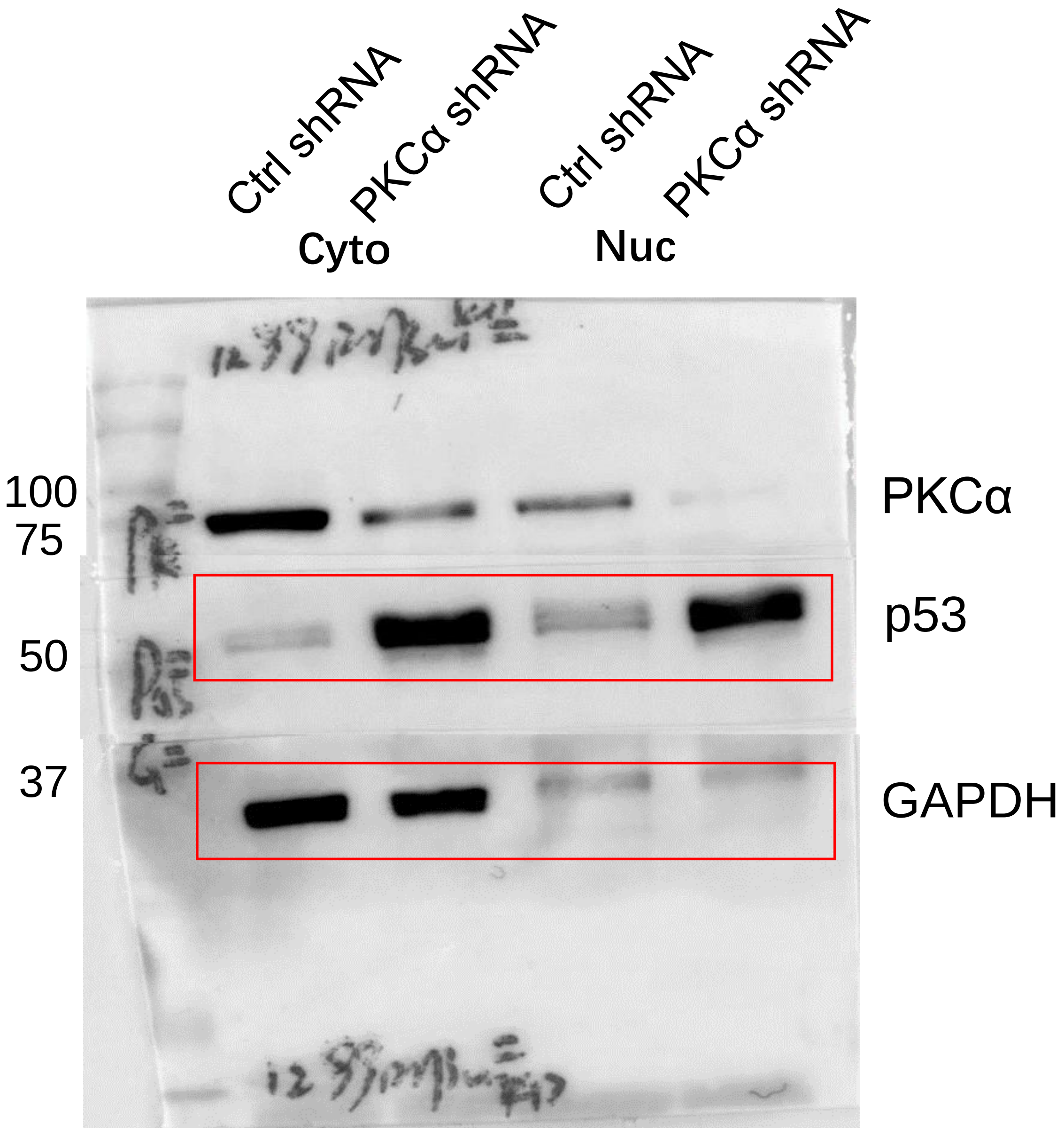

Supplement: Supplementary file 10 — Original Data [file 41419_2025_7341_MOESM10_ESM.pdf]
